# Thioxanthone Synthesis from Thioureas through Double Aryne Insertion into a Carbon-Sulfur Double Bond

**DOI:** 10.1021/acs.orglett.4c04490

**Published:** 2025-01-10

**Authors:** Mayu Kawada, Shinya Tabata, Yukitaka Hoshi, Suguru Yoshida

**Affiliations:** Department of Biological Science and Technology, Faculty of Advanced Engineering, Tokyo University of Science, 6-3-1 Niijuku, Katsushika-ku, Tokyo 125-8585, Japan

## Abstract

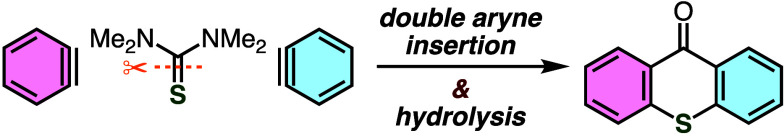

Thioxanthone synthesis from *o*-silylaryl triflates and thioureas is disclosed. Double aryne insertion into the C=S double bond of thioureas and subsequent hydrolysis realized the facile preparation of thioxanthones. A simple reaction procedure and good accessibility of *o*-silylaryl triflates allowed us to synthesize a wide range of highly functionalized thioxanthones.

Thioxanthones are of great importance in various research fields including materials chemistry, pharmaceutical sciences, and catalytic chemistry (Figure 1A).^1−3^ Conventional methods have enabled us to synthesize a range of thioxanthones from *o*-mercaptobenzoic acid and arenes through oxidative *S*-arylation and subsequent cyclization in concentrated sulfuric acid (Figure 1B).^4^ Despite the significance of thioxanthones and continuous efforts to develop novel synthetic methods,^5^ it is not easy to synthesize highly functionalized thioxanthones such as π-extended derivatives. Herein, we disclose a novel method to prepare thioxanthones via double aryne insertion into the carbon=sulfur double bond of thiocarbonyl compounds followed by hydrolysis.

**Figure 1:**
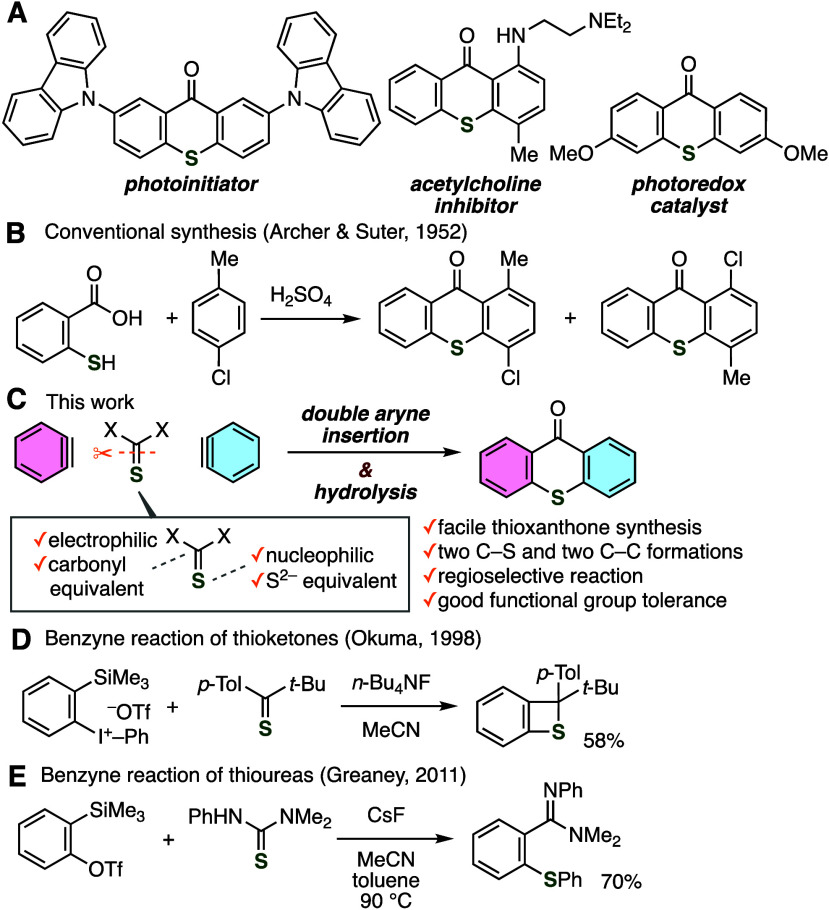
(A) Significant thioxanthones. (B) Conventional thioxanthone synthesis. (C) Overview of this work. (D) Okuma’s study. (E) Greaney’s study.

Aryne insertions into σ- or π-bonds have attracted attention from researchers in diverse disciplines due to their intriguing mechanisms as well as synthetic utility for realizing natural product syntheses.^6^ Recently, we have developed efficient transformations through aryne insertions using organosulfur compounds such as sulfilimines, sulfoxides, sulfoximines, thioalkynes, silyl sulfides, and potassium xanthates.^7^ Inspired by remarkable achievements in aryne insertions of various carbonyl compounds including amides,^8^ we conceived a novel synthetic method for the synthesis of thioxanthones by double aryne insertion of thiocarbonyl compounds such as thiocarbonates (Figure 1C).^9^ Pioneering reactions including [2 + 2] cycloaddition of thioketones^10a^ or amidine synthesis through the C–S cleavage, further *S*-phenylation, and protonation^10b^ indicate the good reactivity of the thiocarbonyl group with aryne intermediates (Figure 1D and E). With these fundamental reactivities of thiocarbonyl compounds and the significance of thioxanthones in mind, we designed a new method to synthesize thioxanthones through ring-opening facilitated by heteroatoms connected to the thiocarbonyl group and further addition–cyclization of the resulting thiolate anions with arynes, followed by hydrolysis leading to the carbonyl formation. The hydrolysis might proceed easily due to the labile xanthone-type acetal structure.^11^ In this sequential reaction pathway using thiocarbonyl compounds, the nucleophilic sulfur serves as an S^2–^ equivalent and the electrophilic carbon serves as a carbonyl source.

After screening thiocarbonyl compounds under the aryne-generating conditions from *o*-(trimethylsilyl)phenyl triflate (**1a**), we succeeded in the synthesis of thioxanthone **3a** through double aryne insertion into the C=S double bond and following hydrolysis (Table 1). First, thiocarbonate **2a** was treated with *o*-silylphenyl triflate **1a** in the presence of cesium carbonate and 18-crown-6, which were chosen for aryne generation and hydrolysis (entry 1). As a result, a complex mixture of products was obtained after an aqueous workup of the resulting mixture, where to our surprise a trace amount of thioxanthone **3a** was observed. Thiocarbonyl compounds **2b**–**2d** having two good leaving groups were not effective for thioxanthone formation (entries 2–4). When using thiocarbamoyl chloride **2e**, the desired thioxanthone **3a** was obtained in low yield (entry 5). We achieved the efficient synthesis of **3a** by treating **1a** and thiourea **2f** with cesium carbonate and 18-crown-6 (entry 6). The thioxanthone formation involved two C–S and two C–C bond formations between thiourea **2f** and two molar amounts of benzyne generated from **1a**, and hydrolysis led to the carbonyl group. It is worthy to note that the corresponding amidines or benzthioamides were not observed. Screening of thiocarbonyl compounds suggests that amino groups improved the reactivity of the thiocarbonyl group. This double aryne insertion into the C=S double bond of thiourea **2f** contrasts the benzyne reaction with *N*,*N*,*N′*,*N′*-tetraethylurea resulting in the insertion into the C–N σ-bond to afford *N*,*N*-diethyl-*o*-(diethylamino)benzamide.^8a^ We accomplished the preparation of thioxanthone **3a** in a good yield without aqueous workup (entry 7). Of note, we achieved the thioxanthone synthesis on a 15 mmol scale (**1a**, 4.5 g) through the practical protocol without aqueous workup. When the benzyne generation conditions were changed, we also synthesized **3a** in moderate to good yields (entries 9 and 10). In contrast, **3a** was not obtained in the case of thioureas **2g** and **2h** having protons at the nitrogen atoms (entries 11 and 12).

**Table 1:**
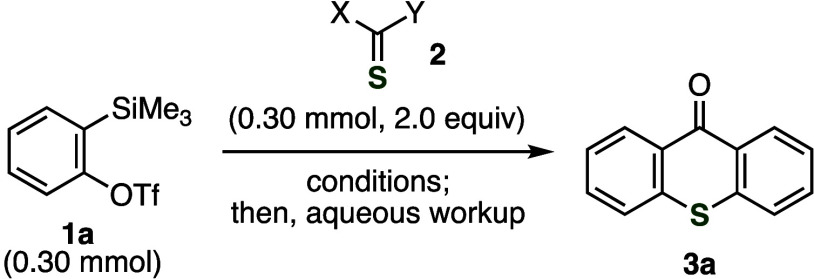

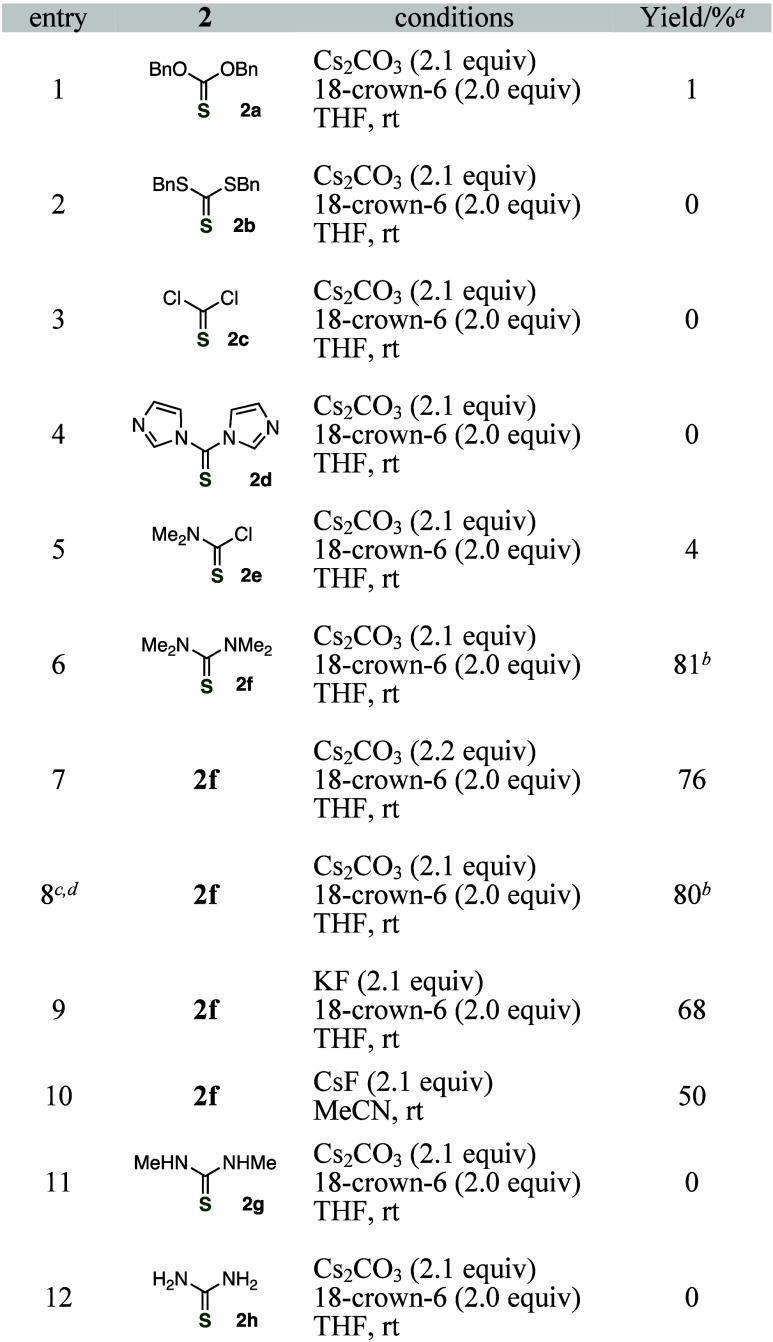
Screening of the Reaction Conditions

a ^1^H NMR yields.

b Isolated yield.

c After filtration of the reaction mixture with a short pad of Celite, removal of the solvent, and silica gel column chromatography, thioxanthone **3a** was obtained without aqueous workup.

d The thioxanthone synthesis was conducted on a 15 mmol scale [**1a** (4.5 g, 15 mmol), **2f** (2.0 g, 15 mmol)].

A wide range of thioxanthones were prepared from *o*-silylaryl triflates possessing various functional groups (Figure 2). For example, 4,5-dimethyl-, 4,5-dimethoxy-, and 4,5-difluorobenzyne smoothly reacted with thiourea **2f** to provide tetrasubstituted thioxanthones **3b**–**3d** in moderate to good yields, leaving methyl, methoxy, and fluoro groups intact (Figure 2A). In particular, the synthesis of tetrasubstituted thioxanthone **3c** was realized on a 5 mmol scale using 4,5-dimethoxybenzyne precursor **1c** (1.8 g). In the case of 3-methoxybenzyne, we succeeded in the facile preparation of thioxanthone **3e** selectively through the hydrolysis with saturated aqueous NH_4_Cl at 60 °C, in which both C–S bond formations took place at the 1-position of benzyne and both C–C formations took place at the 2-position. Also, 5-bromo- or 5-phenyl-3-methoxy-2-(trimethylsilyl)phenyl triflate participated in the selective formation of thioxanthone **3f** or **3g**, respectively, without damaging the bromo or phenyl groups. Since a wide variety of 5-substituted-3-methoxybenzyne precursors can be synthesized via Ir-catalyzed C–H borylation of 3-methoxy-2-(trimethylsilyl)phenyl triflate,^12^ these results indicate that diverse thioxanthones were readily accessible from simple modules such as thiourea **2f**, 3-methoxy-2-(trimethylsilyl)phenyl triflate, and arylboronic acids. It is worth noting that we accomplished the preparation of amino-substituted thioxanthones **3h** and **3i** without side reactions such as *N*-arylation, in which 3-amino-2-(trimethylsilyl)phenyl triflates used as 3-aminobenzyne precursors were easily prepared from 2,6-bis(triflyloxy)phenyl iodide and aminosilanes through the aryne relay manner.^13^ Significant regioselectivities in the synthesis of **3e**–**3i** where no regioisomers were observed should be derived from distorted structures of arynes having electron-negative atoms at the 3-position.^14^ Unfortunately, dibromo-substituted thioxanthone **3j** was not obtained from 2-bromo-6-(trimethylsilyl)phenyl triflate and thiourea **2f**. In the case of thioxanthone synthesis via 4-methylbenzyne, a mixture of regioisomers **3k**, **3k′**, and **3k″** were obtained. Furthermore, we achieved the preparation of pentacyclic compounds **3l**–**3n** via ring-fused benzynes. These results clearly show that a broad range of thioxanthones are available from various *o*-silylaryl triflates with good functional group tolerance based on remarkable progress in aryne chemistry enabling us to generate diverse aryne intermediates.^15,16^ In addition, iminium salt **4a** was synthesized after being treated with aqueous NH_4_Cl at 60 °C when using 3-(4-methoxyphenylthio)-2-(trimethylsilyl)phenyl triflate (**1o**) (Figure 2B).

**Figure 2:**
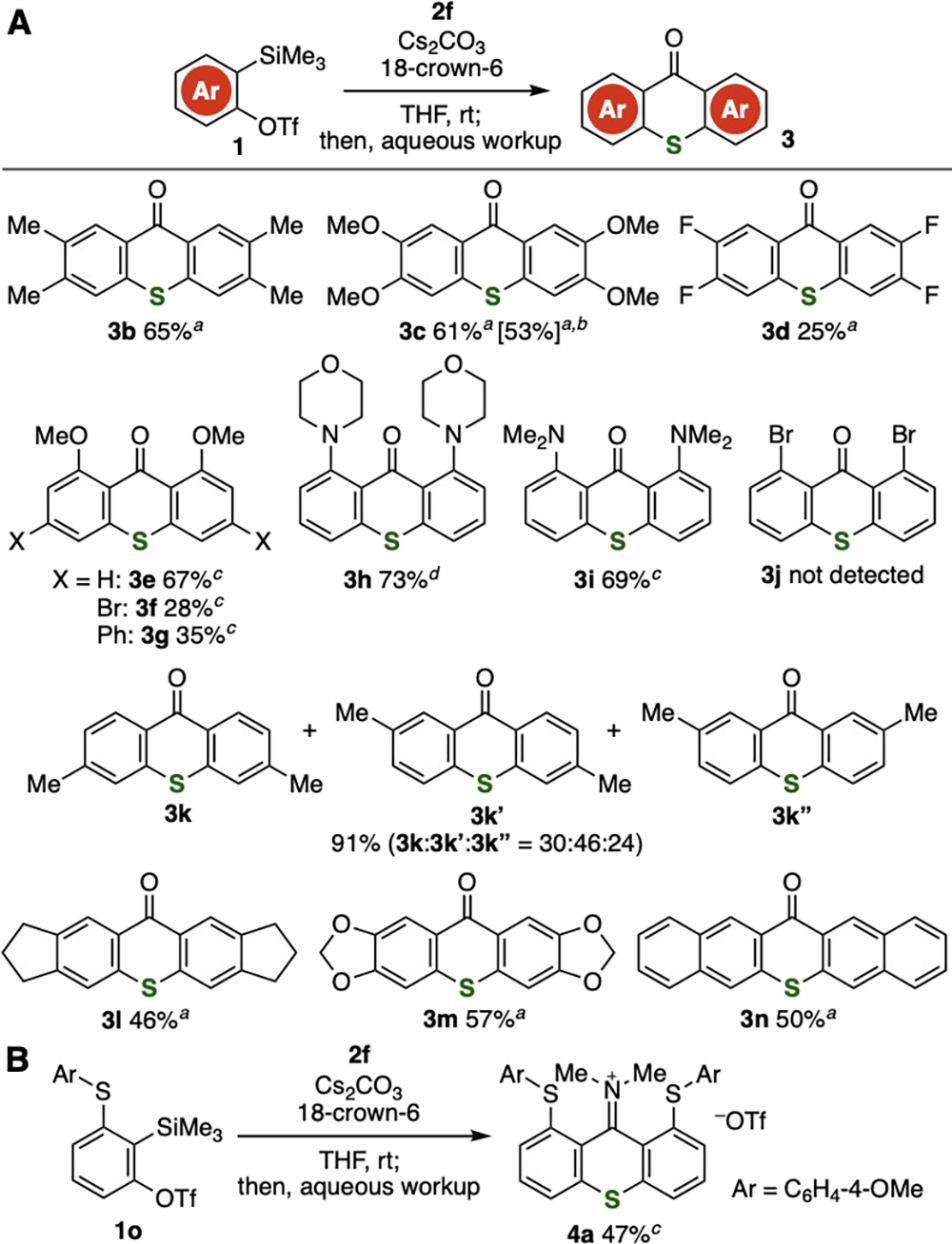
Synthesis of thioxanthones **3** from various *o*-silylaryl triflates **1b**–**1n**. See the Supporting Information for details. ^*a*^Thioxanthones **3** were obtained after filtration of the reaction mixture with a short pad of Celite, removal of the solvent, and silica gel column chromatography. ^*b*^The thioxanthone synthesis was conducted on a 5 mmol scale [**1c** (1.8 g, 5.0 mmol), **2f** (0.66 g, 5.0 mmol)]. ^*c*^Hydrolysis was conducted with saturated aqueous NH_4_Cl in methanol at 60 °C. ^*d*^Hydrolysis was conducted with 1 M aqueous HCl in methanol at 100 °C.

We accomplished the synthesis of unsymmetric thioxanthones using two types of *o*-silylaryl triflates with thiourea **2f** (Figure 3). For instance, thioxanthone **5a** was prepared along with **3e** and **3m** through arynes generated from *o*-silylaryl triflates **1e** and **1l**, respectively.^17^ Also, we realized the synthesis of multisubstituted thioxanthones **5b** and **5c** from *o*-silylaryl triflates **1b**/**1i** and **1c**/**1j**, respectively, in moderate yields. These results clearly show that changing the aryne precursors allowed us to prepare diverse unsymmetric thioxanthones.

**Figure 3:**
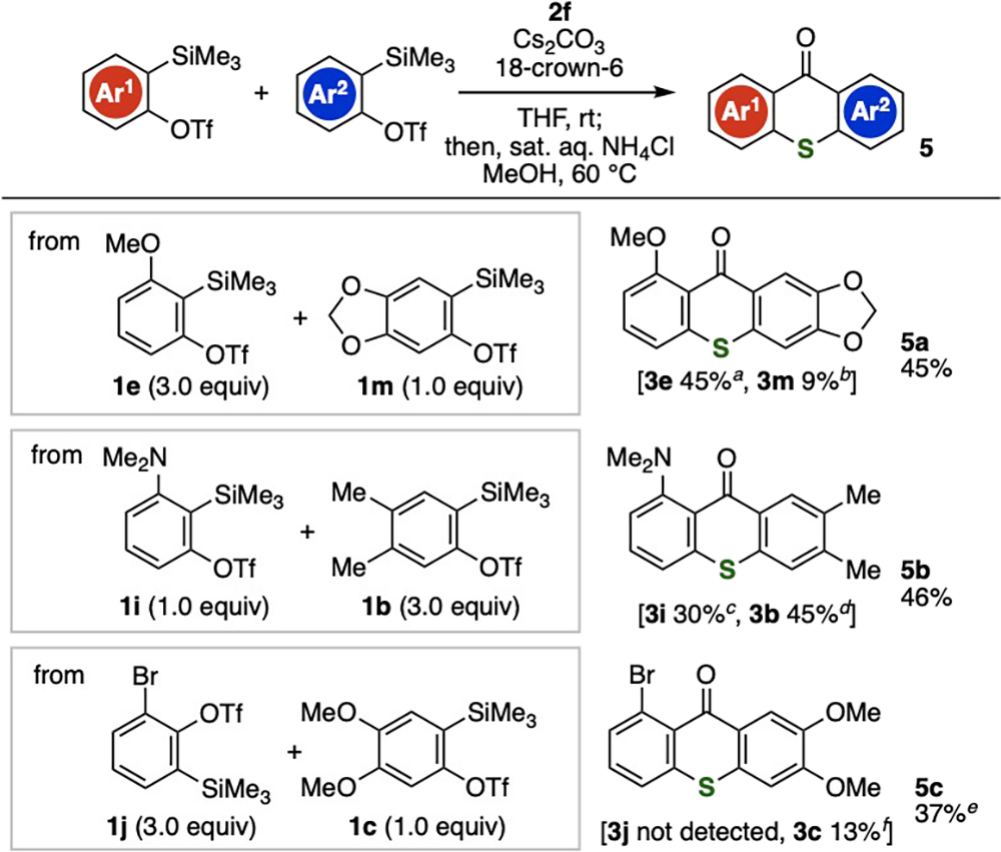
Synthesis of unsymmetric thioxanthones **5a**–**5c**. ^*a*^Yield based on **1e**. ^*b*^Yield based on **1m**. ^*c*^Yield based on **1i**. ^*d*^Yield based on **1b**. ^*e*^Hydrolysis was conducted with 1 M aqueous HCl in methanol at 100 °C. ^*f*^Yield based on **1c**.

To clarify the reaction mechanism, we conducted control experiments for stepwise thioxanthone formation (Figure 4). To our surprise, isolation of iminium salt **4b** was achieved by treating *o*-silylaryl triflate **1e** with slightly bulky thiourea **2i** in the presence of cesium carbonate and 18-crown-6 after purification with silica gel (Figure 4A). While iminium salt **4b** was stable under the basic conditions, we then accomplished thioxanthone synthesis from iminium salt **4b** by boiling with hydrochloric acid. Then, we analyzed the products from *o*-silylaryl triflate **1e** and thiourea **2f** after a quick aqueous workup and purification with silica gel (Figure 4B). As a result, iminium salt **4c** was obtained as a mixture with 18-crown-6 along with a small amount of thioxanthone **3e**. This result also suggests that carbonyl oxygen was derived from water in the workup. The hydrolysis was promoted by cesium ions (Figure 4C).^18^ Indeed, after the removal of inorganic salts by aqueous workup of the reaction mixture, the yields of **3a** by the following hydrolysis with NH_4_Cl at 60 °C were obviously increased by the addition of cesium triflate or cesium carbonate. These results support that the synthesis of thioxanthone **3e** involves double aryne insertion affording iminium salt **4c** as an intermediate and the following hydrolysis took place with saturated aqueous NH_4_Cl at 60 °C in the presence of cesium ions.

**Figure 4:**
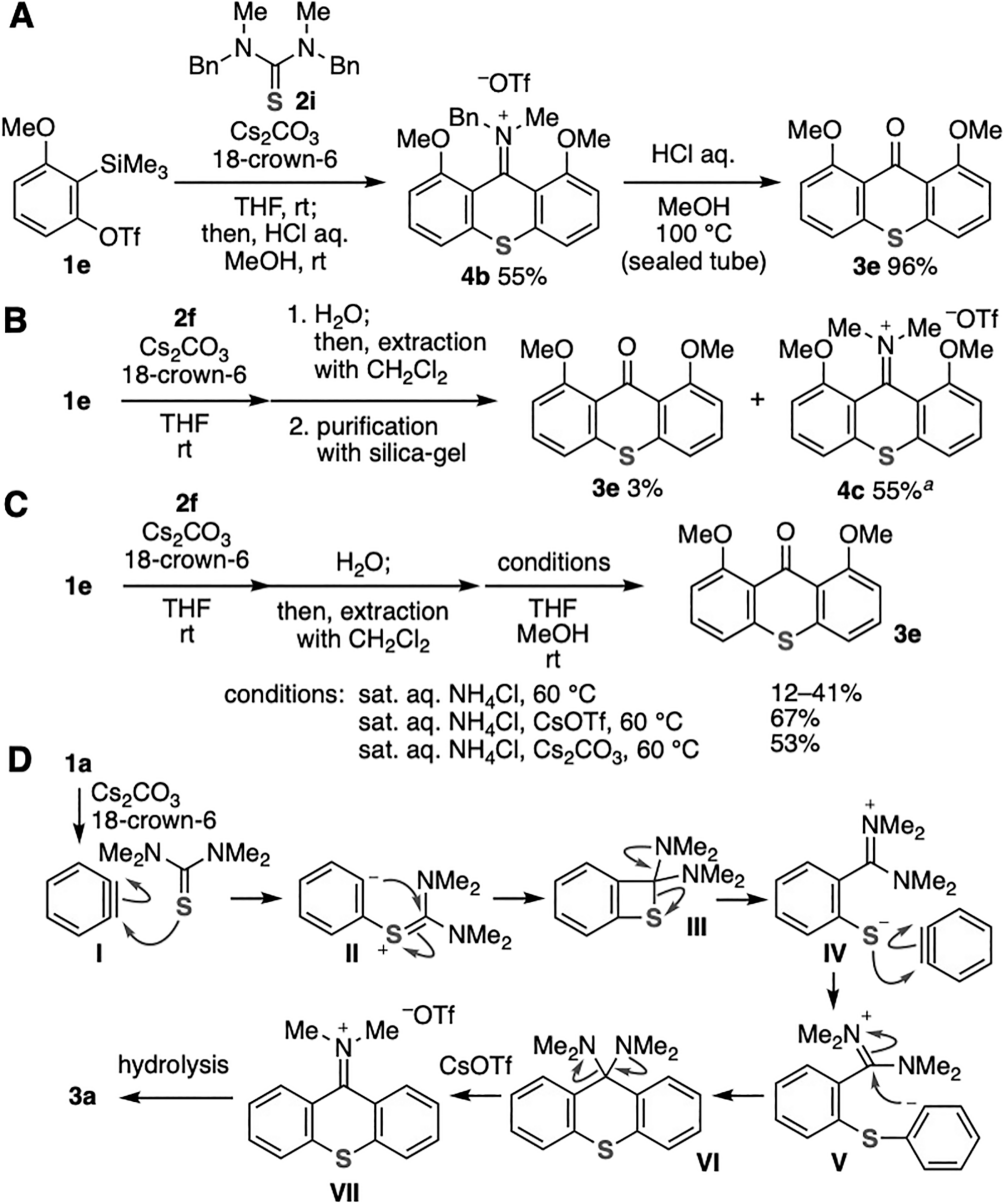
(A) Isolation and hydrolysis of iminium salt **4b**. (B) Control experiments using **1e** and **2f**. (C) Control experiments for the hydrolysis of **4c**. (D) Plausible reaction mechanism. ^*a*^Iminium salt **4c** was obtained as a mixture with 18-crown-6.

A plausible reaction mechanism on the basis of control experiments is shown in Figure 4D. The reaction was triggered by formal [2 + 2] cycloaddition of benzyne (**I**) with thiourea **2f** leading to four-membered ring intermediate **III**. Then, the ring-opening of **III** would generate amidinium intermediate **IV** having a thiolate moiety, which reacts with benzyne (**I**) to provide iminium intermediate **VII** via addition, cyclization, and C–N cleavage. Following hydrolysis of the iminium intermediate **VII** would afford thioxanthone **3a**, in which the hydrolysis of bulky iminium intermediates took place under acidic conditions in the presence of cesium ions.

The novel method to construct a thioxanthone scaffold through arynes enabled us to synthesize various π-extended thio xanthones (Figure 5A).^19^ For example, dibenzofuran **1p** having a triflyloxy and a silyl group was successfully prepared from 3-hydroxydibenzofuran **6** by electrophilic iodination, *O*-silylation, *C*-silylation by the retro-Brook rearrangement, and triflylation.^20^ The treatment of **1p** with thiourea **2f** in the presence of cesium carbonate and 18-crown-6 furnished heptacyclic compound **3o** possessing a thioxanthone skeleton fused with two benzofuran rings. In the formation of **3o**, both C–S formations took place at the 3-position of dibenzofuran-type aryne **VIII**, and both C–C formations took place at the 4-position. Since recent aryne chemistry allowed us to utilize diverse fused arynes involving hetarynes, a wide variety of π-extended thioxanthones will be synthesized from *o*-silylaryl triflates and thiourea **2f**.

**Figure 5:**
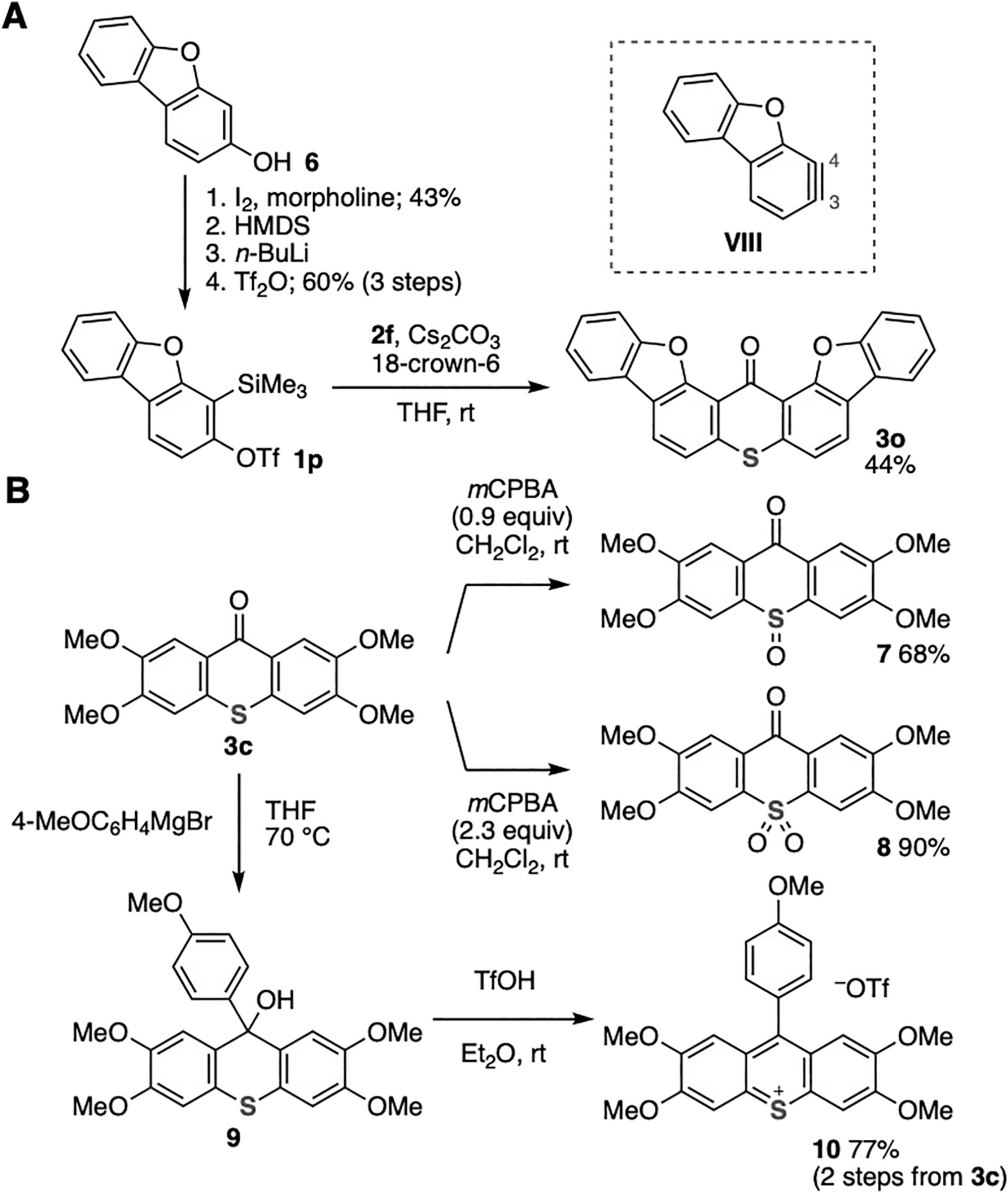
(A) Synthesis of thioxanthone **3o**. (B) Transformations of **3c**. See the Supporting Information for details.

We showcased the preparation of thioxanthone derivatives by modifications of the thioxanthone ring of **3c** through various transformations (Figure 5B, upper). Indeed, the syntheses of sulfoxide **7** and sulfone **8** were achieved in good yields through oxidation by changing the amount of *m*CPBA.^21^ Furthermore, a Grignard reaction of **3c** and subsequent dehydration successfully led to the formation of highly functionalized thiopyrylium salt **10** (Figure 5B, lower).^22^ Indeed, treatment of thioxanthone **3c** with 4-methoxyphenylmagnesium bromide in hot THF followed by dehydration with triflic acid provided pentamethoxy-substituted thiopyrylium salt **10** in a good yield.

Photophysical properties of thioxanthones **3a**, **3c**, **3n**, **3o**, and **8** and thiopyrylium salt **10** show that structural differences such as substituents or π-extension significantly affect absorption and emission spectra (Figure 6). In particular, a clear red shift was observed in the emission spectra by changing the oxidation state of sulfur or π-extension. Thioxanthone skeletons are promising not only as functional molecules including fluorescent molecules and photocatalysts but also as key intermediates for the preparation of various derivatives such as thiopyrylium salts. Therefore, thioxanthone synthesis by double aryne insertion would enable us to study a variety of newly designed thioxanthone derivatives and thiopyryliums.

**Figure 6:**
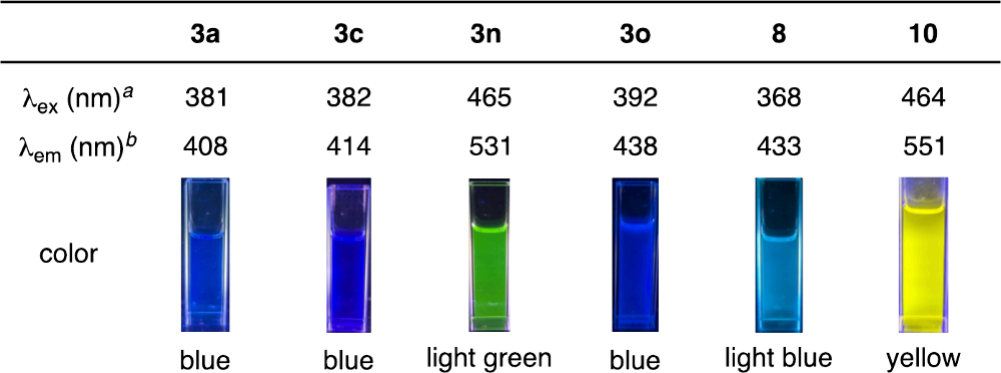
Fluorescent properties of the thioxanthone derivatives. See the Supporting Information for details. ^*a*^Wavelength of the maximum absorption. ^*b*^Wavelength of the maximum fluorescent intensity.

In summary, we have developed a novel thioxanthone synthesis from *o*-silylaryl triflates and thioureas by the double aryne insertion of thioureas. Thioxanthone formation by the domino pathway was realized by thioureas having nucleophilic sulfur as the S^2–^ equivalent and electrophilic carbon as a carbonyl source. A broad range of highly functionalized thioxanthones were successfully prepared due to the good functional group tolerance. Further studies such as the synthesis of various functionalized thioxanthone derivatives involving unsymmetric thioxanthones, detailed theoretical analysis by DFT calculations, and application to synthesizing functional materials including thioxanthene-type molecular motors are ongoing in our research group.

## Data Availability

The data underlying this study are available in the published article and its Supporting Information.

## Supporting Information Available

The Supporting Information is available free of charge on the ACS Publications Web site. (PDF). The Supporting Information is available free of charge at https://pubs.acs.org/doi/10.1021/acs.orglett.4c04490.

Experimental procedures and characterization for new compounds, including NMR spectra (PDF)
