# Supplementary material for: Thioxanthone Synthesis from Thioureas through Double Aryne Insertion into a Carbon-Sulfur Double Bond
Source: Org Lett. 2025 Jan 10;27(3):827–32. doi: 10.1021/acs.orglett.4c04490 (PMC11773562; doi:10.1021/acs.orglett.4c04490)

Supporting Information

Thioxanthone Synthesis from Thioureas through Double Aryne Insertion into Carbon-Sulfur Double Bond

Mayu Kawada, Shinya Tabata, Yukitaka Hoshi, and Suguru Yoshida

Department of Biological Science and Technology, Faculty of Advanced Engineering, Tokyo University of Science, 6-3-1 Niijuku, Katsushika-ku, Tokyo 125-8585

Contents

General Information S1
Structures of Aryne Precursors 1 S2
Experimental Procedures S3
Absorption and fluorescent properties and spectra S10
Characterization Data of New Compounds S12
References for Supporting Information S18
^{1}H and ^{13}C NMR Spectra of Compounds S19
^{1}H NMR Spectra of Reported Compounds S45

General Information

All reactions were performed with dry glassware under atmosphere of argon, unless otherwise noted. Analytical thin-layer chromatography (TLC) was performed on precoated (0.25 mm) silica-gel plates (Merck Chemicals, Silica Gel 60 F254, Cat. No. 1.05715. Column chromatography was conducted using silica-gel (Kanto Chemical Co., Inc., Silica Gel 60, spherical, particle size 40–50 μm, Cat. No. 37562-85). Melting points (Mp) were measured on an OptiMelt MPA100 (Stanford Research Systems), and are uncorrected. ^{1}H NMR spectra were obtained with a Bruker AVANCE 400 spectrometer at 400 MHz. ^{13}C NMR spectra were obtained with a Bruker AVANCE 400 spectrometer at 101 MHz. ^{19}F NMR spectra were obtained with a Bruker AVANCE 400 spectrometer at 377 MHz. All NMR measurements were carried out at 25 ºC. $CDCl_3$ (Kanto Chemical Co. Inc., Cat. No. 07663-23) was used as a solvent for obtaining NMR spectra. Chemical shifts (δ) are given in parts per million (ppm) downfield from $(CH_3)_4Si$ (δ 0.00 for ^{1}H NMR in $CDCl_3$) or the solvent peak (δ 77.0 for ^{13}C NMR in $CDCl_3$; δ 53.5 for ^{13}C NMR in CD_2Cl_2) as an internal reference or α,α,α-trifluorotoluene (δ –63.0 ppm for ^{19}F NMR in $CDCl_3$) as an external standard with coupling constants (*J*) in hertz (Hz). The abbreviations s, d, t, and m signify singlet, doublet, triplet, and multiplet, respectively. IR spectra were measured on a Shimadzu IRSpirit spectrometer with the absorption band given in cm^{-1}. High-resolution mass spectra (HRMS) were measured on a JEOL JMS-T100CS "AccuTOF CS" mass spectrometer under positive electrospray ionization (ESI^+) conditions or negative electrospray ionization (ESI^-). The absorbance spectra (UV/Vis) and fluorescence spectra (FL) were measured with a JASCO UV-750 spectrophotometer and a JASCO FP-8250 spectrofluorophotometer, respectively, at 25 °C using a quartz cuvette (10 mm light path). Recycling preparative HPLC was conducted using a YMC-GPC T2000 (600 mm × 20 φ) column (YMC Co., Ltd.) with a recycling preparative HPLC system (SHIMADZU, eluent: $CHCl_3$). Fluorescence quantum yields were estimated by using 9,10-diphenylanthracene (9,10-DPA) in cyclohexane (ϕ = 0.95) or uranine in ethanol (ϕ = 0.97) as a standard.

Unless otherwise noted, materials obtained from commercial suppliers were used without further purification. 4,5-Dimethyl-2-(trimethylsilyl)phenyl trifluoromethanesulfonate (**1b**),[S1] 4,5-dimethoxy-2-(trimethylsilyl)phenyl trifluoromethanesulfonate (**1c**),[S2] 4,5-difluoro-2-(trimethylsilyl)phenyl trifluoromethanesulfonate (**1d**),[S1] 3-methoxy-2-(trimethylsilyl)phenyl trifluoromethanesulfonate (**1e**),[S3] 5-bromo-3-methoxy-2-(trimethylsilyl)phenyl trifluoromethanesulfonate (**1f**),[S4] 5-methoxy-4-(trimethylsilyl)-[1,1'-biphenyl]-3-yl trifluoromethanesulfonate (**1g**),[S4] 3-morpholino-2-(trimethylsilyl)phenyl trifluoromethanesulfonate(**1h**),[S5] 3-(dimethylamino)-2-(trimethylsilyl)phenyl trifluoromethanesulfonate (**1i**),[S5] 3-bromo-2-(trimethylsilyl)phenyl trifluoromethanesulfonate (**1j**),[S6] 6-(trimethylsilyl)-2,3-dihydro-1*H*-inden-5-yl trifluoromethanesulfonate (**1l**),[S7] 6-(trimethylsilyl)benzo[*d*][1,3]dioxol-5-yl trifluoromethanesulfonate (**1m**),[S7] 3-((4-methoxyphenyl)thio)-2-(trimethylsilyl)phenyl trifluoromethanesulfonate (**1o**),[S8] benzyl phenyl carbonotrithioate (**2b**),[S9] 1,3-dibenzyl-1,3-dimethylthiourea (**2i**),[S10] and cesium trifluoromethanesulfonate[S11] were prepared according to the reported methods.

Structures of Aryne Precursors 1

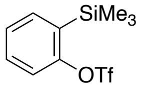

1a

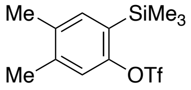

1b

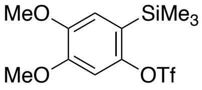

1c

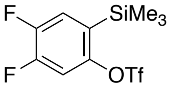

1d

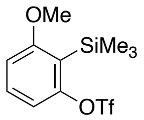

1e

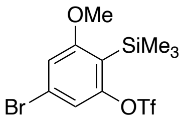

1f

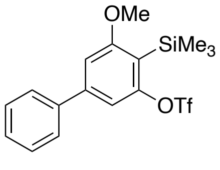

1g

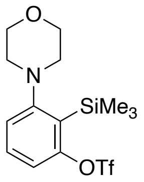

1h

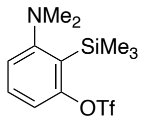

1i

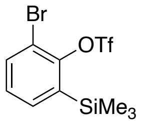

1j

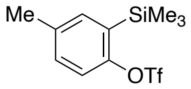

1k

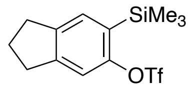

1l

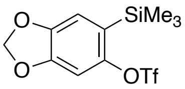

1m

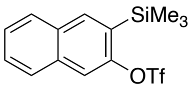

1n

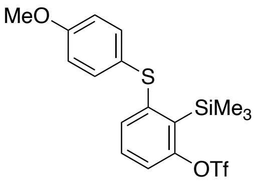

1o

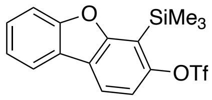

1p

Experimental Procedures

A typical procedure for the synthesis of thioxanthones

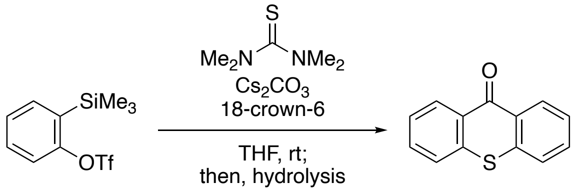

To a solution of 2-(trimethylsilyl)phenyl trifluoromethanesulfonate (**1a**) (89.5 mg, 0.300 mmol) and 1,1,3,3-tetramethylthiourea (**2f**) (39.7 mg, 0.298 mmol, 2.0 equiv) in THF (1.8 mL) in a 5 mL screw-top V-vial® with a solid-top cap (Sigma-Aldrich, Cat. No. Z115118) were added Cs_2CO_3 (205 mg, 0.629 mmol, 2.1 equiv) and 18-crown-6 (160 mg, 0.606 mmol, 2.0 equiv) at room temperature. After stirring for 24 h at the same temperature, to the mixture was added water (2 mL + 10 mL). The mixture was extracted with EtOAc (10 mL × 3). The combined organic extract was washed with brine (5 mL), and then dried with Na_2SO_4. After filtration, the filtrate was concentrated under reduced pressure. The residue was purified by preparative TLC (*n*-hexane/AcOEt = 4/1) to give 9*H*-thioxanthen-9-one (**3a**) (25.7 mg, 0.121 mmol, 81%) as a colorless solid.

A typical procedure for the synthesis of thioxanthones without aqueous workup

To a solution of 2-(trimethylsilyl)phenyl trifluoromethanesulfonate (**1a**) (88.3 mg, 0.297 mmol) and 1,1,3,3-tetramethylthiourea (**2f**) (38.1 mg, 0.288 mmol, 1.9 equiv) in THF (1.8 mL) in a 5 mL screw-top V-vial® with a solid-top cap (Sigma-Aldrich, Cat. No. Z115118) were added Cs_2CO_3 (205 mg, 0.630 mmol, 2.1 equiv) and 18-crown-6 (160 mg, 0.600 mmol, 2.0 equiv) at room temperature. After stirring for 24 h at room temperature, the mixture was filtered through a pad of Celite. The filtrate was concentrated under reduced pressure. To the residue was added 1,1,2,2-tetrachloroethane (15.6 mg, 93.0 μmol) as an internal standard, dissolved in $CDCl_3$, and ^1H NMR analysis (400 MHz) was performed. As a result, ^1H NMR yield of **3a** was determined to be 76%.

Similarly, thioxanthones **3d**, **3l**, and **3k** were prepared from the corresponding *o*-silylaryl triflates without aqueous workup and purification with silica-gel.

Gram scale synthesis of thioxanthone (3a)

To a solution of 2-(trimethylsilyl)phenyl trifluoromethanesulfonate (**1a**) (4.48 g, 15.0 mmol) and 1,1,3,3-tetramethylthiourea (**2f**) (1.99 g, 15.0 mmol, 2.0 equiv) in THF (90 mL) are added Cs_2CO_3 (10.3 g, 31.6 mmol, 2.1 equiv) and 18-crown-6 (7.92 g, 30.0 mmol, 2.0 equiv) at room temperature. After stirring for 24 h the same temperature, the mixture was filtered through a pad of Celite (320 g). The filtrate was concentrated under reduced pressure. The residual solid was washed with methanol (30 mL × 2) and dried under reduced pressure to give 9*H*-thioxanthen-9-one (**3a**) (1.27 g, 5.98 mmol, 80%) as a colorless solid.

Synthesis of thioxanthones (3a) through the treatment of various solid sorbents without aqueous workup

To a solution of 2-(trimethylsilyl)phenyl trifluoromethanesulfonate (**1a**) (88.8 mg, 0.298 mmol) and 1,1,3,3-tetramethylthiourea (**2f**) (39.9 mg, 0.302 mmol, 2.0 equiv) in THF (1.8 mL) in a 5 mL screw-top V-vial® with a solid-top cap (Sigma-Aldrich, Cat. No. Z115118) were added Cs_2CO_3 (206 mg, 0.632 mmol, 2.1 equiv) and 18-crown-6 (158 mg, 0.598 mmol, 2.0 equiv) at room temperature. After stirring for 24 h at room temperature, the mixture was filtered through a short pad of silica gel (6.5 g) with 35 φ elution tube, and the residue was washed with CH_2Cl_2. After filtration, the filtrate was concentrated under reduced pressure. The residue was purified by preparative TLC (*n*-hexane/AcOEt = 4/1) to give 9*H*-thioxanthen-9-one (**3a**) (23.4 mg, 0.110 mmol, 74%) as a colorless solid. When using florisil or Al_2O_3 as a sorbent instead of silica gel, **3a** was obtained in 75% or 77% yield, respectively. The efficient formation of **3a** was observed before the purification with preparative TLC. In contrast, **3a** was synthesized in only 13% yield when using amino-functionalized silica gel. These results clearly indicate that the carbonyl oxygen in **3a** would be introduced in the filtration with sorbents.

A typical procedure for the synthesis of thioxanthones without aqueous workup and purification with silica-gel

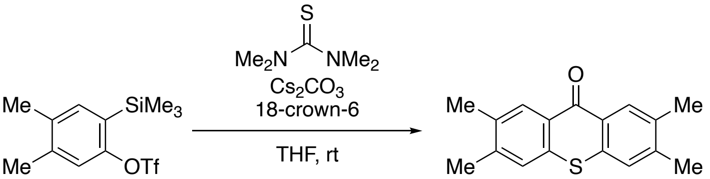

To a solution of 4,5-dimethyl-2-(trimethylsilyl)phenyl trifluoromethanesulfonate (**1b**) (92.0 mg, 0.282 mmol) and 1,1,3,3-tetramethylthiourea (**2f**) (39.3 mg, 0.297 mmol, 2.1 equiv) in THF (1.8 mL) were added Cs_2CO_3 (200 mg, 0.614 mmol, 2.2 equiv) and 18-crown-6 (158 mg, 0.598 mmol, 2.1 equiv) at room temperature. After stirring for 24 h at the same temperature, the mixture was filtered through a pad of Celite. The filtrate was concentrated under reduced pressure. The residual solid was washed with methanol (5 mL) and dried under reduced pressure to give 2,3,6,7-tetramethyl-9*H*-thioxanthen-9-one (**3b**) (24.5 mg, 91.3 µmol, 65%) as a colorless solid.

Similarly, thioxanthones **3b**, **3c**, and **3m**–**3o** were prepared from the corresponding *o*-silylaryl triflates without aqueous workup and purification with silica-gel.

*Synthesis of thioxanthone **3c** in 5 mmol scale*

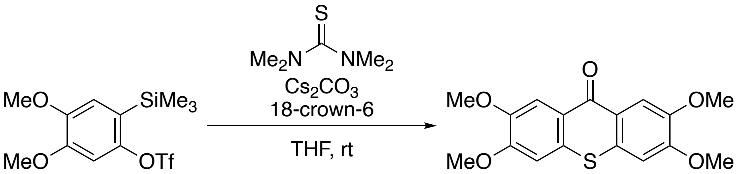

To a solution of 4,5-dimethoxy-2-(trimethylsilyl)phenyl trifluoromethanesulfonate (**1c**) (1.79 g, 4.99 mmol) and 1,1,3,3-tetramethylthiourea (**2f**) (663 mg, 5.01 mmol, 2.0 equiv) in THF (30 mL) were added Cs_2CO_3 (3.43 g, 10.5 mmol, 2.1 equiv) and 18-crown-6 (2.65 g, 10.0 mmol, 2.0 equiv) at room temperature. After stirring for 24 h at the same temperature, the mixture was filtered through a pad of Celite. The filtrate was concentrated under reduced pressure. The residual solid was washed with methanol (20 mL) and dried under reduced pressure to give 2,3,6,7-tetramethoxy-9*H*-thioxanthen-9-one (**3c**) (441 mg, 1.33 mmol, 53%) as a gray solid.

A typical procedure for the synthesis of thioxanthones through the hydrolysis with aq. NH_4Cl at 60 °C

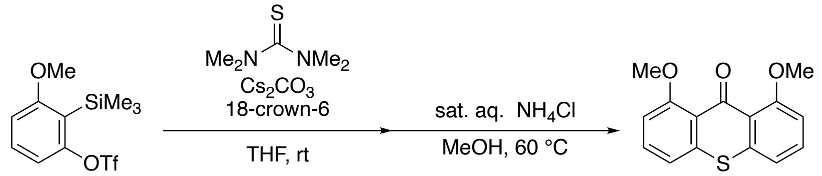

To a solution of 3-methoxy-2-(trimethylsilyl)phenyl trifluoromethanesulfonate (**1e**) (97.2 mg, 0.296 mmol) and 1,1,3,3-tetramethylthiourea (**2f**) (40.0 mg, 0.303 mmol, 2.0 equiv) in THF (1.8 mL) in a 5 mL screw-top V-vial® with a solid-top cap (Sigma-Aldrich, Cat. No. Z115118) were added Cs_2CO_3 (207 mg, 0.635 mmol, 2.2 equiv) and 18-crown-6 (160 mg, 0.606 mmol, 2.1 equiv) at room temperature. After stirring for 24 h at the same temperature, an aqueous saturated solution of ammonium chloride (2.0 mL) and methanol (1.0 mL) were added to the mixture at room temperature. After stirring for 24 h at 60 °C (with oil bath; bath temperature: 60 °C), to the mixture was added water (1 mL + 10 mL). The mixture was extracted with dichloromethane (15 mL × 3). The combined organic extract was washed with brine (20 mL) and dried with Na_2SO_4. After filtration, the filtrate was concentrated under reduced pressure. The residue was purified by preparative TLC (CH_2Cl_2/acetone = 50/1) to give 1,8-dimethoxy-9*H*-thioxanthen-9-one (**3e**) (26.9 mg, 98.8 µmol, 67%) as a colorless solid.

Similarly, thioxanthones **3e**–**3g** and **3i** and iminium salt **4a** were prepared from the corresponding *o*-silylaryl triflates and thioureas.

*A typical procedure for the synthesis of thioxanthone **3h** through the hydrolysis with aq. 1M HCl at 100 °C*

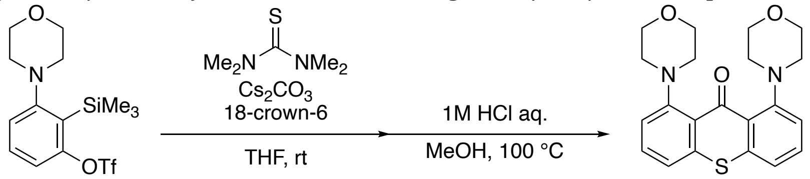

To a solution of 3-morpholino-2-(trimethylsilyl)phenyl trifluoromethanesulfonate (**1h**) (74.0 mg, 0.193 mmol) and 1,1,3,3-tetramethylthiourea (**2f**) (25.5 mg, 0.192 mmol, 2.0 equiv) in THF (1.2 mL) in a 5 mL screw-top V-vial® with a solid-top cap (Sigma-Aldrich, Cat. No. Z115118) were added Cs_2CO_3 (132 mg, 0.405 mmol, 2.1 equiv) and 18-crown-6 (104 mg, 0.393 mmol, 2.0 equiv) at room temperature. After stirring for 24 h at the same

temperature, aq. HCl (1.0 M, 1.4 mL) and methanol (0.7 mL) were added to the mixture at room temperature. After stirring for 24 h at 100 °C (with oil bath; bath temperature: 100 °C), to the mixture was added water (1 mL + 10 mL). The mixture was extracted with dichloromethane (15 mL × 3). The combined organic extract was washed with brine (20 mL) and dried with Na_2SO_4. After filtration, the filtrate was concentrated under reduced pressure. The residual solid was washed with methanol (5 mL) and dried under reduced pressure to give 1,8-dimorpholino-9*H*-thioxanthen-9-one (**3h**) (22.0 mg, 57.5 μmol, 60%) as a yellow solid. The filtrate was concentrated under reduced pressure. The residue was purified by preparative TLC (*n*-hexane/EtOAc = 4/1) to give1,8-dimorpholino-9*H*-thioxanthen-9-one (**3h**) (5.0 mg, 13 μmol, 14%) as a yellow solid.

A typical procedure for the synthesis of unsymmetric thioxanthones ***5***

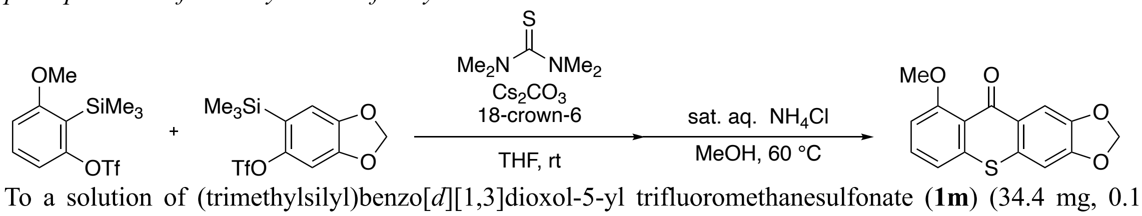

To a solution of (trimethylsilyl)benzo[*d*][1,3]dioxol-5-yl trifluoromethanesulfonate (**1m**) (34.4 mg, 0.100 mmol), 3-methoxy-2-(trimethylsilyl)phenyl trifluoromethanesulfonate (**1e**) (99.3 mg, 0.302 mmol, 3.0 equiv), and 1,1,3,3-tetramethylthiourea (52.9 mg, 0.400 mmol, 4.0 equiv) in THF (2.4 mL) were added Cs_2CO_3 (275 mg, 0.844 mmol, 2.1 equiv) and 18-crown-6 (211 mg, 0.799 mmol, 2.0 equiv) at room temperature. After stirring for 24 h at the same temperature, an aqueous saturated solution of ammonium chloride (2.7 mL) and MeOH (1.4 mL) were added to the mixture at room temperature. After stirring for 24 h at 60 °C (with oil bath; bath temperature: 60 °C), to the mixture was added water (10 mL). The mixture was extracted with dichloromethane (15 mL × 3). The combined organic extract was washed with brine (20 mL) and dried with Na_2SO_4. After filtration, the filtrate was concentrated under reduced pressure. The residue was filtered through a short pad of silica gel, which was washed with CH_2Cl_2 (10 mL). Concentration of the mixture in vacuo was purified by preparative TLC (CH_2Cl_2/acetone = 50/1) to give 9-methoxy-10*H*-thioxantheno[2,3-*d*][1,3]dioxol-10-one (**5a**) (12.9 mg, 45.1 μmol, 45%) as a colorless solid.

Similarly, unsymmetric thioxanthone **5b** was prepared from the corresponding *o*-silylaryl triflates **1b** and **1i**.

Synthesis of unsymmetric thioxanthone ***5c***

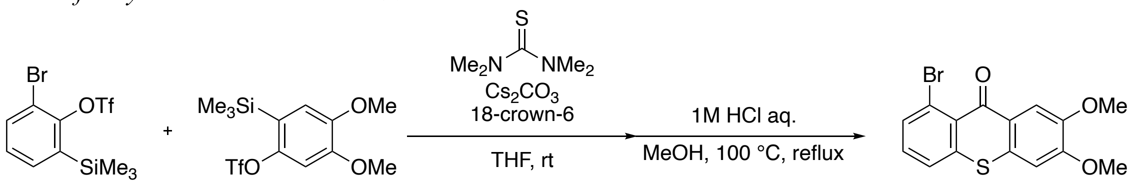

To a solution of 4,5-dimethoxy-2-(trimethylsilyl)phenyl trifluoromethanesulfonate (**1c**) (35.8 mg, 0.100 mmol), 2-bromo-6-(trimethylsilyl)phenyl trifluoromethanesulfonate (**1j**) (113 mg, 0.300 mmol, 3.0 equiv), and 1,1,3,3-tetramethylthiourea (**2f**) (52.9 mg, 0.400 mmol, 4.0 equiv) in THF (2.4 mL) were added Cs_2CO_3 (274 mg, 0.841 mmol, 2.1 equiv) and 18-crown-6 (211 mg, 0.799 mmol, 2.0 equiv) at room temperature. After stirring for 24 h at the same temperature, an aqueous HCl (1.0 M, 2.7 mL) and methanol (1.4 mL) were added to the mixture at room temperature. After stirring at 100 °C (with oil bath; bath temperature: 100 °C) for 24 h, to the mixture was added water (10 mL). The mixture was extracted with dichloromethane (15 mL × 3). The combined organic extract was washed with brine (20 mL) and dried with Na_2SO_4. After filtration, the filtrate was concentrated under reduced pressure. The residue was filtered through a short pad of silica gel, which was washed with CH_2Cl_2 (10 mL). Concentration of the mixture in vacuo was purified by preparative TLC (CH_2Cl_2/*n*-hexane = 2/1) to give 1-bromo-6,7-dimethoxy-9*H*-thioxanthen-9-one (**5c**) (13.1 mg, 37.3 μmol, 37%) as a brown solid.

Synthesis of 1,8-dimethoxy-9H-thioxanthen-9-one (3e) via the isolation of iminium salt ***4b***

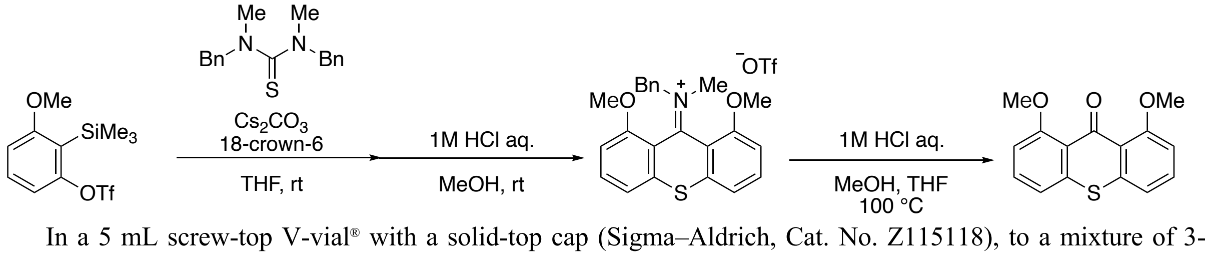

In a 5 mL screw-top V-vial® with a solid-top cap (Sigma–Aldrich, Cat. No. Z115118), to a mixture of 3-methoxy-2-(trimethylsilyl)phenyl trifluoromethanesulfonate (**1e**) (99.9 mg, 0.304 mmol) and 1,3-dibenzyl-1,3-dimethylthiourea (**2i**) (84.1 mg, 0.296 mmol, 1.9 equiv) dissolved in THF (1.8 mL) were added 18-crown-6 (159

mg, 0.602 mmol, 2.0 equiv) and Cs_2CO_3 (212 mg, 0.651 mmol, 2.1 equiv) at room temperature. After stirring for 24 h at the same temperature, an aqueous HCl (1.0 M, 2.0 mL) and methanol (1.3 mL) were added to the mixture at room temperature. After stirring for 24 h at the same temperature, the mixture was extracted with EtOAc (15 mL × 3). The combined organic extract was washed with brine (20 mL) and dried with Na_2SO_4. After filtration, the filtrate was concentrated under reduced pressure. The residue was purified by preparative TLC (CH_2Cl_2/methanol = 30/1) to give *N*-(1,8-dimethoxy-9*H*-thioxanthen-9-ylidene)-*N*-methyl-1-phenylmethanaminium trifluoromethanesulfonate (**4b**) (43.8 mg, 83.3 μmol, 55 %) as a yellow solid.

In a 5 mL screw-top V-vial® with a solid-top cap (Sigma–Aldrich, Cat. No. Z115118), to a mixture of *N*-(1,8-dimethoxy-9*H*-thioxanthen-9-ylidene)-*N*-methyl-1-phenylmethanaminium trifluoromethanesulfonate (**4b**) (19.1 mg, 36.3 μmol) dissolved in THF (0.44 mL) and MeOH (1.0 mL) was added an aqueous saturated HCl (1.0 M, 1.0 mL) at room temperature. After stirring at 100 °C (with oil bath; bath temperature: 100 °C) for 24 h, to the mixture was added water (1 mL). The mixture was extracted with dichloromethane (15 mL × 3). The combined organic extract was washed with brine (10 mL) and dried with Na_2SO_4. After filtration, the filtrate was concentrated under reduced pressure. The residue was purified by preparative TLC (CH_2Cl_2/acetone = 50/1) to give 1,8-dimethoxy-9*H*-thioxanthen-9-one (**3e**) (9.5 mg, 35 μmol, 96%) as a colorless solid.

Synthesis of N-(1,8-dimethoxy-9H-thioxanthen-9-ylidene)-N-methylmethanaminium trifluoromethanesulfonate (4c)

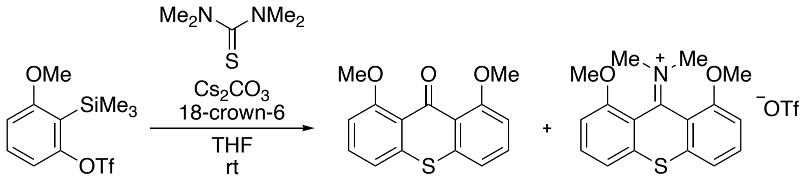

In a 5 mL screw-top V-vial® with a solid-top cap (Sigma–Aldrich, Cat. No. Z115118), to a mixture of 3-methoxy-2-(trimethylsilyl)phenyl trifluoromethanesulfonate (**1e**) (99.5 mg, 0.303 mmol) and 1,1,3,3-tetramethylthiourea (**2f**) (40.0 mg, 0.303 mmol, 2.0 equiv) dissolved in THF (1.8 mL) were added 18-crown-6 (160 mg, 0.606 mmol, 2.0 equiv) and Cs_2CO_3 (205 mg, 0.629 mmol, 2.1 equiv) at room temperature. After stirring for 24 h at the same temperature, to the mixture was added water (1 mL + 10 mL). The mixture was extracted with dichloromethane (15 mL × 3). The combined organic extract was washed with brine (5 mL) and dried with Na_2SO_4. After filtration, the filtrate was concentrated under reduced pressure. The residue was purified by column chromatography (silica-gel 10 g, CH_2Cl_2/MeOH = 10/1) to give 1,8-dimethoxy-9*H*-thioxanthen-9-one (**3e**) (1.3 mg, 4.8 μmol, 3%) as a colorless solid and an inseparable mixture of *N*-(1,8-dimethoxy-9*H*-thioxanthen-9-ylidene)-*N*-methylmethanaminium trifluoromethanesulfonate (**4c**, 83 μmol, 55%) and 18-crown-6 (37 μmol) as a yellow solid (47.2 mg; **4c**:18-crwon-6 = 69:31 (judged from ^{1}H NMR analysis)). To obtain an authentic sample of **4c**, further purification was carried out by recycling preparative HPLC system (SHIMADZU) equipped with a refractive index detector and a YMC-GPC T2000 (600 mm × 20 φ) column (YMC Co., Ltd.) using $CHCl_3$ as an eluent.

Synthesis of 1,8-dimethoxy-9H-thioxanthen-9-one (3e) via removal of cesium salts and the hydrolysis with aq. NH_4Cl at 60 °C

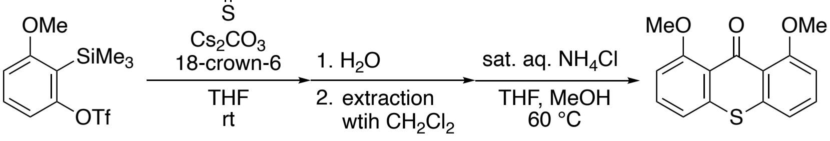

In a 5 mL screw-top V-vial® with a solid-top cap (Sigma–Aldrich, Cat. No. Z115118), to a mixture of 3-methoxy-2-(trimethylsilyl)phenyl trifluoromethanesulfonate (**1e**) (98.6 mg, 0.300 mmol) and 1,1,3,3-tetramethylthiourea (**2f**) (40.0 mg, 0.303 mmol, 2.0 equiv) dissolved in THF (1.8 mL) were added 18-crown-6 (160 mg, 0.605 mmol, 2.0 equiv) and Cs_2CO_3 (205 mg, 0.629 mmol, 2.1 equiv) at room temperature. After stirring for 24 h at the same temperature, to the mixture was added water (1 mL + 10 mL). The mixture was extracted with dichloromethane (15 mL × 3). The combined organic extract was washed with brine (10 mL) and dried with Na_2SO_4. After filtration, the filtrate was concentrated under reduced pressure. To the residue dissolved in THF (1.8 mL) and MeOH (1.0 mL) in a 5 mL screw-top V-vial® with a solid-top cap was added an aqueous saturated solution of ammonium chloride (2.0 mL) at room temperature. After stirring at 60 °C (with oil bath; bath temperature: 60 °C) for 24 h, to the mixture was added water (1 mL + 10 mL). The mixture was extracted with EtOAc (15 mL × 3). The combined organic extract was washed with brine (20 mL) and dried with Na_2SO_4. After filtration, the filtrate was concentrated under reduced pressure. The residue was purified by preparative TLC

(CH_2Cl_2/acetone = 50/1) to give 1,8-dimethoxy-9*H*-thioxanthen-9-one (**3e**) (10.6 mg, 38.9 μmol, 26%) as a colorless solid. This experiment was performed six times, in which **3e** was obtained in varied yields (12–41%).

Synthesis of 1,8-dimethoxy-9H-thioxanthen-9-one (3e) via removal of cesium salts and the hydrolysis with aq. NH_4Cl at 60 °C in the presence of CsOTf

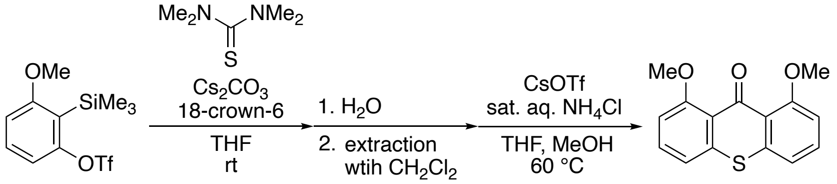

In a 5 mL screw-top V-vial® with a solid-top cap (Sigma–Aldrich, Cat. No. Z115118), to a mixture of 3-methoxy-2-(trimethylsilyl)phenyl trifluoromethanesulfonate (**1e**) (98.6 mg, 0.300 mmol) and 1,1,3,3-tetramethylthiourea (**2f**) (40.1 mg, 0.303 mmol, 2.0 equiv) dissolved in THF (1.8 mL) were added 18-crown-6 (160 mg, 0.606 mmol, 2.0 equiv) and Cs_2CO_3 (204 mg, 0.626 mmol, 2.1 equiv) at room temperature. After stirring for 24 h at the same temperature, to the mixture was added water (1 mL + 10 mL). The mixture was extracted with dichloromethane (15 mL × 3). The combined organic extract was washed with brine (20 mL) and dried with Na_2SO_4. After filtration, the filtrate was concentrated under reduced pressure. To the residue dissolved in THF (1.8 mL) and MeOH (1.0 mL) in a 5 mL screw-top V-vial® with a solid-top cap were added cesium trifluoromethanesulfonate (179 mg, 0.635 mmol, 2.1 equiv) and an aqueous saturated solution of ammonium chloride (2.0 mL) at room temperature. After stirring at 60 °C (with oil bath; bath temperature: 60 °C) for 24 h, to the mixture was added water (1 mL + 10 mL) at room temperature. The mixture was extracted with dichloromethane (15 mL × 3). The combined organic extract was washed with brine (20 mL) and dried with Na_2SO_4. After filtration, the filtrate was concentrated under reduced pressure. The residue was purified by preparative TLC (CH_2Cl_2/acetone = 50/1) to give 1,8-dimethoxy-9*H*-thioxanthen-9-one (**3e**) (27.2 mg, 99.9 μmol, 67%) as a colorless solid.

Synthesis of 2,3,6,7-tetramethoxy-9H-thioxanthen-9-one 10-oxide (7)

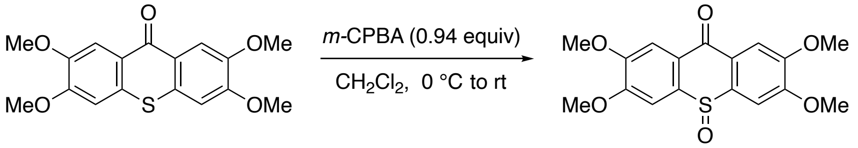

To a mixture of 2,3,6,7-tetramethoxy-9*H*-thioxanthen-9-one (**3c**) (69.1 mg, 0.208 mmol) in dichloromethane (2.9 mL) was added *m*-chloroperbenzoic acid (*m*-CPBA) (43.7 mg, 77%, 0.19 mmol, 0.94 equiv) at 0 ºC. After stirring at room temperature for 24 h, to the mixture was added an aqueous saturated solution of sodium bicarbonate (10 mL). The mixture was extracted with dichloromethane (15 mL × 3). The combined organic extract was washed with brine (5 mL). The extract was dried with Na_2SO_4. After filtration, the filtrate was concentrated under reduced pressure. The residue was purified by preparative TLC (CH_2Cl_2/acetone = 30/1) to give 2,3,6,7-tetramethoxy-9*H*-thioxanthen-9-one 10-oxide (**7**) (49.1 mg, 0.141 mmol, 68%) as a colorless solid.

*Synthesis of 2,3,6,7-tetramethoxy-9H-thioxanthen-9-one 10,10-dioxide (**8**)*

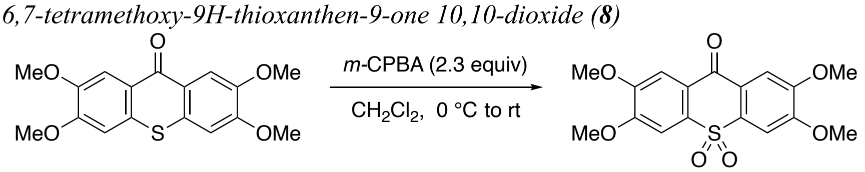

To a mixture of 2,3,6,7-tetramethoxy-9*H*-thioxanthen-9-one (**3c**) (45.6 mg, 0.137 mmol) in dichloromethane (0.60 mL) was added *m*-chloroperbenzoic acid (*m*-CPBA) (69.4 mg, 77%, 0.31 mmol, 2.3 equiv) at 0 ºC. After stirring at room temperature for 24 h, to the mixture was added an aqueous saturated solution of sodium bicarbonate (20 mL). The mixture was extracted with dichloromethane (15 mL × 3). The combined organic extract was washed with brine (5 mL). The extract was dried with Na_2SO_4. After filtration, the filtrate was concentrated under reduced pressure. The residual solid was washed with MeOH (5 mL) and dried under reduced pressure to give 2,3,6,7-tetramethoxy-9*H*-thioxanthen-9-one 10,10-dioxide (**8**) (44.8 mg, 0.123 mmol, 90%) as a yellow solid.

*Synthesis of 2,3,6,7-tetramethoxy-9-(4-methoxyphenyl)thioxanthylium trifluoromethanesulfonate (**10**)*

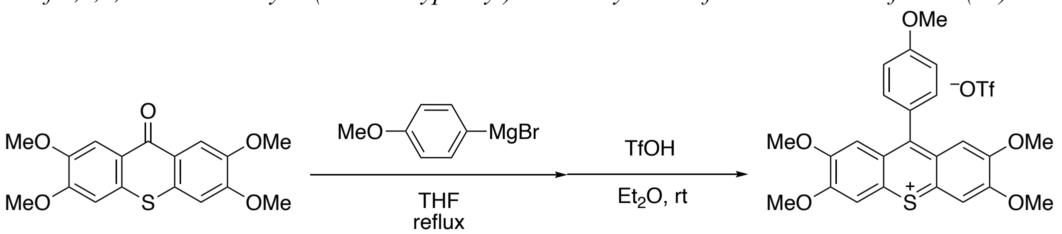

To a mixture of 2,3,6,7-tetramethoxy-9*H*-thioxanthen-9-one (**3c**) (34.8 mg, 0.105 mmol) in THF (1.0 mL) was added (4-methoxyphenyl)magnesium bromide (0.520 mL, 0.96 M in THF, 0.50 mmol, 4.8 equiv) dropwise at room temperature. After stirring at reflux (with oil bath; bath temperature: 80 ºC) for 24 h, to the mixture was added an aqueous saturated solution of ammonium chloride (20 mL) at room temperature. The mixture was extracted with EtOAc (20 mL × 3). The combined organic extract was washed with brine (10 mL) and dried with Na_2SO_4. After filtration, the filtrate was concentrated under reduced pressure, affording a crude product, which was used without further purification. To a solution of the obtained crude material in Et_2O (5.0 mL) was added trifluoromethanesulfonic acid (TfOH) (18.0 µL, 0.204 mmol, 1.9 equiv) dropwise at room temperature. The resulting mixture was filtrated, washed with Et_2O (20 mL), and dried under reduced pressure to afford 2,3,6,7-tetramethoxy-9-(4-methoxyphenyl)thioxanthylium trifluoromethanesulfonate (**10**) (46.3 mg, 80.9 µmol, 77%) as a red solid.

*Synthesis of 4-(trimethylsilyl)dibenzo[b,d]furan-3-yl trifluoromethanesulfonate (**1o**)*

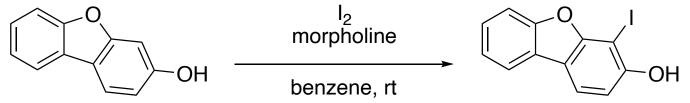

To a solution of iodine (2.25 g, 8.86 mmol, 1.5 equiv) dissolved in benzene (24 mL) was added morpholine (2.10 mL, 24.4 mmol, 4.0 equiv) at room temperature. After stirring for 20 min at the same temperature, to the mixture was added dibenzo[*b*,*d*]furan-3-ol (**6**) (1.11 g, 6.03 mmol) at room temperature. After stirring for 1 h at the same temperature, to the mixture was added an aqueous sodium thiosulfate (20 mL). The mixture was extracted with EtOAc (20 mL × 3). The combined organic extract was washed with brine (10 mL), and then dried with Na_2SO_4. After filtration, the filtrate was concentrated under reduced pressure. The resulting mixture was purified by column chromatography (silica-gel 43 g, CH_2Cl_2) to give 4-iododibenzo[*b*,*d*]furan-3-ol (806 mg, 2.60 mmol, 43%) as a colorless solid.

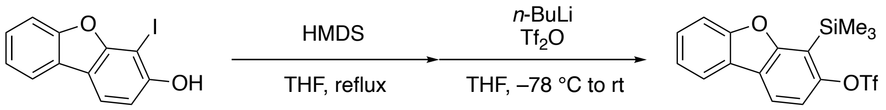

A mixture of 4-iododibenzo[*b*,*d*]furan-3-ol (806 mg, 2.60 mmol) in THF (9.0 mL) was added 1,1,1,3,3,3-hexamethyldisilazane (HMDS) (0.700 mL, 3.33 mmol, 1.3 equiv) at room temperature. After stirring at reflux (with oil bath; bath temperature: 80 °C) for 10 h, volatile substances were removed under reduced pressure, affording a crude product, which was used without further purification. To a solution of the obtained crude material in THF (6.2 mL) was added *n*-BuLi (1.20 mL, 2.7 M in *n*-hexane, 3.2 mmol, 1.2 equiv) dropwise at –78 °C. After stirring for 30 min at the same temperature, to the mixture was added trifluoromethanesulfonic anhydride (0.520 mL, 3.17 mmol, 1.2 equiv) in a dropwise manner at –78 °C. After stirring for 30 min at the same temperature, the mixture was allowed to warm to room temperature. After stirring for 20 min at the same temperature, to the mixture was added an aqueous saturated sodium hydrogen carbonate solution (10 mL). The mixture was extracted with EtOAc (20 mL × 3). The combined organic extract was washed with brine (10 mL) and dried with Na_2SO_4. After filtration, the filtrate was concentrated under reduced pressure. The residue was purified by column chromatography (silica-gel 90 g, *n*-hexane/CH_2Cl_2 = 2/1) to give 4-(trimethylsilyl)dibenzo[*b*,*d*]furan-3-yl trifluoromethanesulfonate (**1o**) (606 mg, 1.56 mmol, 60%) as a yellow solid.

*Synthesis of 1,3-dibenzyl-1,3-dimethylthiourea (**2i**)*

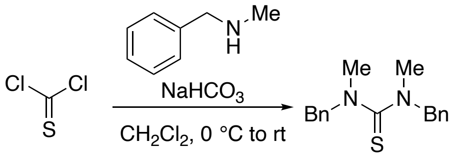

To a mixture of *N*-methyl-1-phenylmethanamine (390 µL, 367 mg, 3.03 mmol, 3.0 equiv) in dichloromethane (14 mL) was added sodium bicarbonate (571 mg, 6.80 mmol, 6.8 equiv) at 0 °C. After stirring for 5 min at the

same temperature, to the mixture was added thiophosgene (76.1 μL, 114 mg, 0.993 mmol) at 0 °C in a dropwise manner. The mixture was stirred for another 30 min at the same temperature. Then, the mixture was allowed to warm to room temperature and stirred for 18 h at room temperature. To the mixture was added water (10 mL). The mixture was extracted with dichloromethane (15 mL × 3). The combined organic extract was washed with brine (5 mL). The extract was dried with Na_2SO_4. After filtration, the filtrate was concentrated under reduced pressure. The residue was purified by column chromatography (silica-gel 30 g, *n*-hexane/EtOAc = 1/1) to give 1,3-dibenzyl-1,3-dimethylthiourea (**2i**) (263 mg, 0.925 mmol, 93%) as a yellow solid.

Absorption and fluorescent properties and spectra

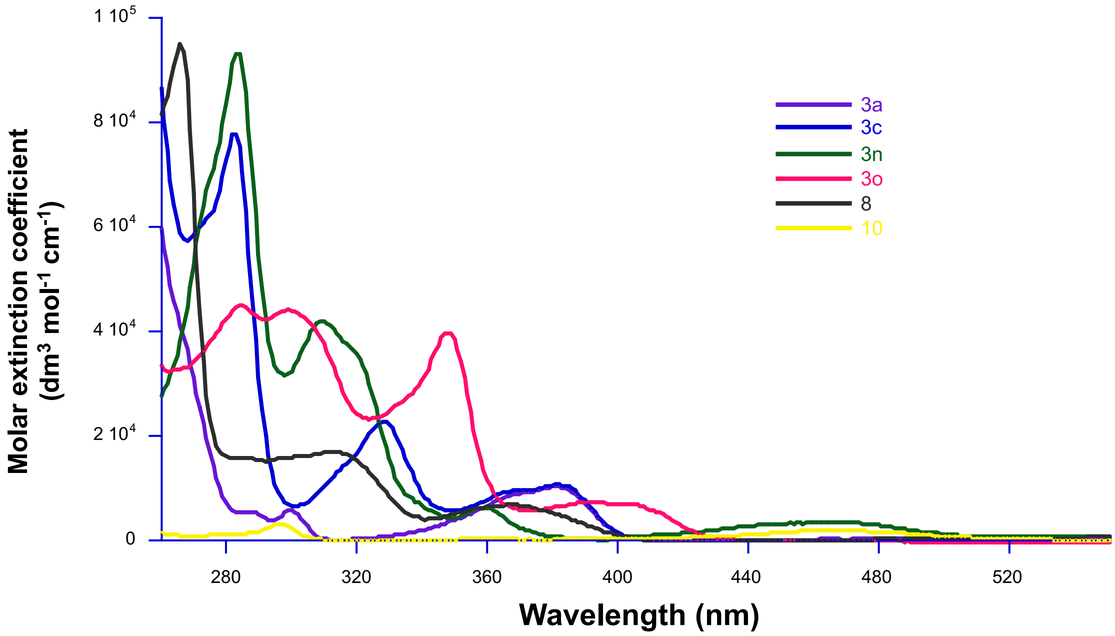

Compound	λ_{abs} [nm] (log ε)
**3a**	300 (3.75), 381 (4.01)
**3c**	328 (3.35), 382 (3.02)
**3n**	310 (4.62), 360 (3.79), 465(3.53)
**3o**	348 (4.65), 392 (3.92)
**8**	313 (4.23), 368 (3.83)
**10**	464 (3.25)

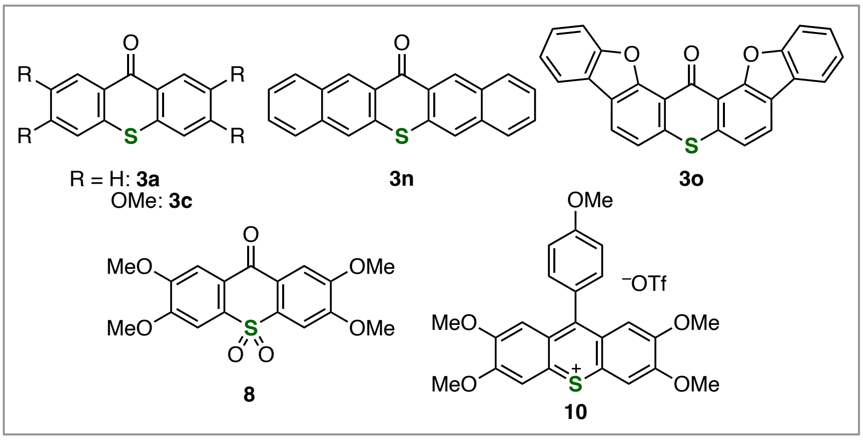

Fig. S1: UV-Vis spectra of **3a, 3c, 3n, 3o, 8,** and **10** in CH_2Cl_2 and summary table of observed representative absorption peaks **3a, 3c, 3m 3n, 8,** and **10**. Concentrations: **3a** = 3.0 × 10^{-6} M, **3c** = 1.9 × 10^{-6} M, **3n** = 7.0 × 10^{-7} M, **3o** = 9.7 × 10^{-7} M, **8** = 2.1 × 10^{-6} M, **10** = 1.0 × 10^{-6} M.

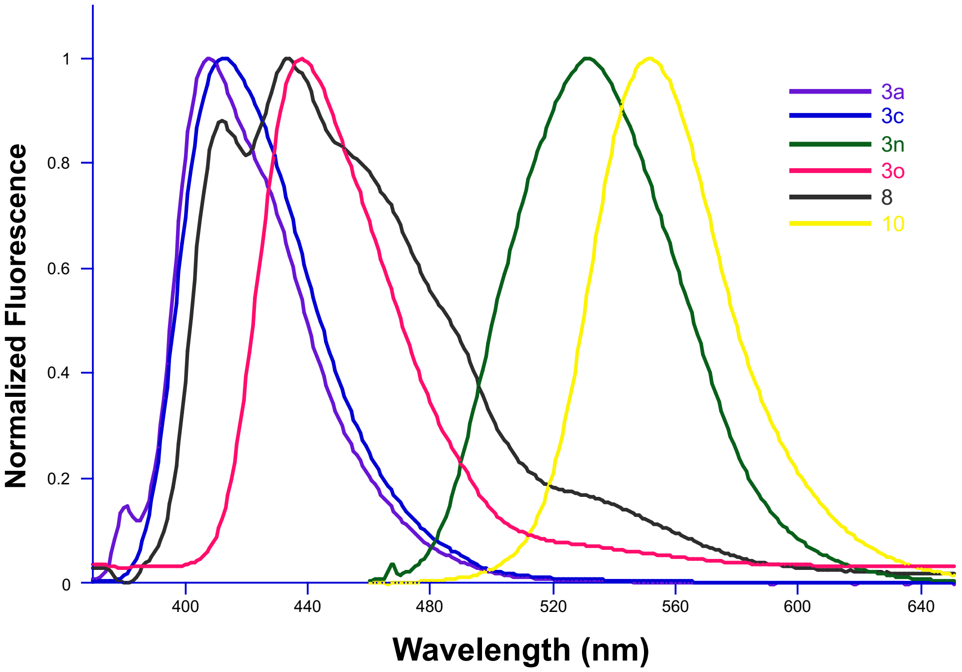

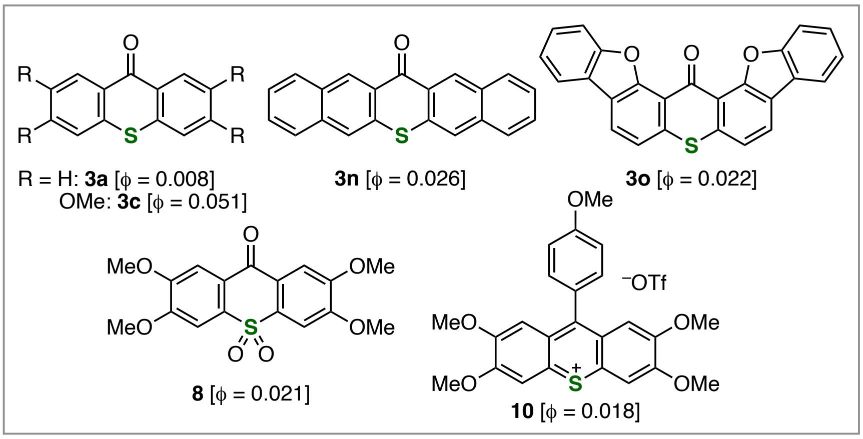

Fig. S2: Emission spectra of **3a, 3c, 3n, 3o, 8,** and **10** in CH_2Cl_2.
Excitation wavelengths for each compound: **3a** (408 nm), **3c** (414 nm), **3n** (531 nm), **3o** (438 nm), **8** (433 nm), and **10** (551 nm). Concentrations: **3a** = 3.0×10^{-6} M, **3c** = 1.9×10^{-6} M, **3n** = 1.3×10^{-6} M, **3o** = 9.7×10^{-7} M, **8** = 2.1×10^{-6} M, **10** = 1.0×10^{-6} M. The excitation wavelength was 350 nm (**3a, 3c, 3o, 8**) or 450 nm (**3n, 10**).

Characterization Data of New Compounds

9*H*-Thioxanthen-9-one (**3a**)[S12] and 1,8-dimethoxy-9*H*-thioxanthen-9-one (**3e**)[S13] were identical in spectra data with those reported in the literature.

2,3,6,7-Tetramethyl-9*H*-thioxanthen-9-one (**3b**)

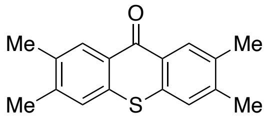

Yield: 65% (24.5 mg, 91.3 μmol); Colorless solid; Mp 278–280 °C; TLC R_f 0.60 (CH_2Cl_2/*n*-hexane = 2/1); ^1H NMR ($CDCl_3$, 400 MHz): δ 8.38 (s, 2H), 7.33 (s, 2H), 2.39 (s, 6H), 2.38 (s, 6H); $^{13}C\{^1H\}$ NMR ($CDCl_3$, 101 MHz): δ 180.1, 142.7, 135.7, 135.0, 130.3, 127.7, 126.7, 20.5, 19.9; IR (NaCl, cm^{-1}) 871, 911, 999, 1027, 1075, 1132, 1225, 1265, 1322, 1372, 1402, 1449, 1480, 1593, 1627; HRMS (ESI) *m/z*: $[M + Na]^+$ Calcd for $C_{17}H_{16}NaOS^+$ 291.0820; Found 291.0815.

2,3,6,7-Tetramethoxy-9*H*-thioxanthen-9-one (**3c**)

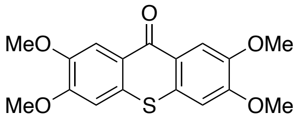

Yield: 61% (26.1 mg, 78.5 μmol); Gray solid; Mp 268–270 °C; TLC R_f 0.46 (CH_2Cl_2); ^1H NMR ($CDCl_3$, 400 MHz): δ 8.09 (s, 2H), 6.97 (s, 2H), 4.05 (s, 6H), 4.04 (s, 6H); $^{13}C\{^1H\}$ NMR ($CDCl_3$, 101 MHz): δ 177.6, 152.9, 148.6, 131.0, 122.7, 109.9, 106.4, 56.3, 56.2; IR (NaCl, cm^{-1}) 1056, 1222, 1253, 1268, 1409, 1441, 1455, 1462, 1504, 1591, 1644; HRMS (ESI) *m/z*: $[M + Na]^+$ Calcd for $C_{17}H_{16}NaO_5S^+$ 355.0616; Found 355.0617.

2,3,6,7-Tetrafluoro-9*H*-thioxanthen-9-one (**3d**)

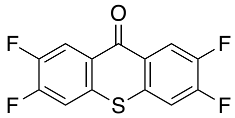

Yield: 25% (10.4 mg, 36.6 μmol); Colorless solid; Mp 219–220 °C; TLC R_f 0.63 (CH_2Cl_2/*n*-hexane = 2/1); ^1H NMR ($CDCl_3$, 400 MHz): δ 8.41 (dd, 2H, *J* = 10.8, 8.2 Hz), 7.40 (dd, 2H, *J* = 9.6, 6.7 Hz); $^{13}C\{^1H\}$ NMR ($CDCl_3$, 101 MHz): δ 176.6, 153.6 (dd, *J* = 261, 14.7 Hz), 149.8 (dd, *J* = 252, 13.3 Hz), 133.4 (dd, *J* = 7.7, 2.7 Hz), 125.5 (dd, *J* = 4.7, 2.8 Hz), 118.5 (dd, *J* = 18.8, 2.2 Hz), 114.2 (d, *J* = 20.6 Hz); $^{19}F\{^1H\}$ NMR ($CDCl_3$, 377 MHz): δ −135.9 (d, 2F, *J* = 21.3 Hz), −127.2 (d, 2F, *J* = 21.3 Hz); IR (NaCl, cm^{-1}) 857, 903, 1017, 1107, 1222, 1279, 1336, 1388, 1495, 1613, 1640; HRMS (ESI) *m/z*: $[M + H]^+$ Calcd for $C_{13}H_5F_4OS^+$ 284.9997; Found 284.9999.

3,6-Dibromo-1,8-dimethoxy-9*H*-thioxanthen-9-one (**3f**)

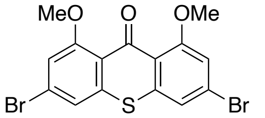

Yield: 28% (11.6 mg, 27.0 μmol); Colorless solid; Mp 239–240 °C; TLC R_f 0.52 (CH_2Cl_2/acetone = 30/1); ^1H NMR ($CDCl_3$, 400 MHz): δ 7.21 (d, 2H, *J* = 1.6 Hz), 7.03 (d, 2H, *J* = 1.6 Hz), 3.97 (s, 6H); $^{13}C\{^1H\}$ NMR ($CDCl_3$, 101 MHz): δ 180.7, 160.7, 138.5, 126.4, 121.7, 120.0, 113.5, 56.9; IR (NaCl, cm^{-1}) 823, 916, 1049, 1248, 1298, 1391, 1451, 1547, 1557, 1568, 1651; HRMS (ESI) *m/z*: $[M + Na]^+$ Calcd for $C_{15}H_{10}{}^{79}Br_2NaO_3S^+$ 450.8615; Found 450.8615.

1,8-Dimethoxy-3,6-diphenyl-9*H*-thioxanthen-9-one (**3g**)

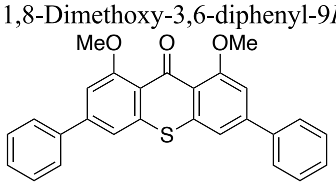

Yield: 35% (15.5 mg, 36.5 μmol); Colorless solid; Mp 197–198 ℃; TLC R_f 0.60 (CH_2Cl_2/acetone = 20/1); ^1H NMR ($CDCl_3$, 400 MHz): δ 7.66–7.60 (AA'BB'C, 4H), 7.51–7.40 (m, 6H), 7.28–7.23 (m, 2H), 7.08 (d, 2H, *J* = 1.3 Hz), 4.05 (s, 6H); $^{13}C\{^1H\}$ NMR ($CDCl_3$, 101 MHz): δ 181.6, 160.4, 144.9, 139.6, 137.9, 129.0, 128.5, 127.3, 121.6, 115.8, 108.4, 56.4; IR (NaCl, cm^{-1}) 846, 874, 927, 1047, 1235, 1265, 1321, 1391, 1454, 1501, 1538, 1551, 1594, 1657; HRMS (ESI) *m/z*: $[M + Na]^+$ Calcd for $C_{27}H_{20}NaO_3S^+$ 447.1031; Found 447.1038.

1,8-Dimorpholino-9*H*-thioxanthen-9-one (**3h**)

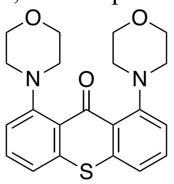

Yield: 73% (27.0 mg, 70.7 μmol); Yellow solid; Mp 247–249 °C; TLC R_f 0.45 (CH_2Cl_2/methanol = 30/1); ^{1}H NMR ($CDCl_3$, 400 MHz): δ 7.38 (dd, 2H, J = 8.0, 8.0 Hz), 7.14 (dd, 2H, J = 8.0, 0.8 Hz), 7.04 (dd, 2H, J = 8.0, 0.8 Hz), 4.01–3.89 (AA'BB', 8H), 3.24–3.12 (AA'BB', 8H); ^{13}C{^{1}H} NMR ($CDCl_3$, 101 MHz): δ 184.1, 153.2, 137.0, 131.2, 126.8, 119.7, 117.2, 67.4, 53.7; IR (NaCl, cm^{-1}) 962, 1066, 1113, 1237, 1253, 1429, 1442, 1452, 1555, 1571, 1658; HRMS (ESI) *m/z*: $[M + H]^+$ Calcd for $C_{21}H_{23}N_2O_3S^+$ 383.1429; Found 383.1426.

1,8-Bis(dimethylamino)-9*H*-thioxanthen-9-one (**3i**)

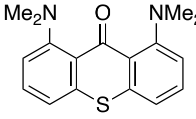

Yield: 69% (20.5 mg, 68.7 μmol); Yellow solid; Mp 127–130 °C; TLC R_f 0.63 (CH_2Cl_2/methanol = 15/1); ^{1}H NMR ($CDCl_3$, 400 MHz): δ 7.32 (dd, 2H, J = 8.0, 8.0 Hz), 7.03–6.96 (m, 4H), 2.94 (s, 12H); ^{13}C{^{1}H} NMR ($CDCl_3$, 101 MHz): δ 184.7, 153.7, 136.6, 130.6, 125.8, 117.2, 115.9, 45.4; IR (NaCl, cm^{-1}) 1062, 1096, 1125, 1130, 1198, 1209, 1232, 1253, 1361, 1366, 1404, 1418, 1448, 1552, 1568, 1633; HRMS (ESI) *m/z*: $[M + Na]^+$ Calcd for $C_{17}H_{18}N_2NaOS^+$ 321.1038; Found 321.1037.

3,6-Dimethyl-9*H*-thioxanthen-9-one (**3k**)

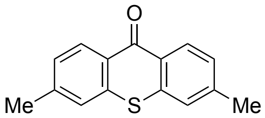

Yield: 27% (9.7 mg, 40 μmol); Colorless solid; Mp 122–123 °C; TLC R_f 0.50 (CH_2Cl_2/*n*-hexane = 2/1); ^{1}H NMR ($CDCl_3$, 400 MHz): δ 8.53 (d, 2H, J = 8.3 Hz), 7.38 (s, 2H), 7.34–7.26 (m, 2H), 2.49 (s, 6H); ^{13}C{^{1}H} NMR ($CDCl_3$, 101 MHz): δ 179.6, 143.1, 137.3, 129.8, 127.8, 127.1, 125.8, 21.7; IR (NaCl, cm^{-1}) 844, 931, 1128, 1169, 1316, 1395, 1451, 1558, 1601, 1631; HRMS (ESI) *m/z*: $[M + H]^+$ Calcd for $C_{15}H_{13}OS^+$ 241.0682; Found 241.0684.

2,6-Dimethyl-9*H*-thioxanthen-9-one (**3k'**)

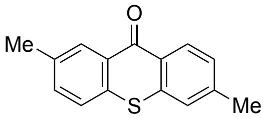

Yield: 42% (14.9 mg, 62.0 μmol); colorless solid; Mp 161–162 °C; TLC R_f 0.56 (CH_2Cl_2/*n*-hexane = 2/1); ^{1}H NMR ($CDCl_3$, 400 MHz): δ 8.53 (d, 1H, J = 8.2 Hz), 8.45 (s, 1H), 7.53–7.43 (m, 2H), 7.39 (s, 1H), 7.34–7.26 (m, 1H), 2.50 (m, 3H+3H); ^{13}C{^{1}H} NMR ($CDCl_3$, 101 MHz): δ 179.9, 143.2, 137.5, 136.3, 134.1, 133.6, 129.8, 129.6, 129.1,127.7, 127.1, 125.9, 125.8, 21.7, 21.3; IR (NaCl, cm^{-1}) 1126, 1295, 1319, 1457, 1507, 1558, 1603, 1634; HRMS (ESI) *m/z*: $[M + H]^+$ Calcd for $C_{15}H_{13}OS^+$ 241.0682; Found 241.0682.

2,7-Dimethyl-9*H*-thioxanthen-9-one (**3k"**)

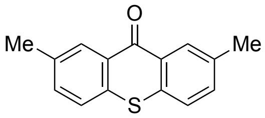

Yield: 22% (7.9 mg, 33 μmol); Colorless solid; Mp 175–177 °C; TLC R_f 0.65 (CH_2Cl_2/*n*-hexane = 2/1); ^{1}H NMR ($CDCl_3$, 400 MHz): δ 8.46 (d, 2H, J = 0.8 Hz), 7.54–7.44 (m, 4H), 2.51 (s, 6H); ^{13}C{^{1}H} NMR ($CDCl_3$, 101 MHz): δ 180.2, 136.2, 134.3, 133.6, 129.6, 129.0, 125.9, 21.2; IR (NaCl, cm^{-1}) 1079, 1126, 1182, 1218, 1285, 1319, 1408, 1471, 1604, 1640; HRMS (ESI) *m/z*: $[M + H]^+$ Calcd for $C_{15}H_{13}OS^+$ 241.0682; Found 241.0684.

2,3,8,9-Tetrahydro-1*H*-dicyclopenta[*b*,*i*]thioxanthen-11(7*H*)-one (**3l**)

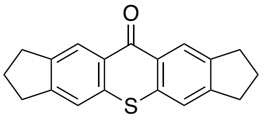

Yield: 46% (20.1 mg, 68.7 μmol); Yellow solid; Mp 226–228 °C; TLC R_f 0.46 (CH_2Cl_2/*n*-hexane = 2/1); ^{1}H NMR ($CDCl_3$, 400 MHz): δ 8.50 (s, 2H), 7.44 (s, 2H), 3.09–2.98 (m, 8H), 2.17 (tt, 4H, *J* = 7.5, 7.5 Hz); ^{13}C{^{1}H} NMR ($CDCl_3$, 101 MHz): δ 180.2, 149.9, 143.5, 135.4, 127.9, 125.0, 121.0, 33.0, 32.4, 25.6; IR (NaCl, cm^{-1}) 851, 896, 913, 1053, 1110, 1209, 1271, 1403, 1462, 1601, 1632; HRMS (ESI) *m/z*: [M + Na]$^+$ Calcd for $C_{19}H_{16}NaOS^+$ 315.0820; Found 315.0819.

11*H*-Thioxantheno[2,3-*d*:6,7-*d'*]bis([1,3]dioxole)-11-one (**3m**)

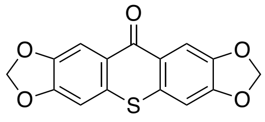

Yield: 57% (25.5 mg, 84.9 μmol); Yellow solid; Mp 286–287 °C; TLC R_f 0.32 (CH_2Cl_2); ^{1}H NMR ($CDCl_3$, 400 MHz): δ 8.04 (s, 2H), 6.96 (s, 2H), 6.13 (s, 4H); ^{13}C{^{1}H} NMR ($CDCl_3$, 101 MHz): δ 177.2, 151.5, 147.9, 132.6, 124.2, 107.8, 104.0, 102.2; IR (NaCl, cm^{-1}) 933, 1032, 1089, 1417, 1428, 1471, 1583, 1610; HRMS (ESI) *m/z*: [M + Na]$^+$ Calcd for $C_{15}H_8NaO_5S^+$ 322.9990; Found 322.9992.

13*H*-Dibenzo[*b*,*i*] thioxanthene-13-one (**3n**)

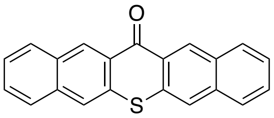

Due to its poor solubility (<1mg/1mL in $CDCl_3$), obtaining clean ^{1}H and ^{13}C spectra with a high signal-to-noise ratio was challenging for our instrument. ^{13}C NMR spectrum was obtained by the analysis for 13 h (cumulative numbers ca. 20000).
Yield: 50% (22.5 mg, 72.0 μmol); Yellow solid; Mp 347–348 °C; TLC R_f 0.58 (CH_2Cl_2/*n*-hexane = 2/1); ^{1}H NMR ($CDCl_3$, 400 MHz): δ 9.21 (s, 2H), 8.09 (d, 2H, *J* = 8.2 Hz), 8.03 (s, 2H), 7.86 (d, 2H, *J* = 8.2 Hz), 7.65 (ddd, 2H, *J* = 6.8, 6.8, 1.2 Hz), 7.54 (ddd, 2H, *J* = 6.8, 6.8, 1.2 Hz); ^{13}C{^{1}H} NMR ($CDCl_3$, 101 MHz): δ 181.9, 135.1, 132.0, 131.9, 131.0, 130.1, 129.5, 126.8, 126.5, 126.1, 123.6; IR (NaCl, cm^{-1}) 800, 881, 931, 959, 1072, 1180, 1319, 1428, 1644; HRMS (ESI) *m/z*: [M + Na]$^+$ Calcd for $C_{21}H_{12}NaOS^+$ 335.0507; Found 335.0508.

N-(1,8-Bis((4-methoxyphenyl)thio)-9*H*-thioxanthen-9-ylidene)-*N*-methylmethanaminium trifluoromethanesulfonate (**4a**)

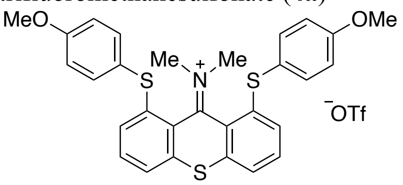

Yield: 47% (30.2 mg, 47.4 μmol); Yellow solid; Mp 68–69 °C; TLC R_f 0.54 (CH_2Cl_2/methanol = 15/1); ^{1}H NMR ($CDCl_3$, 400 MHz): δ 7.56–7.50 (m, 6H), 7.35 (dd, 2H, *J* = 8.1, 8.1 Hz), 7.06–7.01 (AA'BB', 4H), 6.93 (dd, 2H, *J* = 8.1, 0.8 Hz), 4.16 (s, 6H), 3.90 (s, 6H); ^{13}C{^{1}H} NMR ($CDCl_3$, 101 MHz): δ 174.0, 161.5, 140.4, 136.9, 136.0, 131.6, 126.2, 124.2, 123.3, 120.8 (q, *J* = 320 Hz), 118.5, 116.0, 55.6, 46.8; ^{19}F{^{1}H}NMR ($CDCl_3$, 377 MHz): δ −78.2 (s); IR (NaCl, cm^{-1}) 833, 1030, 1153, 1254, 1435, 1494, 1558, 1591, 1628, 2849, 2920, 3061; HRMS (ESI) *m/z*: [M − OTf]$^+$ Calcd for $C_{29}H_{26}NO_2S_3^+$ 516.1126; Found 516.1121.

9-Methoxy-10*H*-thioxantheno[2,3-*d*][1,3]dioxol-10-one (**5a**)

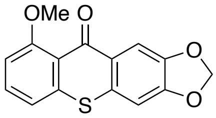

Yield: 45% (12.9 mg, 45.1 μmol); Colorless solid; Mp 182–185 °C; TLC R_f 0.39 (CH_2Cl_2/acetone = 30/1); ^{1}H NMR ($CDCl_3$, 400 MHz): δ 7.92 (s, 1H), 7.46 (dd, 1H, *J* = 8.2, 8.2 Hz), 7.10 (dd, 1H, *J* = 8.2, 0.6 Hz), 6.91 (dd, 1H, *J* = 8.2, 0.6 Hz), 6.85 (s, 1H), 6.07 (s, 2H), 4.01 (s, 3H); ^{13}C{^{1}H} NMR ($CDCl_3$, 101 MHz): δ 179.4, 162.4, 151.5, 148.0, 139.9, 132.6, 131.1,127.5, 119.3, 118.1, 109.1, 108.6, 103.8, 102.3, 56.6; IR (NaCl, cm^{-1}) 936, 1022, 1042, 1203, 1255, 1395, 1421, 1454, 1477, 1501, 1587, 1614; HRMS (ESI) *m/z*: [M + Na]$^+$ Calcd for

$C_{15}H_{10}NaO_4S^+$ 309.0198; Found 309.0197.

1-(Dimethylamino)-6,7-dimethyl-9*H*-thioxanthen-9-one (**5b**)

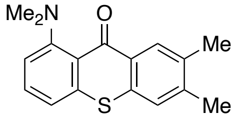

Yield: 46% (16.0 mg, 56.5 μmol); Yellow solid; Mp 136–137 °C; TLC R_f 0.44 (CH_2Cl_2/*n*-hexane = 2/1); ^{1}H NMR ($CDCl_3$, 400 MHz): δ 8.13 (s, 1H), 7.35 (dd, 1H, *J* = 8.2, 8.2 Hz), 7.25 (s, 1H), 6.91 (dd, 1H, *J* = 8.2, 0.9 Hz), 6.87 (dd, 1H, *J* = 8.2, 0.9 Hz), 2.98 (s, 6H), 2.38 (s, 3H), 2.37 (s, 3H); ^{13}C{^{1}H} NMR ($CDCl_3$, 101 MHz): δ 181.7, 154.4, 141.7, 139.5, 135.3, 132.9, 131.8, 131.1, 129.9, 125.6, 118.2, 115.1, 113.0, 44.1, 20.3, 19.9; IR (NaCl, cm^{-1}) 980, 1035, 1065, 1089, 1123, 1135, 1192, 1211, 1265, 1372, 1387, 1405, 1422, 1447, 1455, 1488, 1557, 1574, 1601; HRMS (ESI) *m/z*: [M + Na]$^+$ Calcd for $C_{17}H_{17}NNaOS^+$ 306.0929; Found 306.0929.

1-Bromo-6,7-dimethoxy-9*H*-thioxanthen-9-one (**5c**)

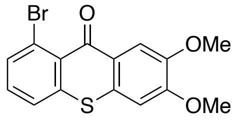

Yield: 37% (13.1 mg, 37.3 μmol); Brown solid; Mp 195–197 °C; TLC R_f 0.71 (CH_2Cl_2); ^{1}H NMR ($CDCl_3$, 400 MHz): δ 7.98 (s, 1H), 7.77 (dd, 1H, *J* = 7.9, 1.1 Hz), 7.55 (dd, 1H, *J* = 7.9, 1.1 Hz), 7.33 (dd, 1H, *J* = 7.9, 7.9 Hz), 6.87 (s, 1H), 4.02 (s, 3H + 3H); ^{13}C{^{1}H} NMR ($CDCl_3$, 101 MHz): δ 178.5, 153.3, 148.8, 139.7, 134.1, 131.1, 128.9, 126.1, 125.8, 124.3, 124.0, 110.5, 106.0, 56.4, 56.2; IR (NaCl, cm^{-1}) 1066, 1142, 1213, 1268, 1391, 1427, 1465, 1507, 1597, 1631; HRMS (ESI) *m/z*: [M + Na]$^+$ Calcd for $C_{15}H_{11}{}^{79}BrNaO_3S^+$ 372.9510; Found 372.9508.

N-(1,8-Dimethoxy-9*H*-thioxanthen-9-ylidene)-*N*-methyl-1-phenylmethanaminium trifluoromethanesulfonate (**4b**)

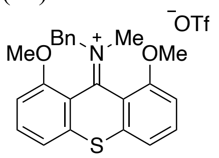

Yield: 55% (43.8 mg, 83.3 μmol); Yellow solid; Mp 141–142 °C ; TLC R_f 0.52 (CH_2Cl_2/methanol = 10/1); ^{1}H NMR ($CDCl_3$, 400 MHz): δ 7.59–7.52 (m, 2H), 7.39 (dd, 1H, *J* = 7.9, 0.6 Hz), 7.36–7.31 (m, 4H), 7.18–7.13 (m, 2H), 7.09–7.05 (m, 2H), 5.45 (d, 1H, *J* = 14.4 Hz), 5.27 (d, 1H, *J* = 14.4 Hz), 4.12 (s, 3H), 4.05 (s, 3H), 3.59 (s, 3H); ^{13}C{^{1}H} NMR ($CDCl_3$, 101 MHz): δ 169.8, 156.5, 155.7, 137.1, 136.9, 133.5, 133.3, 131.5, 130.5 (q, *J* = 269 Hz), 129.5, 128.5, 122.6, 119.8, 119.4, 116.8, 116.7, 110.3, 110.2, 61.5, 56.69, 56.67, 43.1; ^{19}F{^{1}H} NMR ($CDCl_3$, 377 MHz): δ −78.2 (s); IR (NaCl, cm^{-1}): 1030, 1155, 1225, 1275, 1435, 1471, 1498, 1584, 1621, 1722, 2849, 2952, 3069; HRMS (ESI) *m/z*: [M − OTf]$^+$ Calcd for $C_{23}H_{22}NO_2S^+$ 376.1371; Found 376.1365.

N-(1,8-Dimethoxy-9*H*-thioxanthen-9-ylidene)-*N*-methylmethanaminium trifluoromethanesulfonate (**4c**)

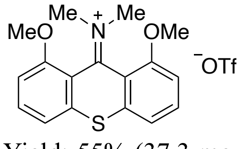

Yield: 55% (37.3 mg, 82.9 μmol); Yellow solid; Mp 178–179 °C ; TLC R_f 0.25 (CH_2Cl_2/methanol = 10/1); ^{1}H NMR ($CDCl_3$, 400 MHz): δ 7.55 (dd, 2H, *J* = 8.0, 8.0 Hz), 7.33 (dd, 2H, *J* = 8.0, 0.6 Hz), 7.05 (br d, 2H, *J* = 8.0 Hz), 4.08 (s, 6H), 3.80 (s, 6H); ^{13}C{^{1}H} NMR ($CDCl_3$, 101 MHz): δ 168.9, 156.3, 137.0, 133.4, 120.9 (q, *J* = 320 Hz), 119.3, 116.1, 109.9, 56.6, 47.2; ^{19}F{^{1}H} NMR ($CDCl_3$, 377 MHz): δ –78.3 (s); IR (NaCl, cm^{-1}): 1032, 1143, 1226, 1268, 1275, 1439, 1470, 1574, 1630, 2850, 2958, 3079; HRMS (ESI) *m/z*: [M − OTf]$^+$ Calcd for $C_{17}H_{18}NO_2S^+$ 300.1051; Found 300.1058.

Bis(dibenzo[*b*,*d*]furano)[5,4-*b*:5',4'-*d*]thiopyran-4-one (**3o**)

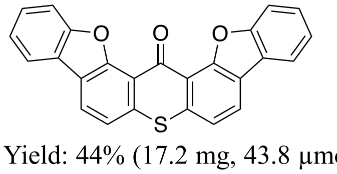

Yield: 44% (17.2 mg, 43.8 μmol); Yellow solid; Mp 315–317 °C; TLC R_f 0.54 (CH_2Cl_2); ^{1}H NMR ($CDCl_3$, 400 MHz): δ 8.10 (d, 2H, *J* = 8.2 Hz), 7.95 (d, 2H, *J* = 7.2 Hz), 7.85 (d, 2H, *J* = 8.2 Hz), 7.58–7.47 (m, 4H), 7.41 (dd,

2H, J = 7.2, 7.2 Hz); $^{13}C\{^1H\}$ NMR ($CDCl_3$, 101 MHz): δ 178.3, 156.7, 155.6, 136.0, 127.6, 124.7, 123.5, 123.4, 122.7, 120.2, 120.0, 117.6, 112.7; IR (NaCl, cm^{-1}) 919, 937, 1012, 1142, 1183, 1319, 1404, 1455, 1540, 1558, 1575, 1621, 1651; HRMS (ESI) *m/z*: $[M + Na]^+$ Calcd for $C_{25}H_{12}NaO_3S^+$ 415.0405; Found 415.0404.

2,3,6,7-Tetramethoxy-9*H*-thioxanthen-9-one 10-oxide (**7**)

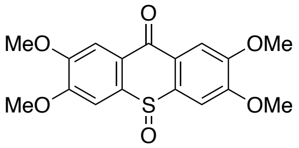

Yield: 68% (49.1 mg, 141 μmol); Colorless solid; Mp 290–291 ℃; TLC R_f 0.37 (CH_2Cl_2/*n*-hexane = 2/1); ^1H NMR ($CDCl_3$, 400 MHz): δ 7.85 (s, 2H), 7.53 (s, 2H), 4.09 (s, 6H), 4.04 (s, 6H); $^{13}C\{^1H\}$ NMR ($CDCl_3$, 101 MHz): δ 177.9, 154.0, 151.7, 138.9, 122.6, 110.9, 109.2, 57.1, 56.8; IR (NaCl, cm^{-1}) 637, 775, 806, 1250, 1283, 1431, 1734; HRMS (ESI) *m/z*: $[M + H]^+$ Calcd for $C_{17}H_{17}O_6S^+$ 349.0740; Found 349.0739.

2,3,6,7-Tetramethoxy-9*H*-thioxanthen-9-one 10,10-dioxide (**8**)

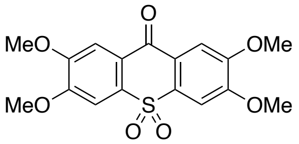

Yield: 90% (44.8 mg, 123 μmol); Yellow solid; Mp 311–312 °C; TLC R_f 0.54 (CH_2Cl_2/acetone = 20/1); ^1H NMR ($CDCl_3$, 400 MHz): δ 7.77 (s, 2H), 7.50 (s, 2H), 4.09 (s, 6H), 4.05 (s, 6H); $^{13}C\{^1H\}$ NMR ($CDCl_3$, 101 MHz): δ 176.3, 154.1, 152.4, 134.8, 124.5, 110.1, 104.8, 56.9, 56.6; IR (NaCl, cm^{-1}) 1041, 1123, 1163, 1233, 1282, 1364, 1401, 1438, 1459, 1514, 1567, 1648; HRMS (ESI) *m/z*: $[M + H]^+$ Calcd for $C_{17}H_{17}O_7S^+$ 365.0690; Found 365.0690.

2,3,6,7-Tetramethoxy-9-(4-methoxyphenyl)thioxanthylium trifluoromethanesulfonate (**10**)

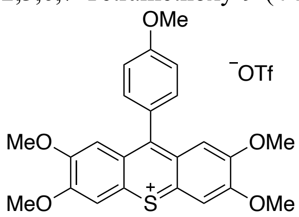

Yield: 77% (46.3 mg, 80.9 μmol); Red solid; Mp 110–112 °C; TLC R_f 0.33 (CH_2Cl_2/methanol = 20/1); ^1H NMR ($CDCl_3$, 400 MHz): δ 8.19 (s, 2H), 7.41–7.36 (AA'BB', 2H), 7.32–7.24 (m, 2H), 7.16 (s, 2H), 4.28 (s, 6H), 4.02 (s, 3H), 3.80 (s, 6H); $^{13}C\{^1H\}$ NMR ($CDCl_3$, 101 MHz): δ 161.0, 159.8, 157.3, 151.7, 143.4, 130.4, 127.5, 125.5, 120.9 (q, J = 320 Hz), 114.8, 110.7, 107.8, 58.3, 56.2, 55.6; $^{19}F\{^1H\}$ NMR ($CDCl_3$, 377 MHz): δ –78.1 (s); IR (NaCl, cm^{-1}) 867, 1009, 1029, 1072, 1223, 1249, 1278, 1359, 1428, 1505, 1603; HRMS (ESI) *m/z*: $[M − OTf]^+$ Calcd for $C_{24}H_{23}O_5S^+$ 423.1261; Found 423.1261.

4-Iododibenzo[*b*,*d*]furan-3-ol

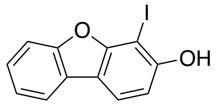

Yield: 43% (806 mg, 2.60 mmol); Colorless solid; Mp 138–139 ℃; TLC R_f 0.65 (CH_2Cl_2); ^1H NMR ($CDCl_3$, 400 MHz): δ 7.86 (d, 1H, J = 7.9 Hz), 7.76 (d, 1H, J = 8.3 Hz), 7.63 (d, 1H, J = 7.9 Hz), 7.43 (dd, 1H, J = 7.9, 7.9 Hz), 7.36 (dd, 1H, J = 7.9, 7.9 Hz), 7.04 (d, 1H, J = 8.3 Hz), 5.58 (s, 1H); $^{13}C\{^1H\}$ NMR ($CDCl_3$, 101 MHz): δ 157.2, 155.9, 155.1, 126.3, 124.9, 123.3, 121.3, 120.3, 117.7, 111.8, 110.7, 67.1; IR (NaCl, cm^{-1}) 986, 1023, 1087, 1126, 1179, 1220, 1372, 1409, 1470; HRMS (ESI) *m/z*: $[M − H]^-$ Calcd for $C_{12}H_6IO_2^-$ 308.9413; Found 308.9412.

4-(Trimethylsilyl)dibenzo[*b*,*d*]furan-3-yl trifluoromethanesulfonate (**1p**)

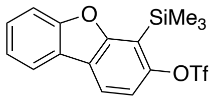

Yield: 60% (606 mg, 1.56 mmol); Yellow solid; Mp 59–60 °C; TLC R_f 0.70 (CH_2Cl_2/*n*-hexane = 2/1); ^1H NMR ($CDCl_3$, 400 MHz): δ 8.00–7.92 (m, 2H), 7.59 (d, 1H, J = 7.8, 0.8 Hz), 7.50 (ddd, 1H, J = 7.8, 7.8, 0.8 Hz), 7.38 (ddd, 1H, J = 7.8, 7.8, 0.8 Hz), 7.34 (d, 1H, J = 8.5 Hz), 0.57 (s, 9H); $^{13}C\{^1H\}$ NMR ($CDCl_3$, 101 MHz): δ 160.9, 156.9, 153.0, 127.9, 123.4, 123.2, 123.0, 122.4, 120.9, 119.0 (q, J = 320 Hz), 117.1, 115.6, 111.9, 0.6; $^{19}F\{^1H\}$

NMR ($CDCl_3$, 377 MHz): δ –73.0 (s); IR (NaCl, cm^{-1}) 843, 954, 1110, 1140, 1193, 1215, 1251, 1387, 1418, 1422, 1445, 1461, 1580, 2903, 2959, 3065; HRMS (ESI) *m/z*: $[M + Na]^+$ Calcd for $C_{16}H_{15}F_3NaO_4SSi^+$ 411.0310; Found 411.0317.

O,*O*-Dibenzyl carbonothioate (**2a**)

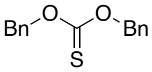

Colorless solid; Mp 69–70 °C; TLC R_f 0.70 (CH_2Cl_2/*n*-hexane = 2/1); ^1H NMR ($CDCl_3$, 400 MHz): δ 7.35–7.46 (m, 10H), 5.52 (s, 4H); $^{13}C\{^1H\}$ NMR ($CDCl_3$, 101 MHz) δ 195.1, 134.5, 128.7, 128.64, 128.60, 74.8; IR (NaCl, cm^{-1}) 849, 907, 934, 1029, 1080, 1225, 1385, 1454, 1498, 2928, 2950, 3035; HRMS (ESI) *m/z*: $[M + Na]^+$ Calcd for $C_{15}H_{14}NaO_2S^+$ 281.0612; Found 281.0612.

References for Supporting Information

S1 C. Shen, G. Yang, W. Zhang, *Org. Lett.* **2013**, *15*, 5722.

S2 B. S. Shaibu, R. K. Kawade, R. S. Liu, *Org. Biomol. Chem.* **2012**, *10*, 6834.

S3 X. Li, Y. Sun, X. Huang, L. Zhang, L. Kong, B. Peng, *Org. Lett.* **2017**, *19*, 838.

S4 S. Yoshida, K. Shimomori, T. Nonaka, T. Hosoya, *Chem. Lett.* **2015**, *44*, 1324.

S5 S. Yoshida, Y. Nakamura, K. Uchida, Y. Hazama, T. Hosoya, *Org. Lett.* **2016**, *18*, 6212.

S6 Jose M. Medina, Joel L. Mackey, Neil K. Garg, K. N. Houk, *J. Am. Chem. Soc.* **2014**, *136*, 15798.

S7 W. Bao, Z. P. Gao, D. P. Jin, C. G. Xue, H. Liang, L. S. Lei, X. T. Xu, K. Zhang, S. H. Wang, *Chem. Commun*. **2020**, *56*, 7641.

S8 Y. Nakamura, Y. Miyata, K. Uchida, S. Yoshida, T. Hosoya, *Org. Lett.* **2019**, *21,* 5252.

S9 N. Aoyagi, T. Endo, *J. Polym. Sci. A Polym. Chem*. **2008**, *47*, 3702.

S10 H. Sugimoto, I. Makino, K. Hirai, *J. Org. Chem*. **1988**, *53*, 2263.

S11 P. Sokołowska, K. Dąbrowa, S. Jarosz, *Org. Lett.* **2021**, *23*, 2687.

S12 A. Venkanna, P. V. K. Goud, P. V. Prasad, M. Shanker, P. V. Rao, *ChemistrySelect* **2016**, *1,* 2271.

S13 T. Yamaguchi, Y. Yamamoto, Y. Fujiwara, Y. Tanimoto, *Org. Lett.* **2005**, *7*, 2739.

^{1}H and ^{13}C NMR Spectra of Compounds

^{1}H NMR (400 MHz) and ^{13}C NMR (101 MHz) spectra of 2,3,6,7-tetramethyl-9*H*-thioxanthen-9-one (**3b**) ($CDCl_3$)

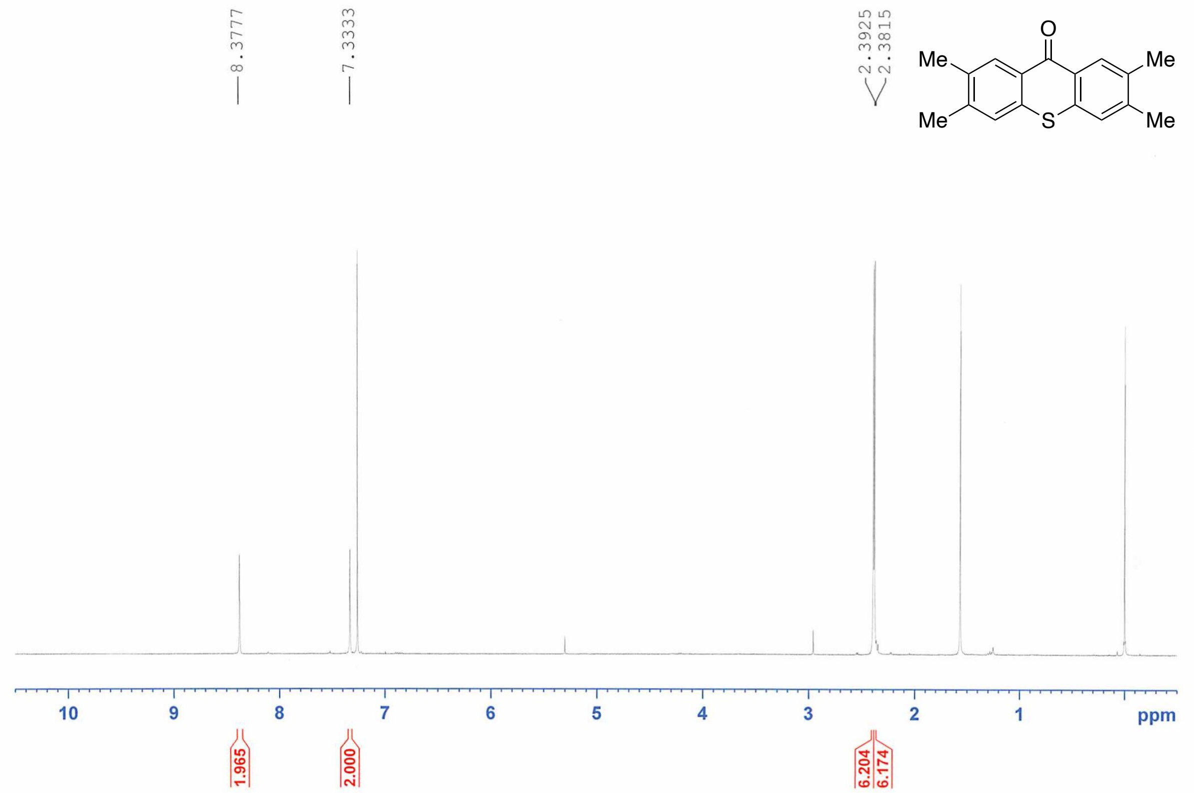

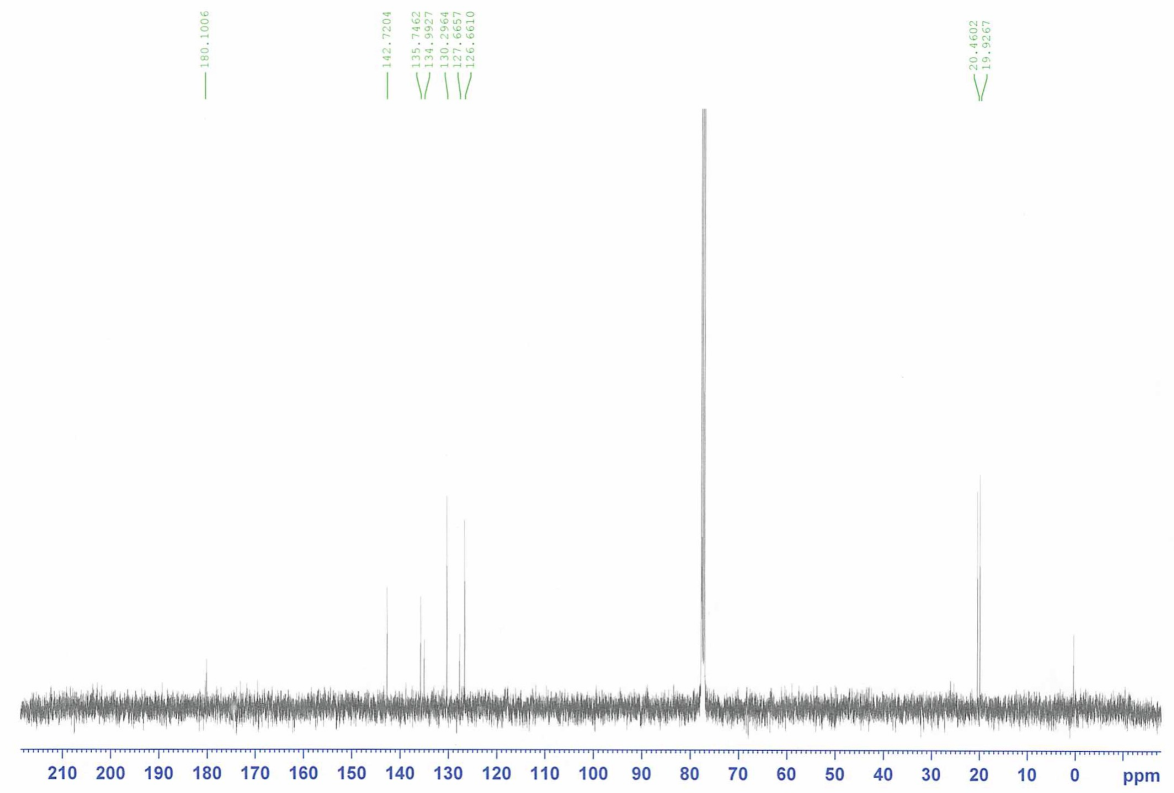

^{1}H NMR (400 MHz) and ^{13}C NMR (101 MHz) spectra of 2,3,6,7-tetramethoxy-9*H*-thioxanthen-9-one (**3c**) ($CDCl_3$)

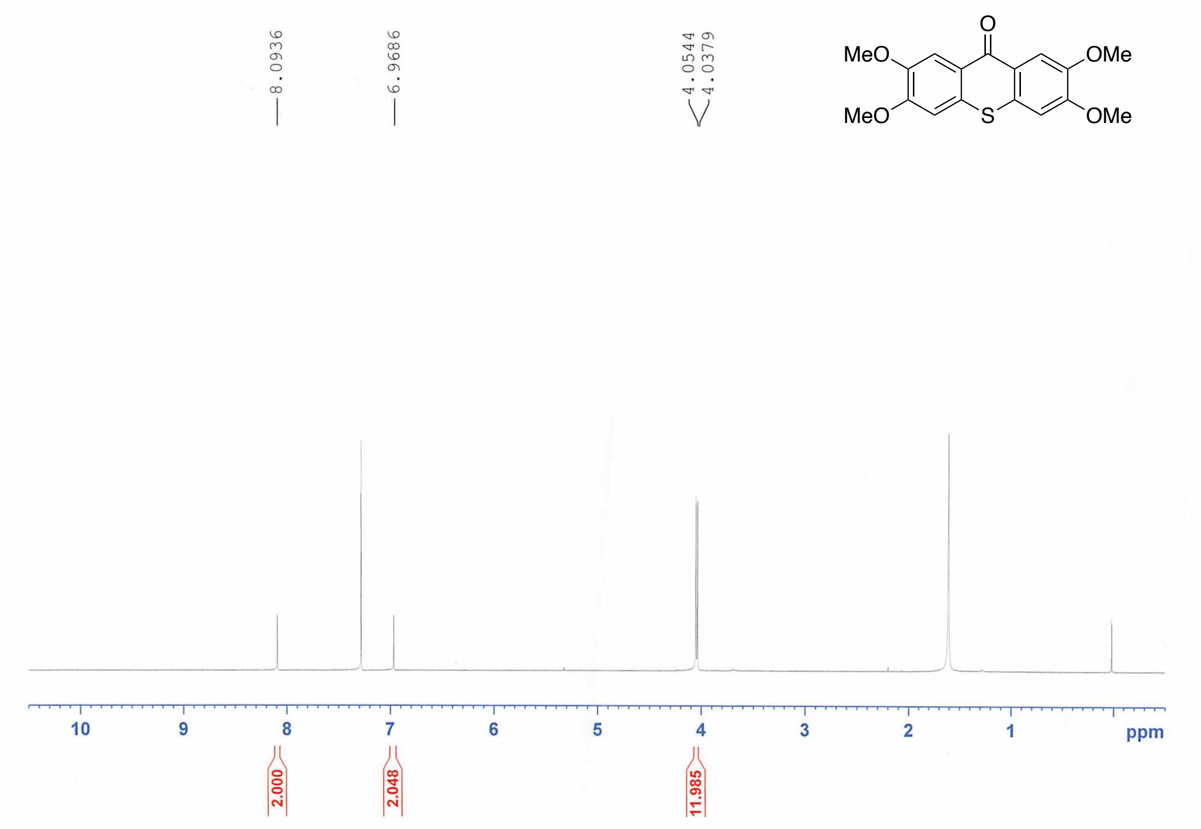

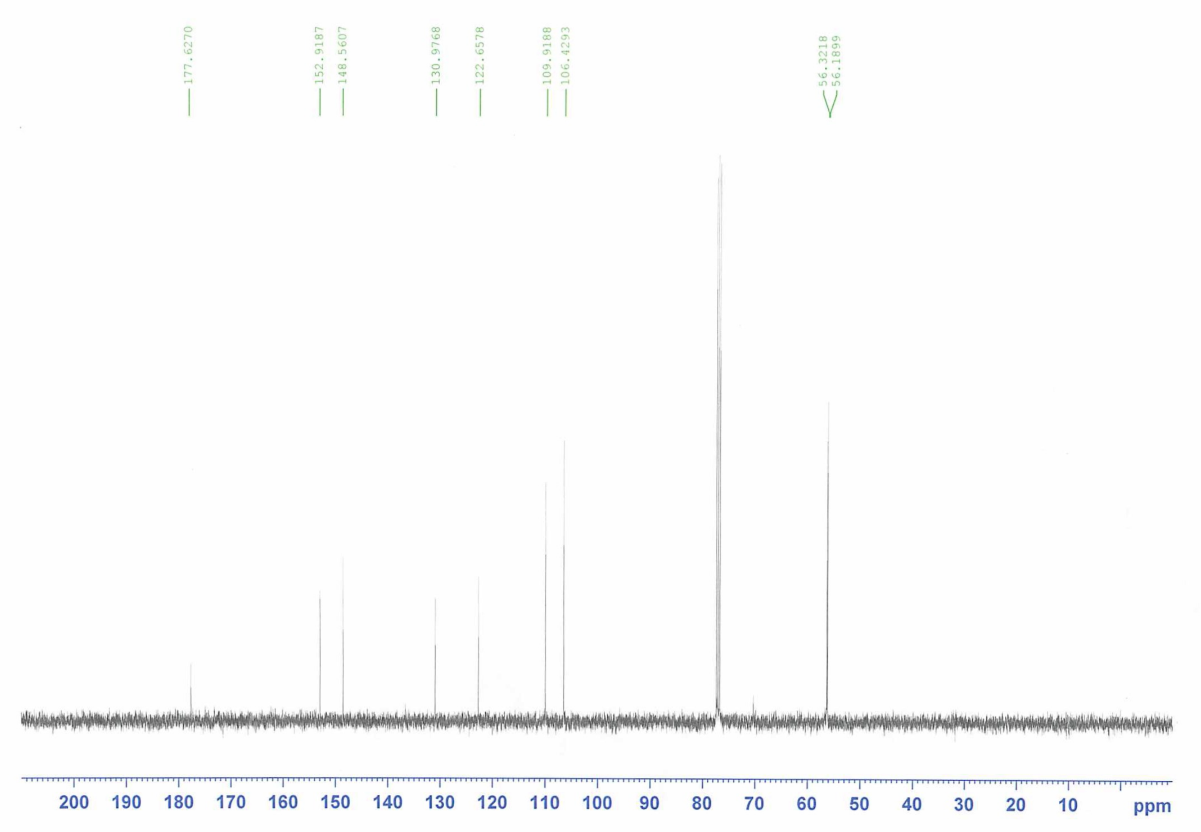

^1H NMR (400 MHz) and ^{13}C NMR (101 MHz) spectra of 2,3,6,7-tetrafluoro-9*H*-thioxanthen-9-one (**3d**) ($CDCl_3$)

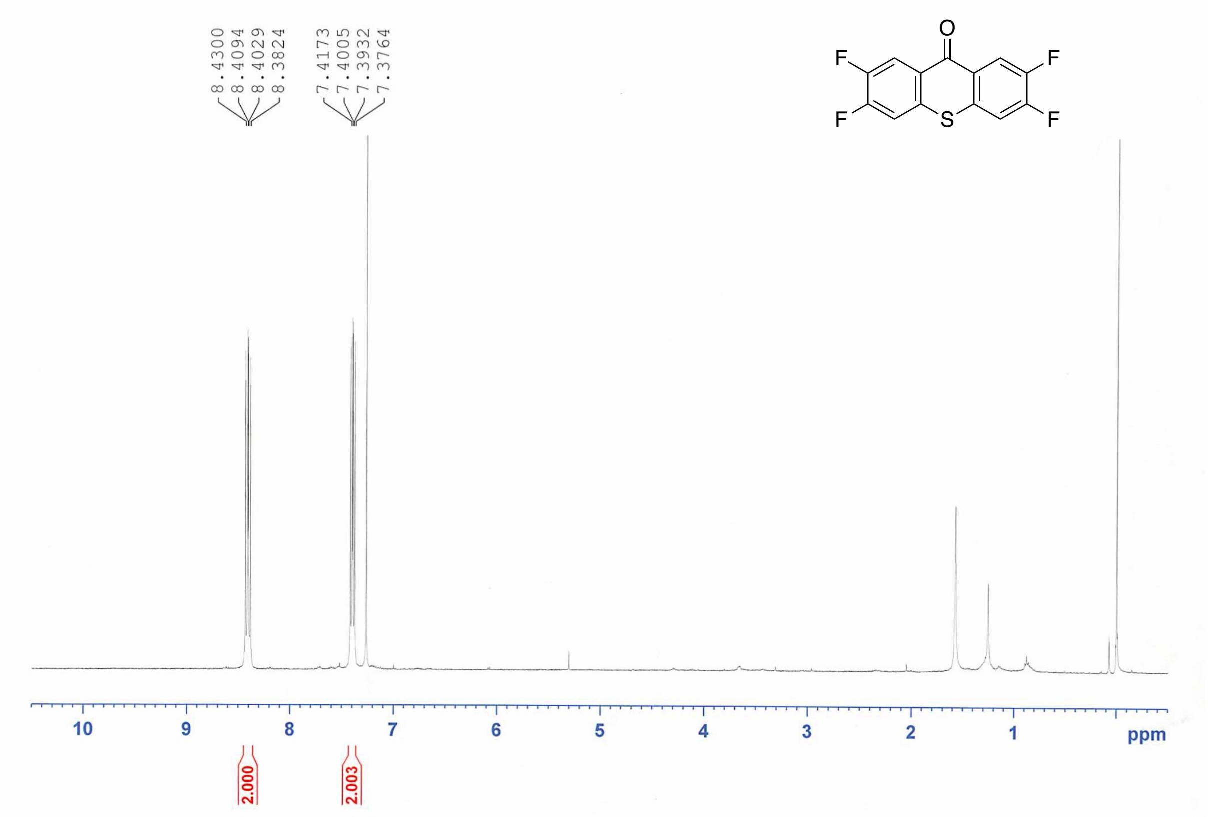

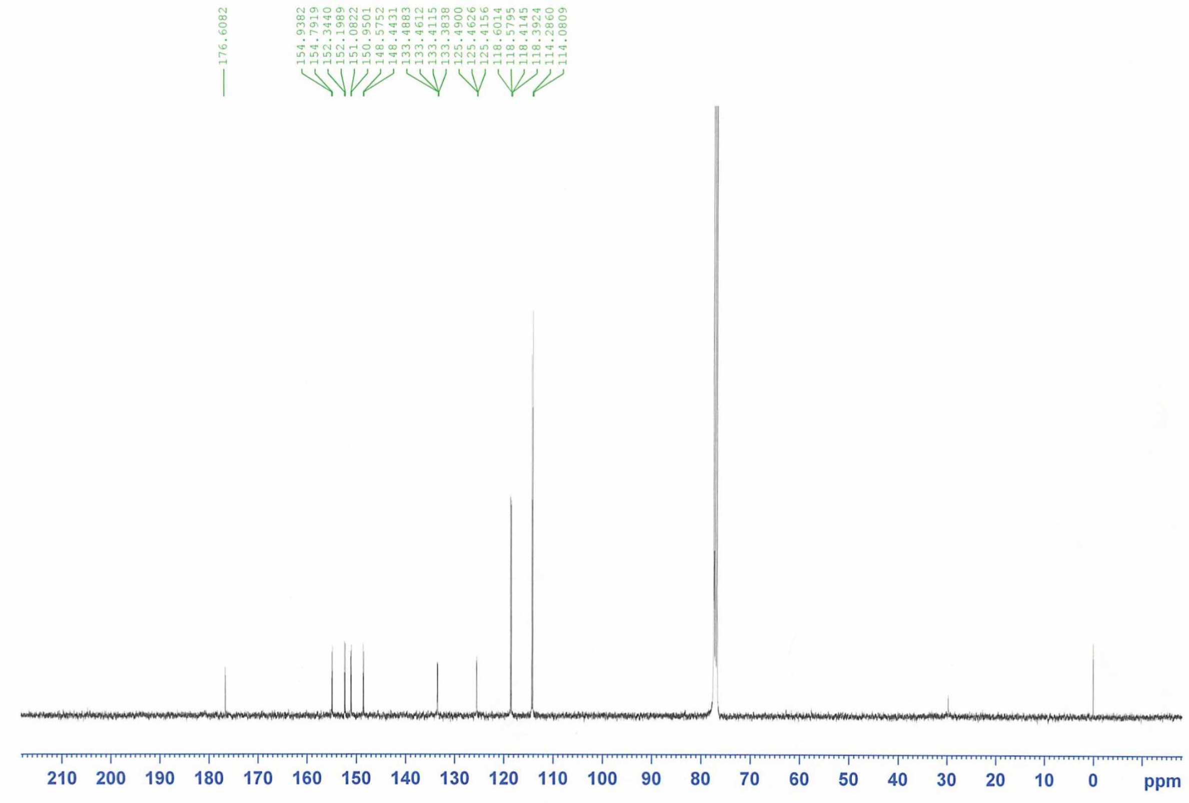

^{1}H NMR (400 MHz) and ^{13}C NMR (101 MHz) spectra of 3,6-dibromo-1,8-dimethoxy-9*H*-thioxanthen-9-one (**3f**) ($CDCl_3$)

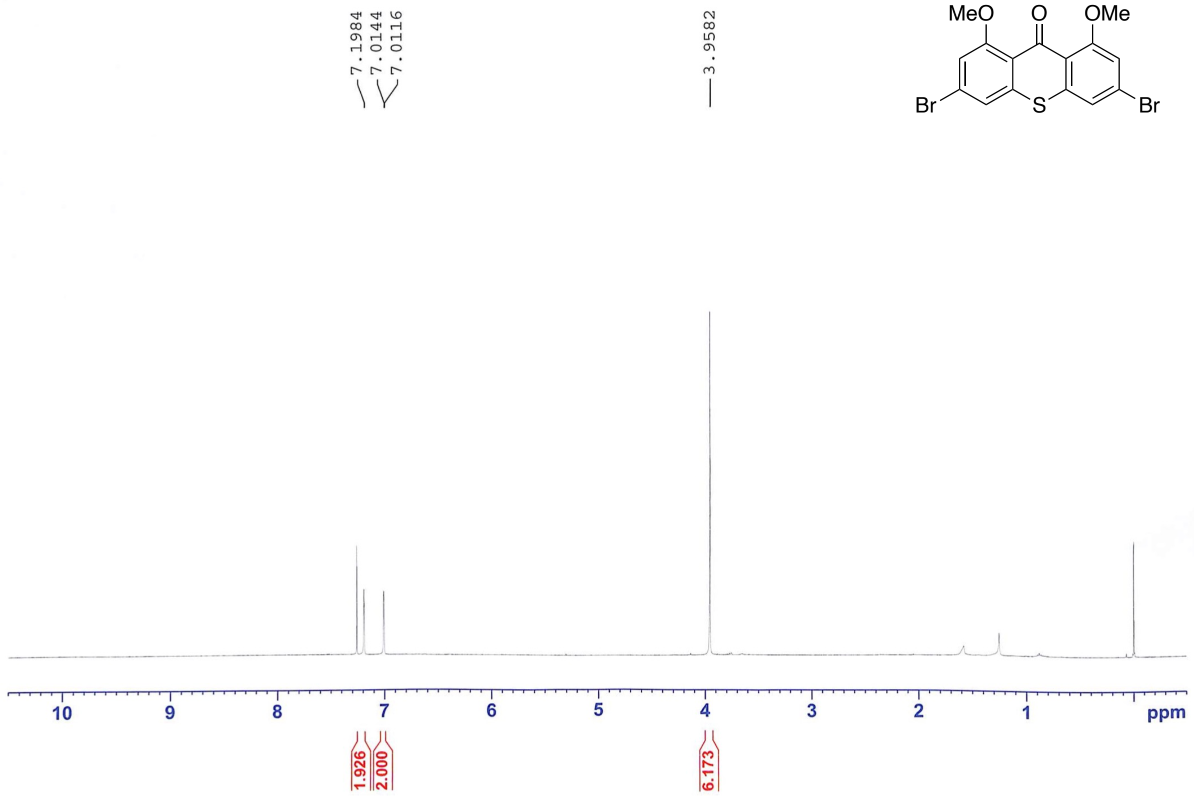

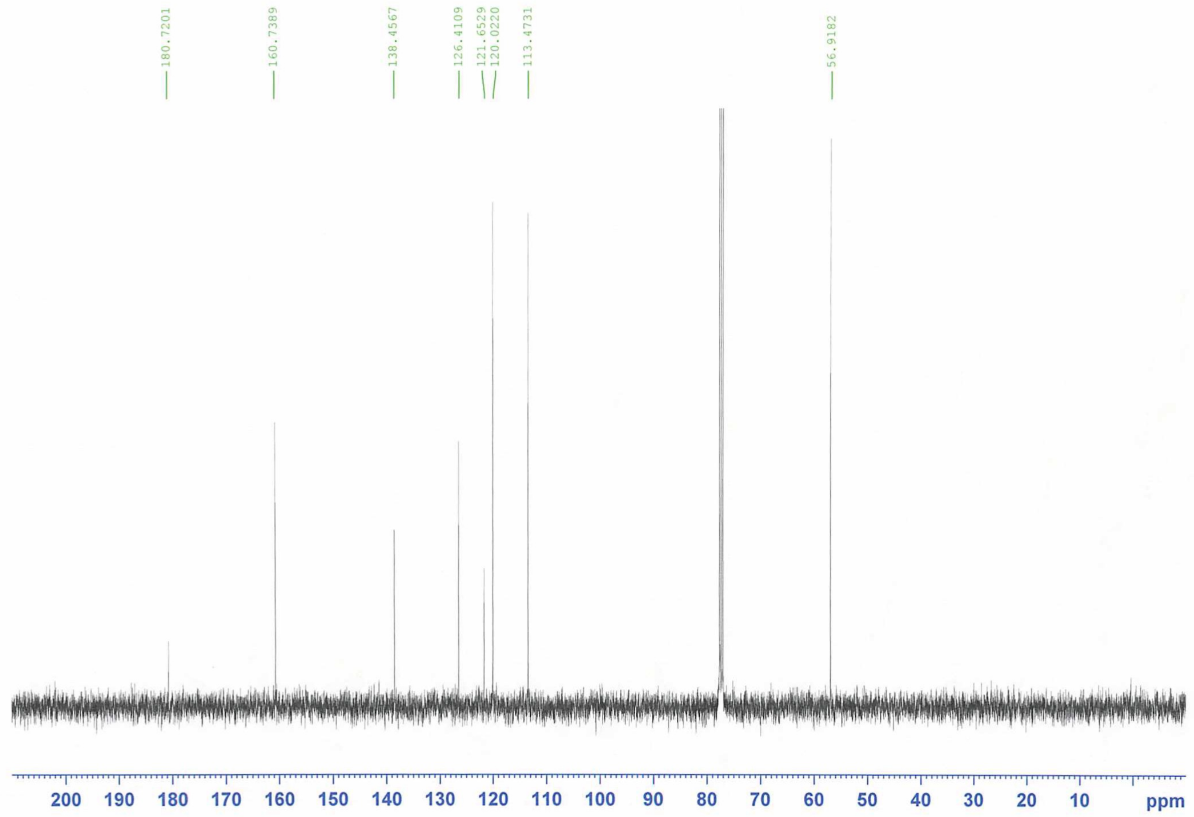

^{1}H NMR (400 MHz) and ^{13}C NMR (101 MHz) spectra of 1,8-dimethoxy-3,6-diphenyl-9*H*-thioxanthen-9-one (**3g**) ($CDCl_3$)

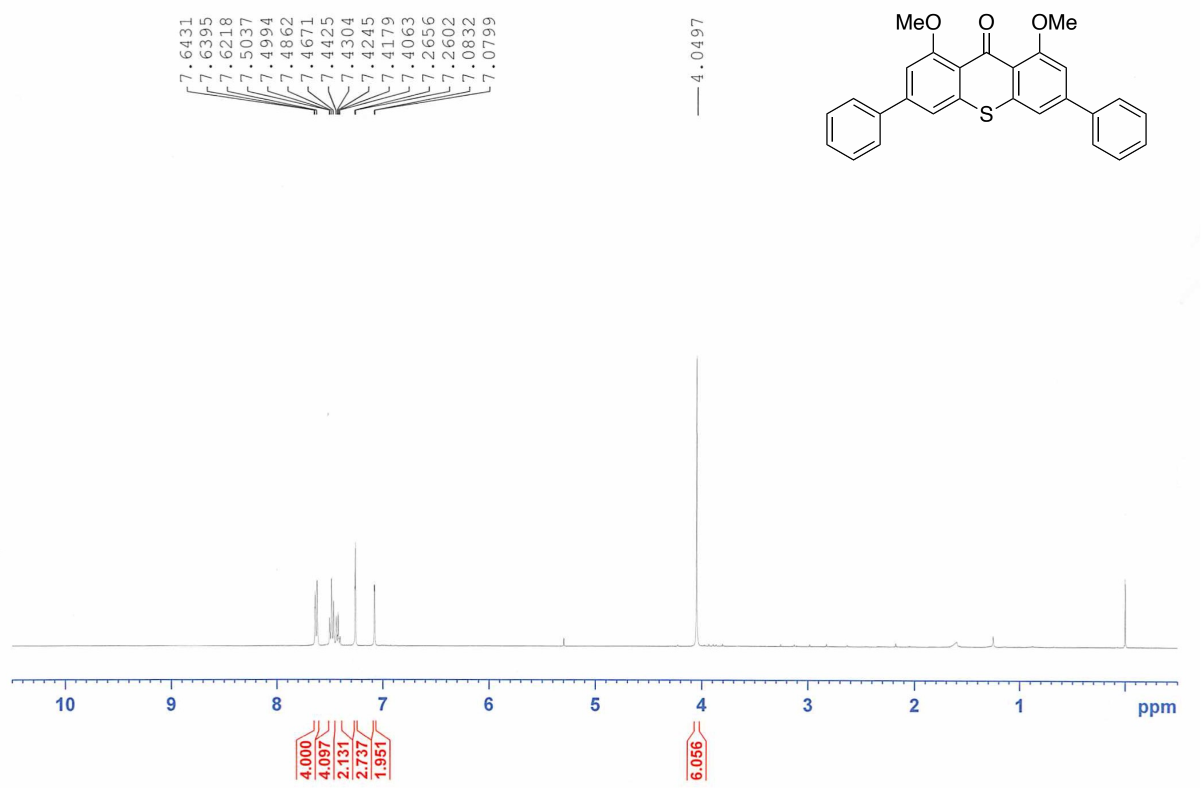

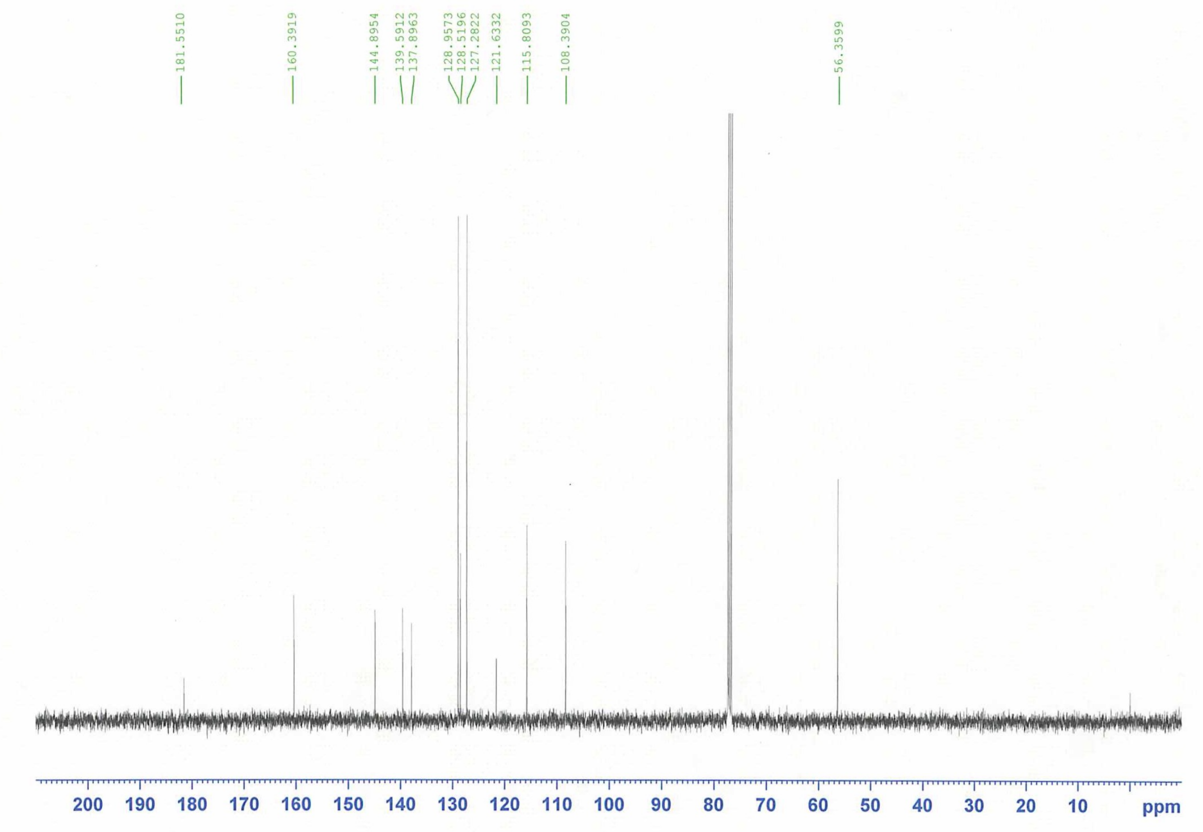

^{1}H NMR (400 MHz) and ^{13}C NMR (101 MHz) spectra of 1,8-dimorpholino-9*H*-thioxanthen-9-one (**3h**) ($CDCl_3$)

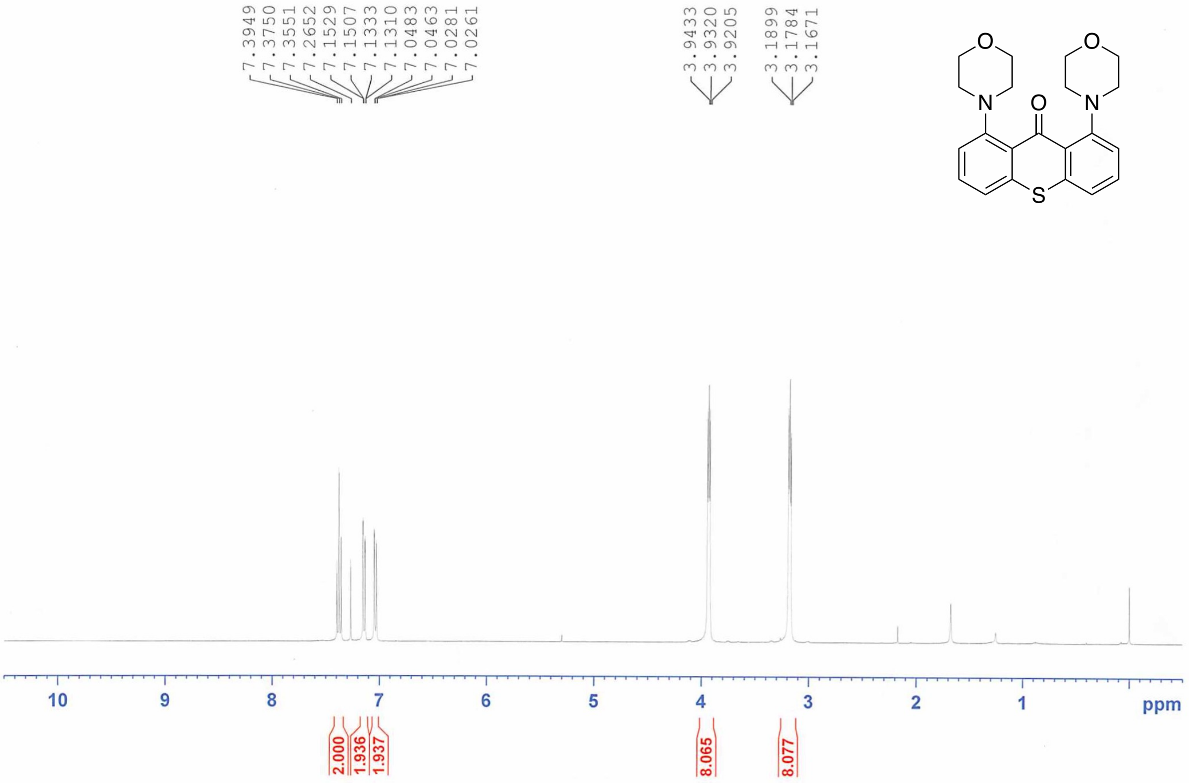

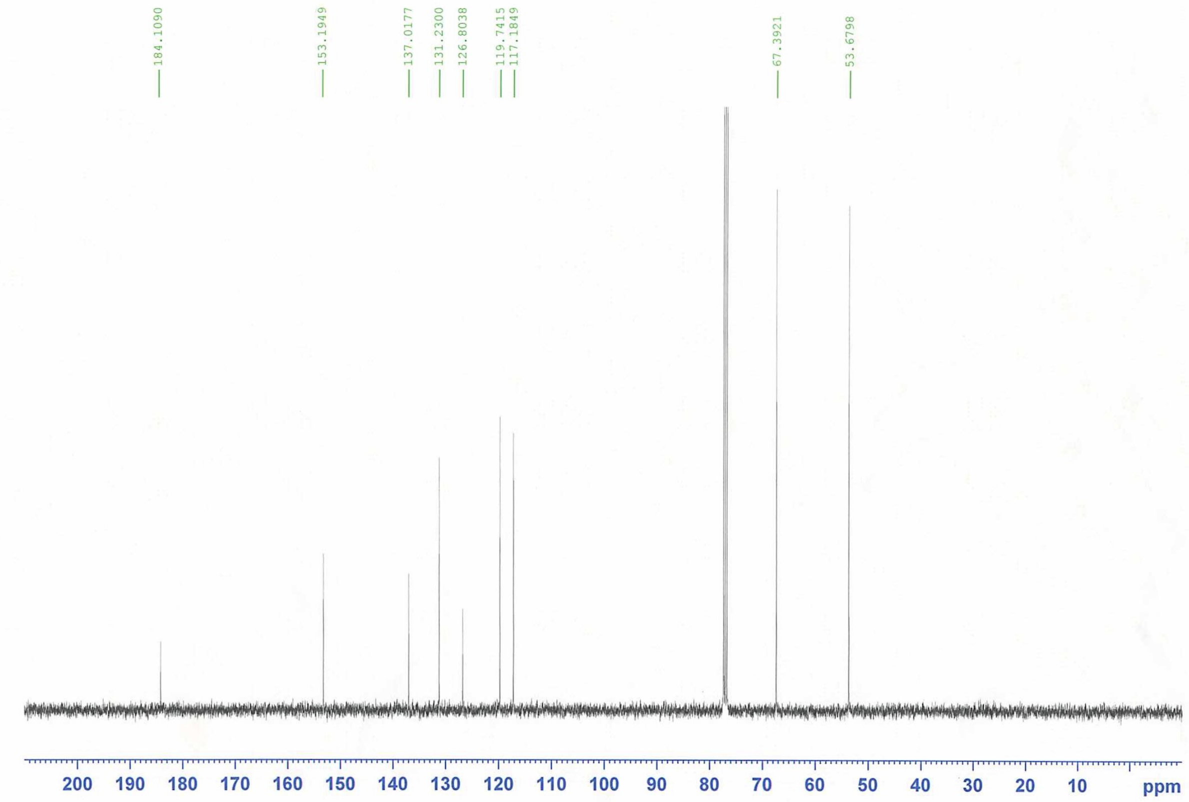

^{1}H NMR (400 MHz) and ^{13}C NMR (101 MHz) spectra of 1,8-bis(dimethylamino)-9*H*-thioxanthen-9-one (**3i**) ($CDCl_3$)

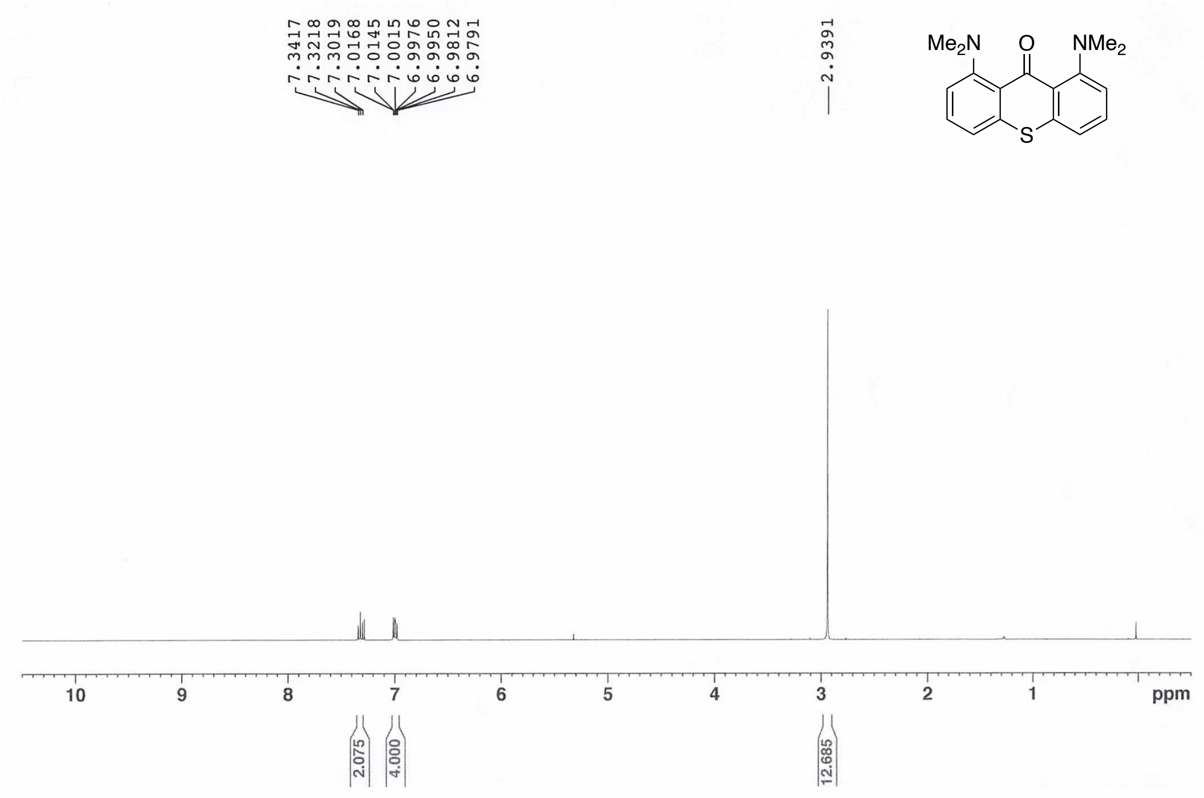

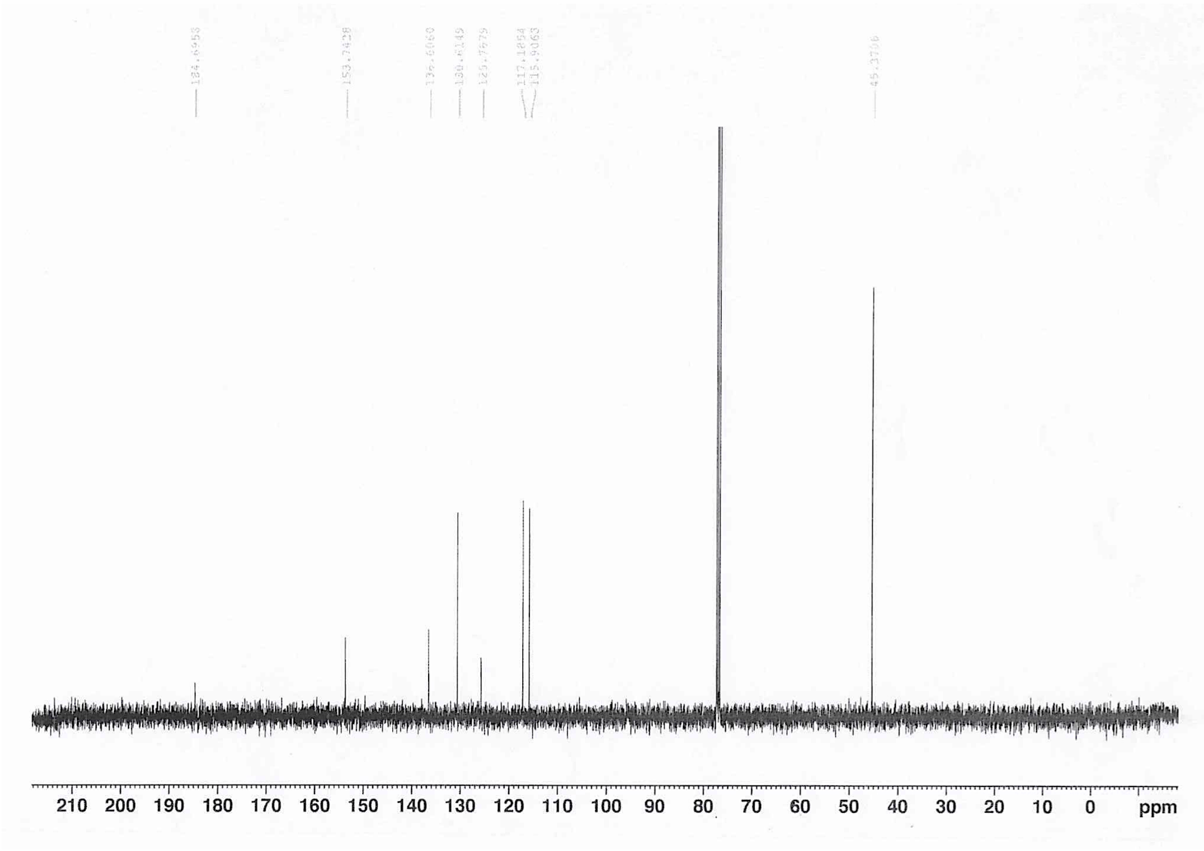

^{1}H NMR (400 MHz) and ^{13}C NMR (101 MHz) spectra of 3,6-dimethyl-9*H*-thioxanthen-9-one (**3k**) ($CDCl_3$)

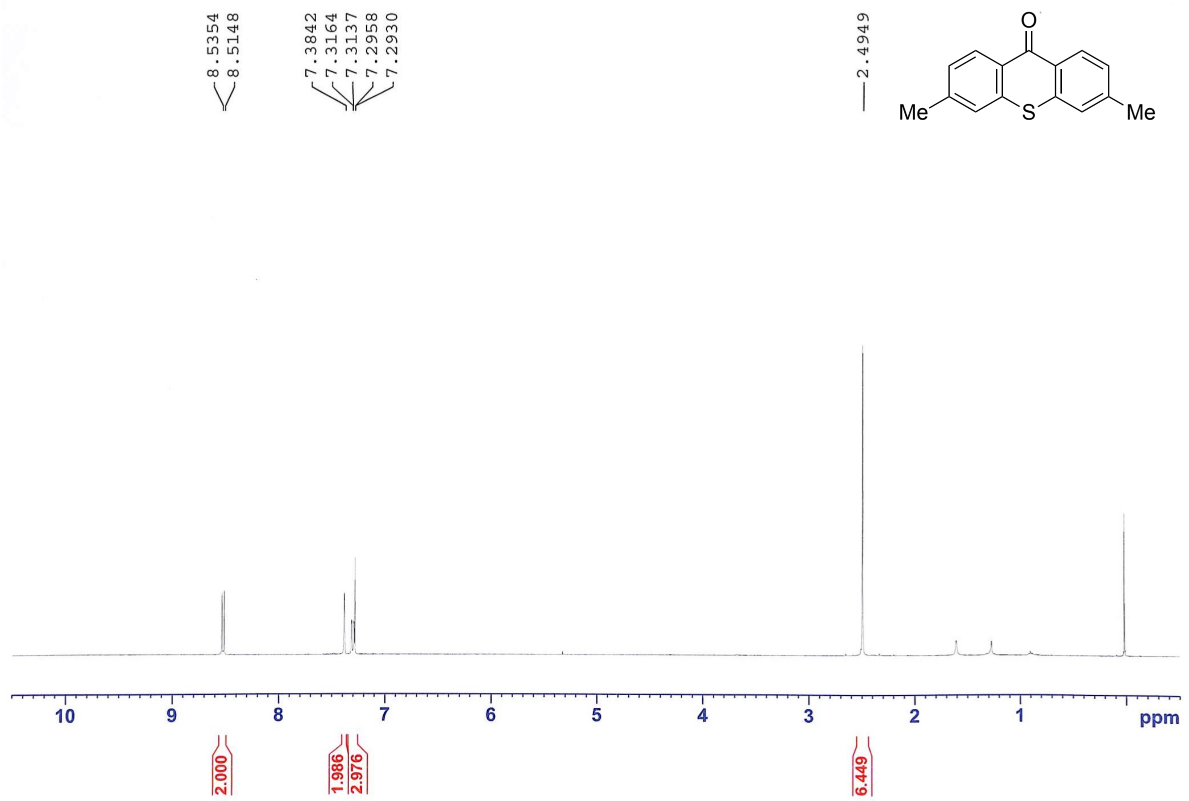

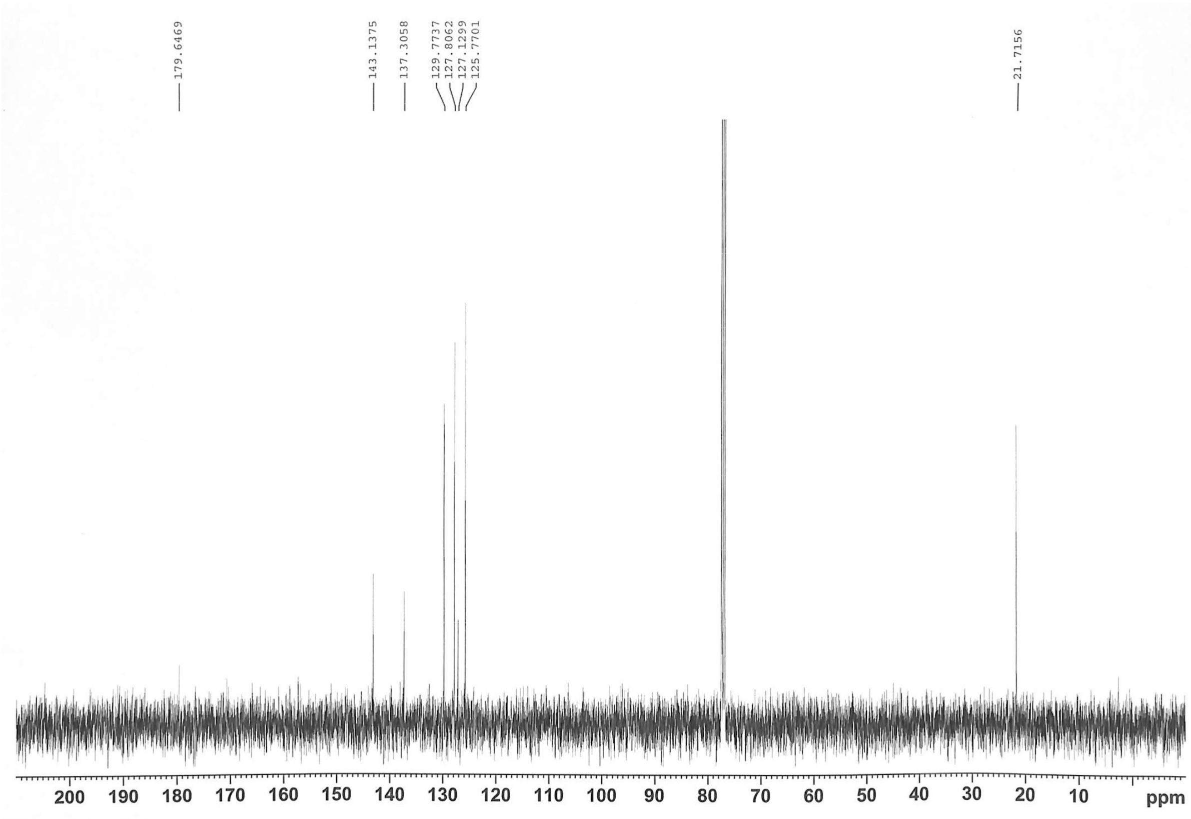

^{1}H NMR (400 MHz) and ^{13}C NMR (101 MHz) spectra of 2,6-dimethyl-9*H*-thioxanthen-9-one (**3k'**) ($CDCl_3$)

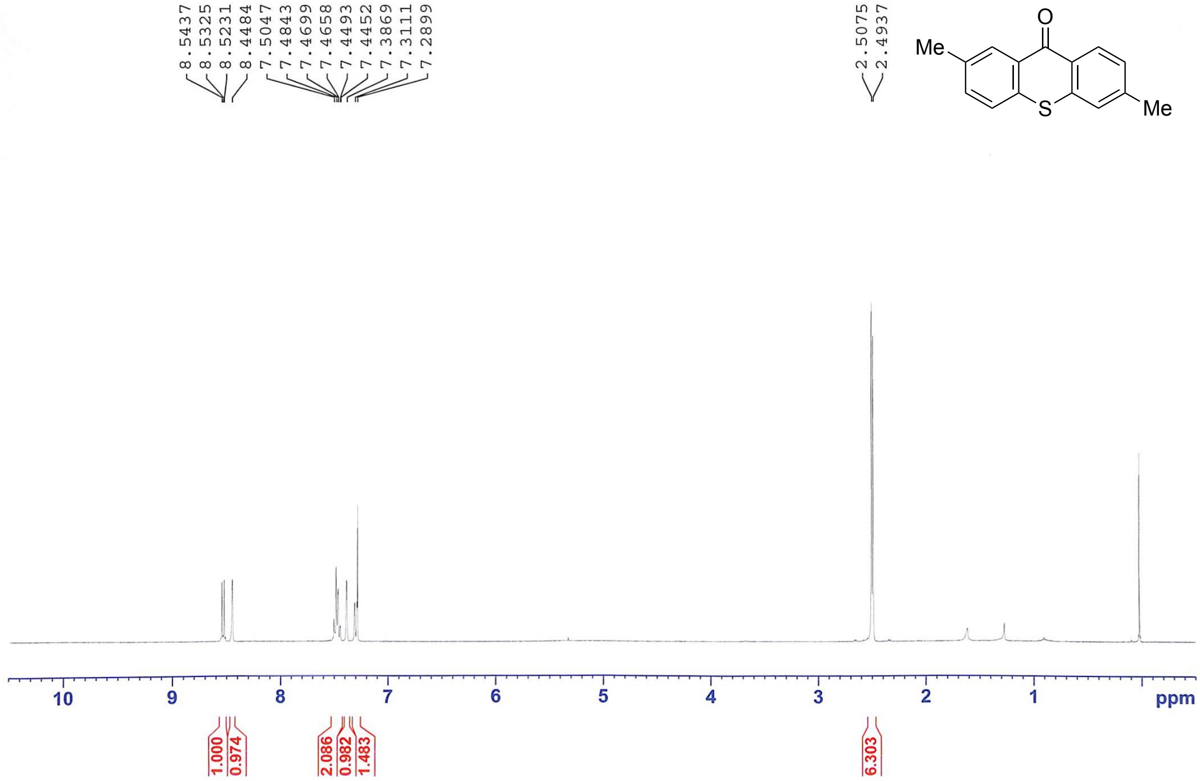

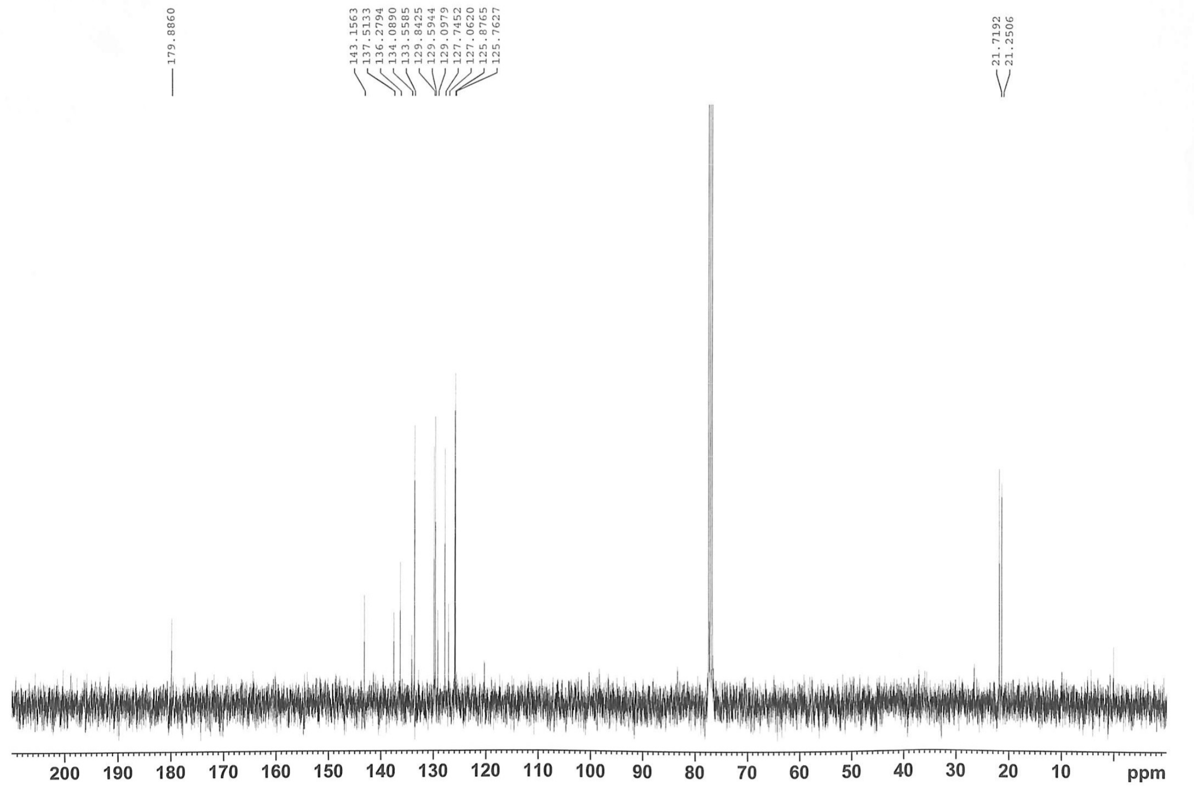

^{1}H NMR (400 MHz) and ^{13}C NMR (101 MHz) spectra of 2,7-dimethyl-9*H*-thioxanthen-9-one (**3k''**) ($CDCl_3$)

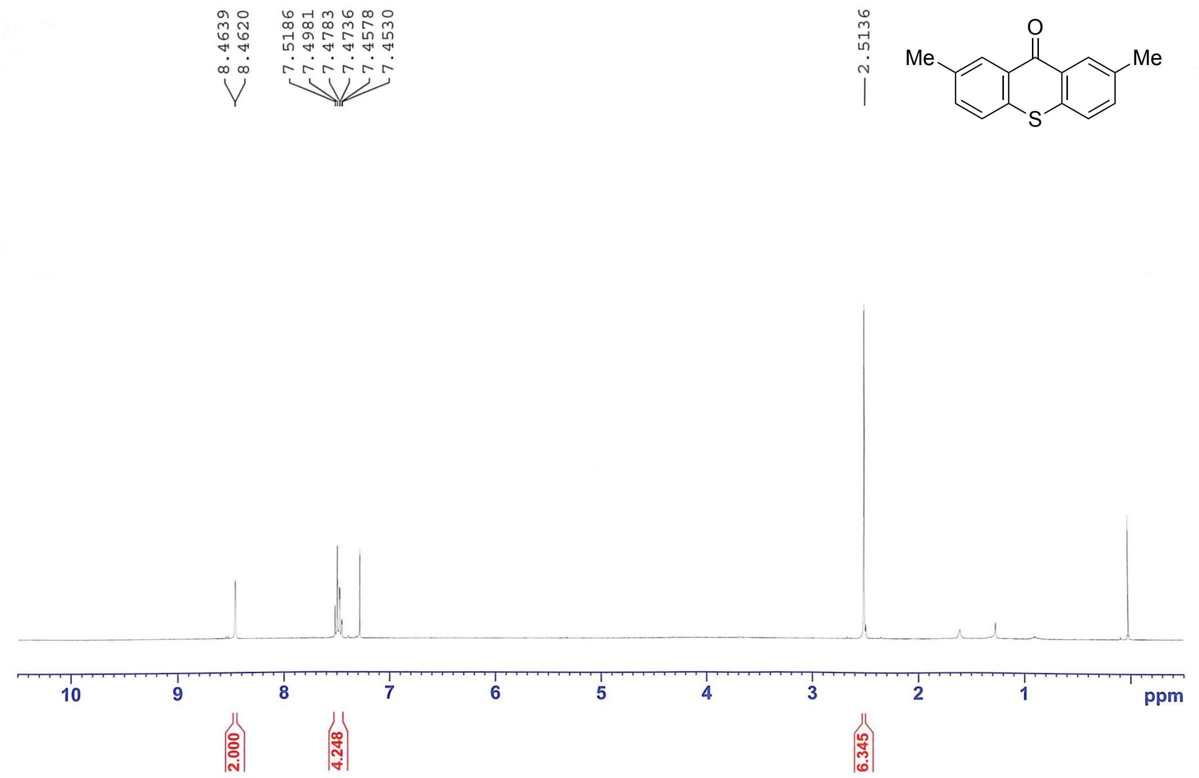

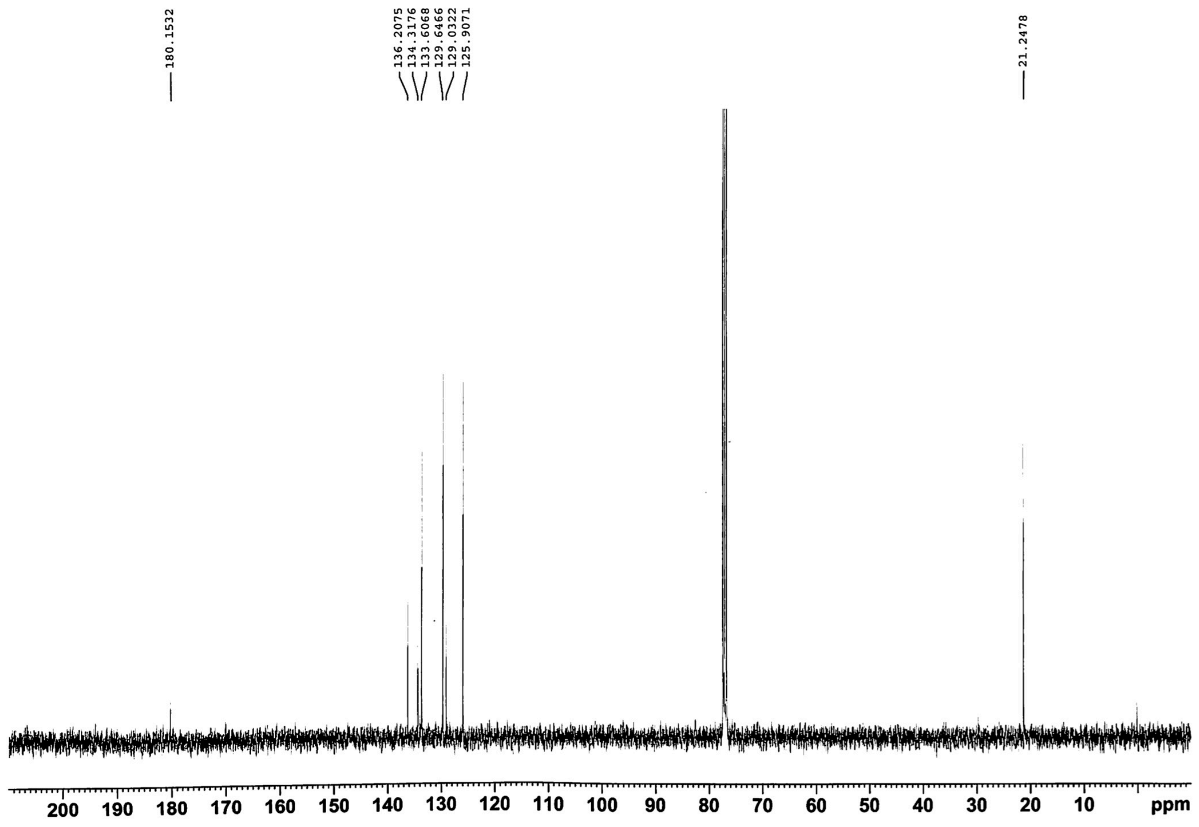

^{1}H NMR (400 MHz) and ^{13}C NMR (101 MHz) spectra of 2,3,8,9-tetrahydro-1*H*-dicyclopenta[*b*,*i*]thioxanthen-11(7*H*)-one (**3l**) ($CDCl_3$)

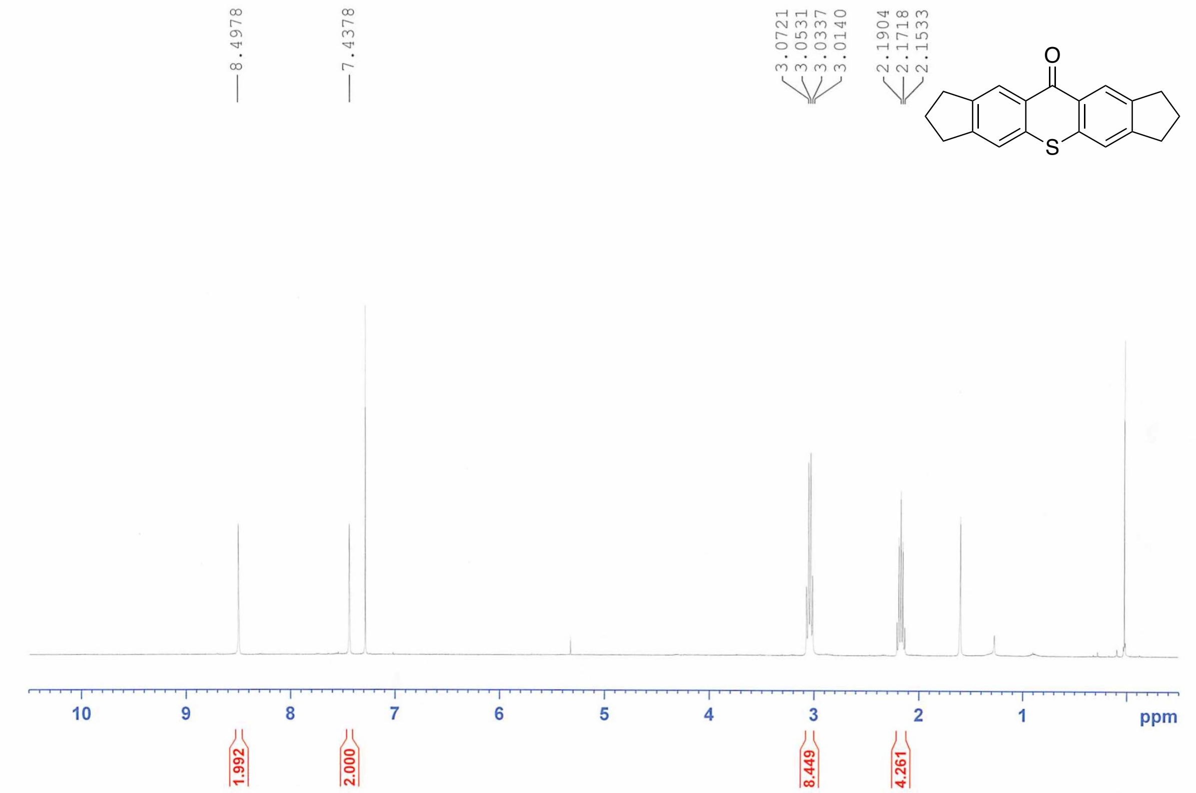

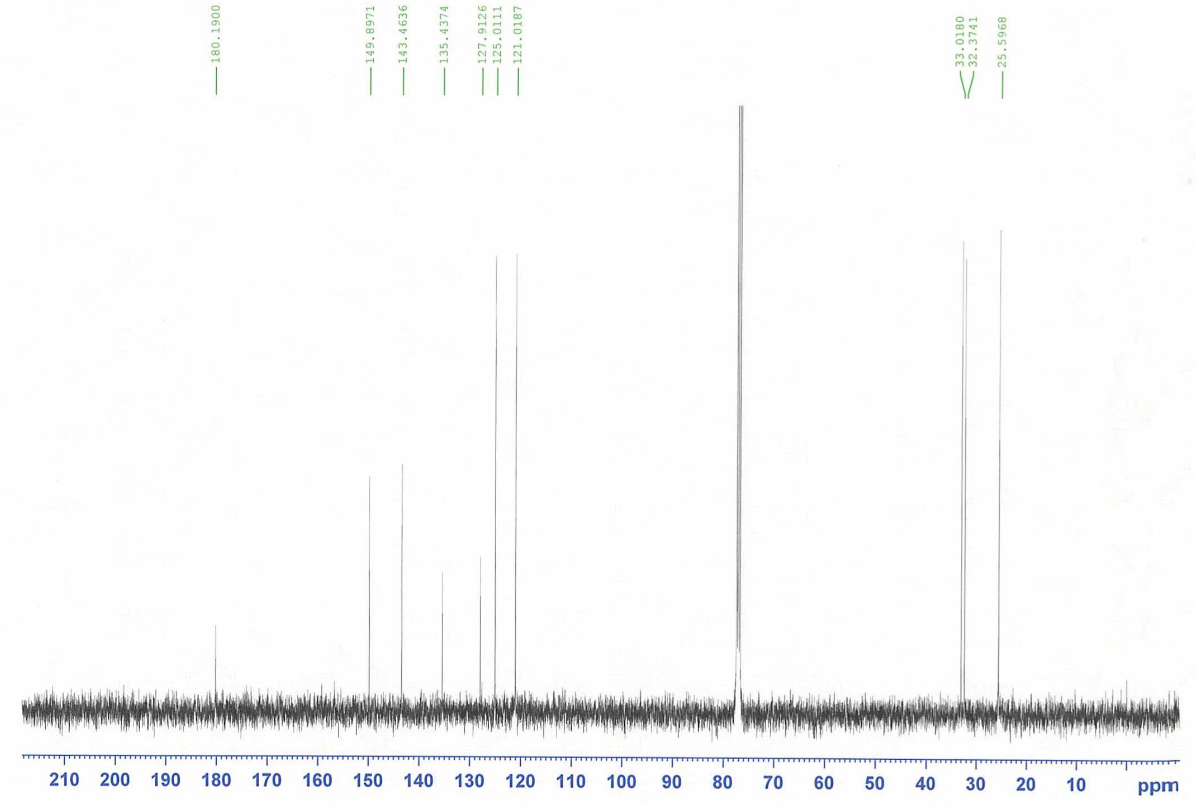

^{1}H NMR (400 MHz) and ^{13}C NMR (101 MHz) spectra of 11*H*-thioxantheno[2,3-*d*:6,7-*d'*]bis([1,3]dioxole)-11-one (**3m**) ($CDCl_3$)

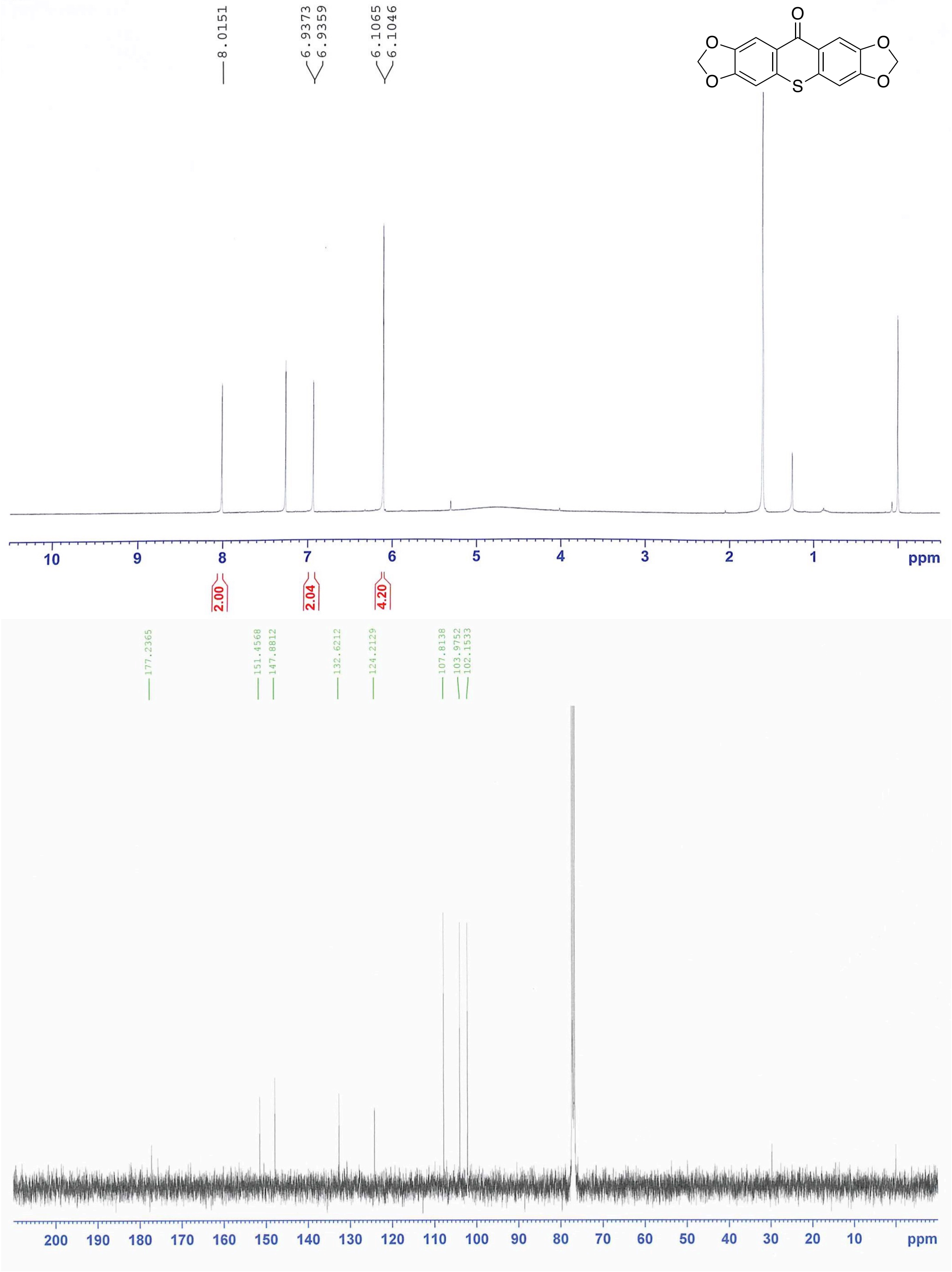

^{1}H NMR (400 MHz) and ^{13}C NMR (101 MHz) spectra of 13*H*-dibenzo[*b*,*i*] thioxanthene-13-one (**3n**) ($CDCl_3$)

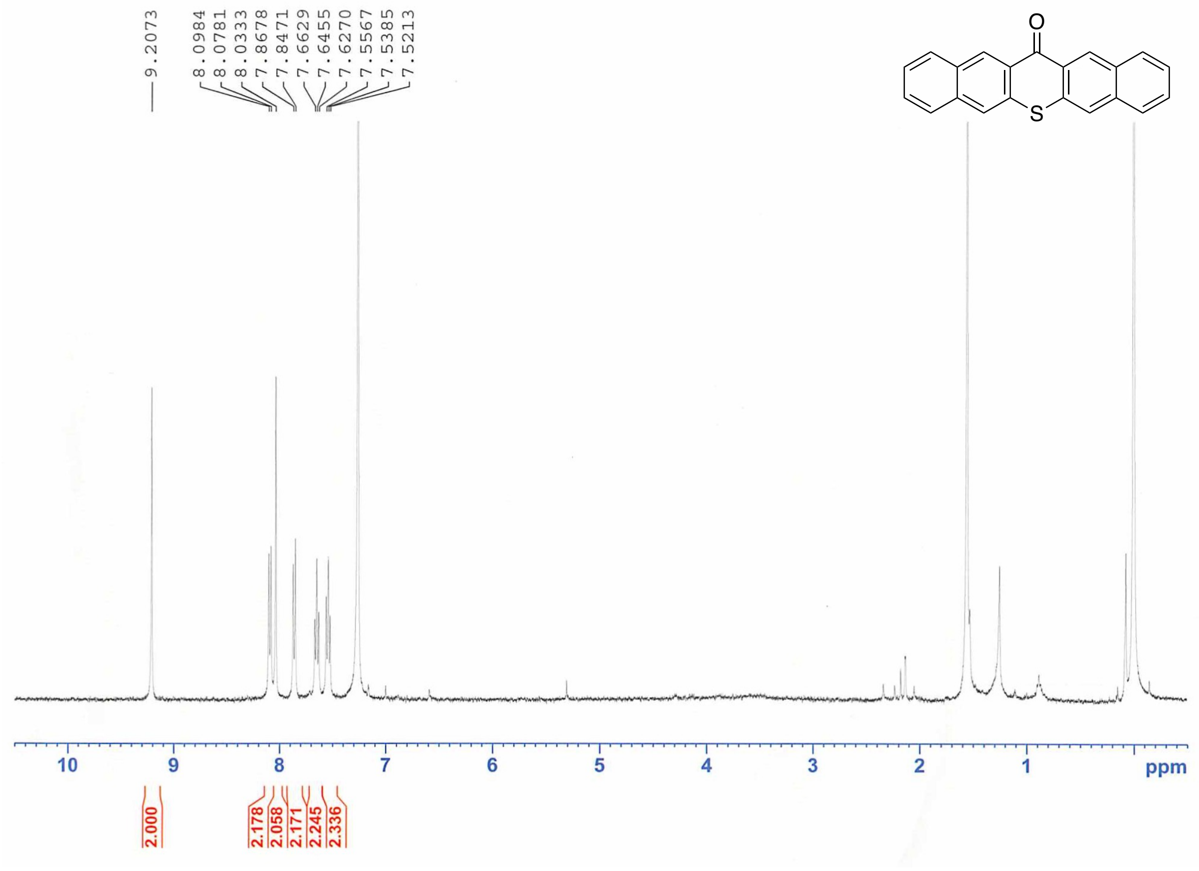

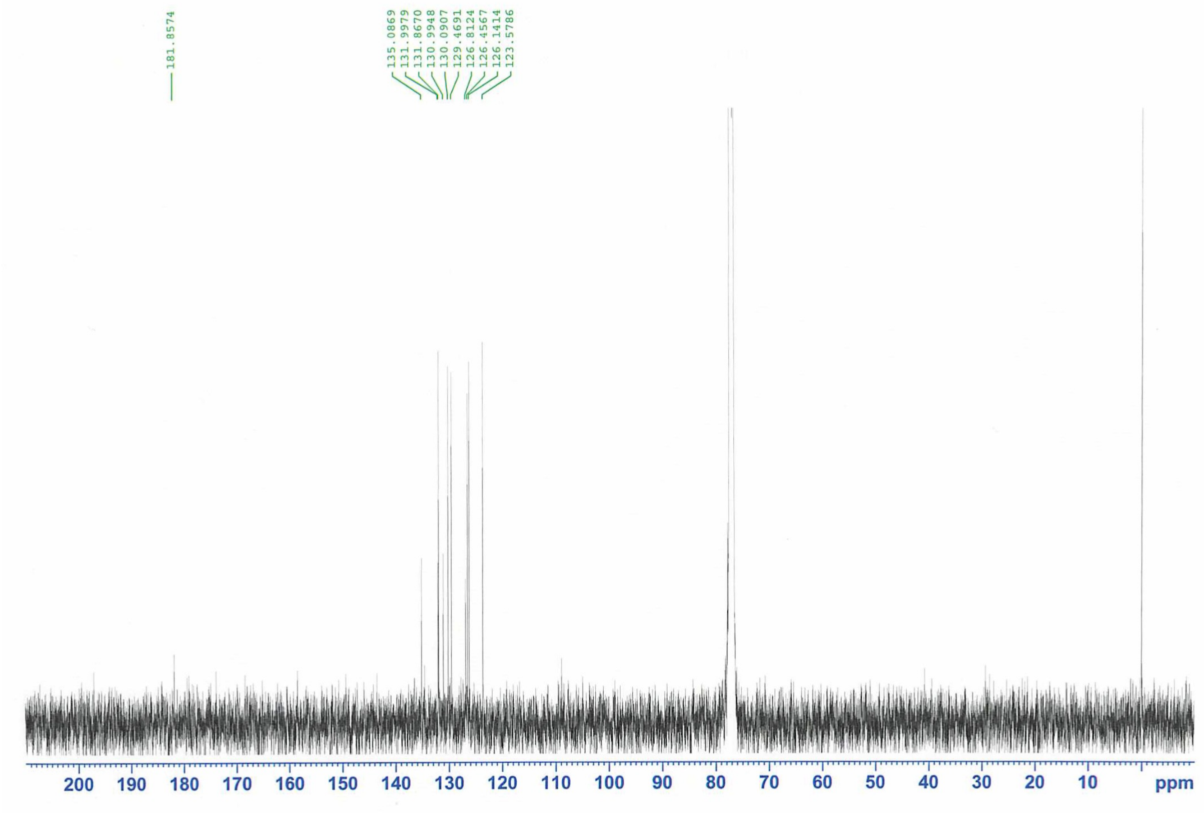

^{1}H NMR (400 MHz) and ^{13}C NMR (101 MHz) spectra of *N*-(1,8-bis((4-methoxyphenyl)thio)-9*H*-thioxanthen-9-ylidene)-*N*-methylmethanaminium trifluoromethanesulfonate (**4a**) ($CDCl_3$)

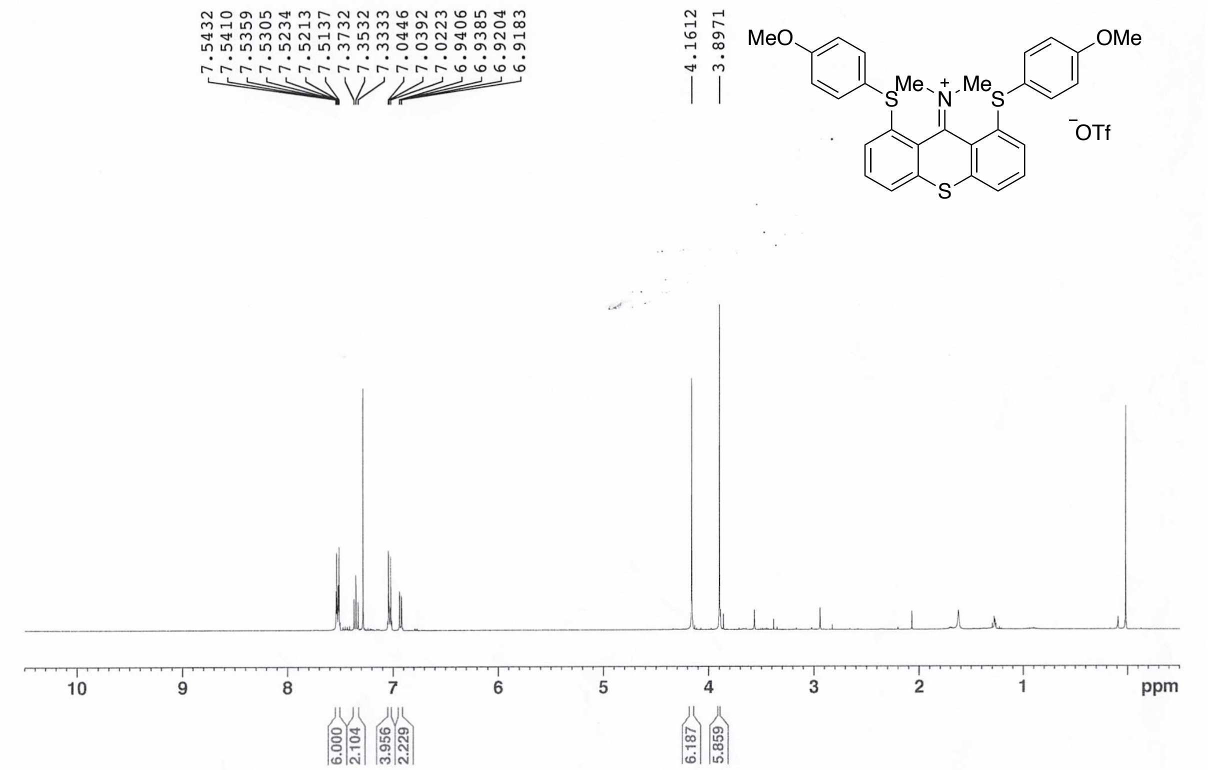

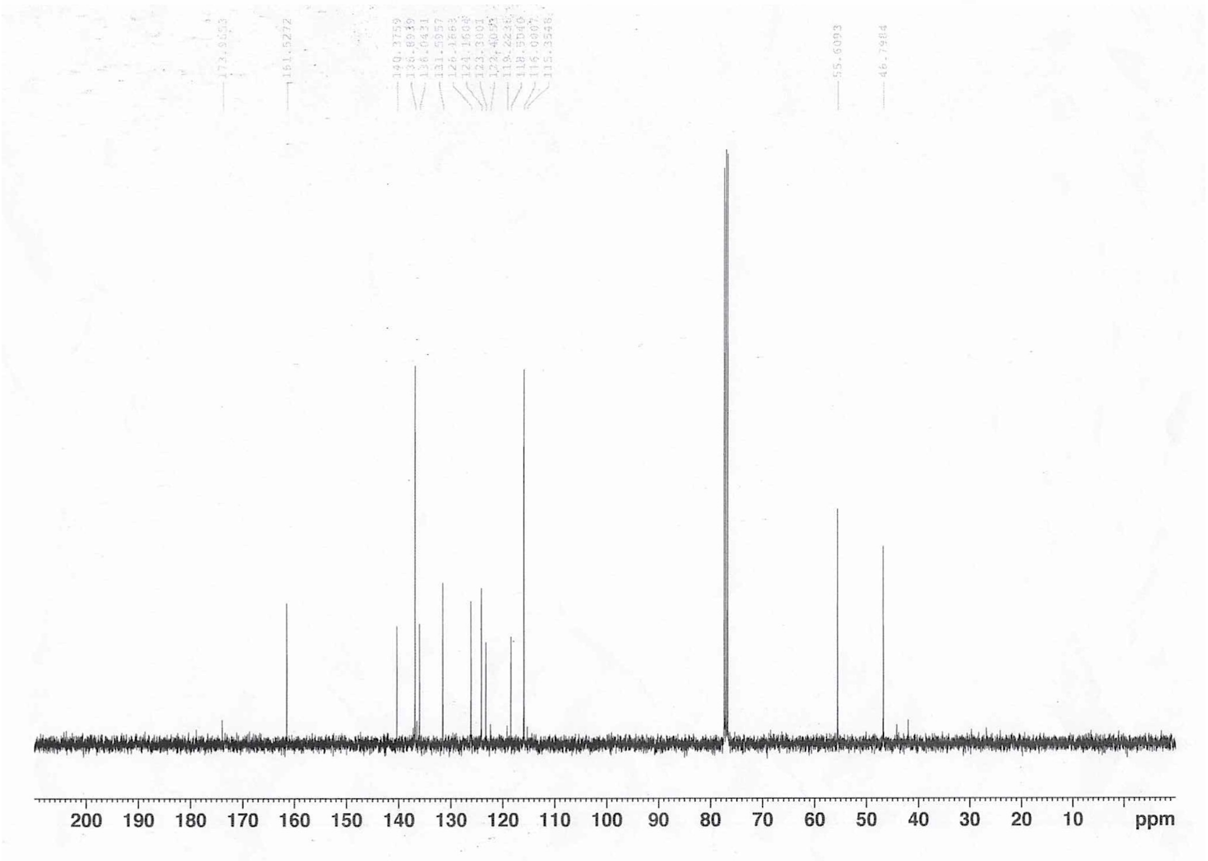

^{1}H NMR (400 MHz) and ^{13}C NMR (101 MHz) spectra of 9-methoxy-10*H*-thioxantheno[2,3-*d*][1,3]dioxol-10-one (**5a**) ($CDCl_3$)

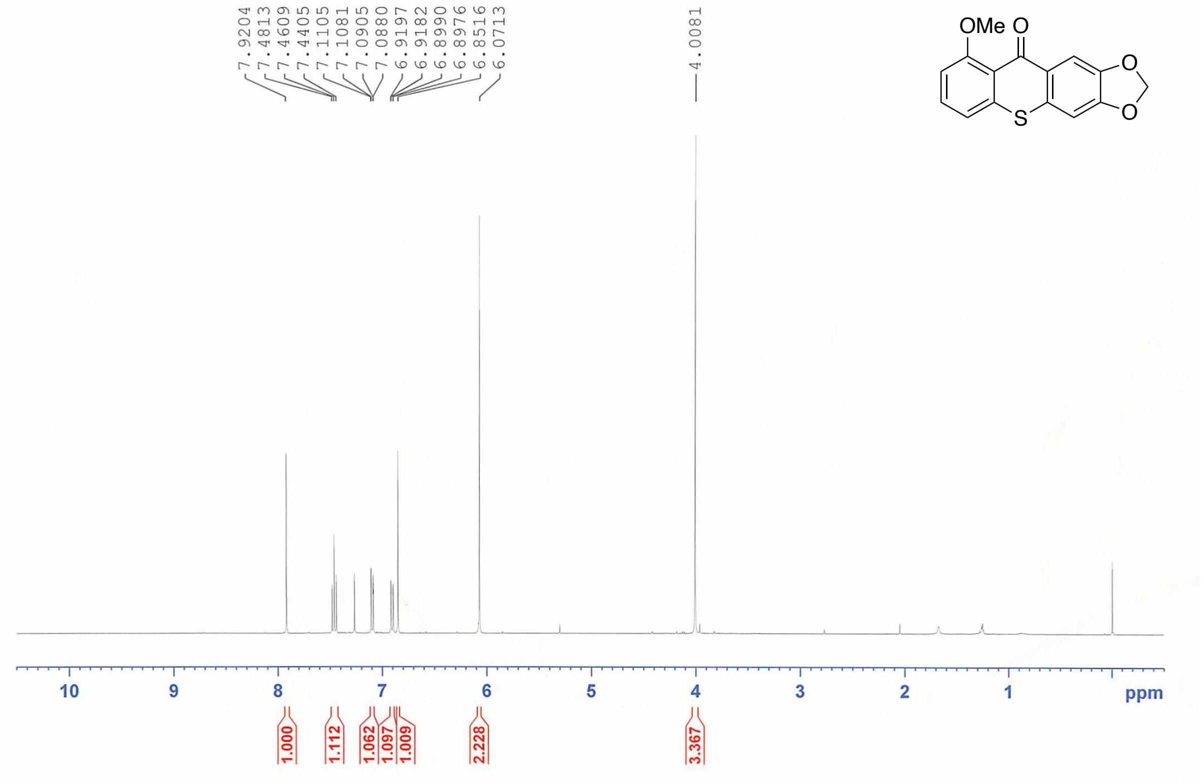

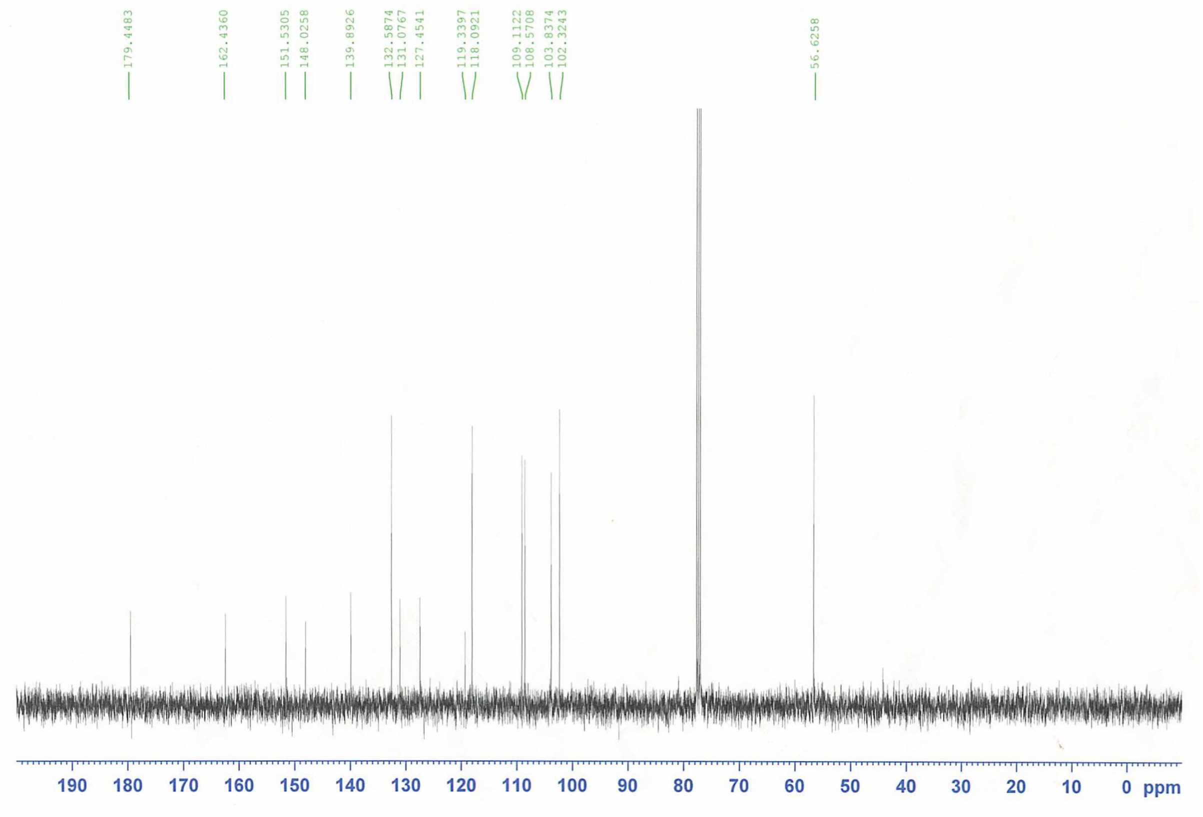

^{1}H NMR (400 MHz) and ^{13}C NMR (101 MHz) spectra of 1-(dimethylamino)-6,7-dimethyl-9*H*-thioxanthen-9-one (**5b**) ($CDCl_3$)

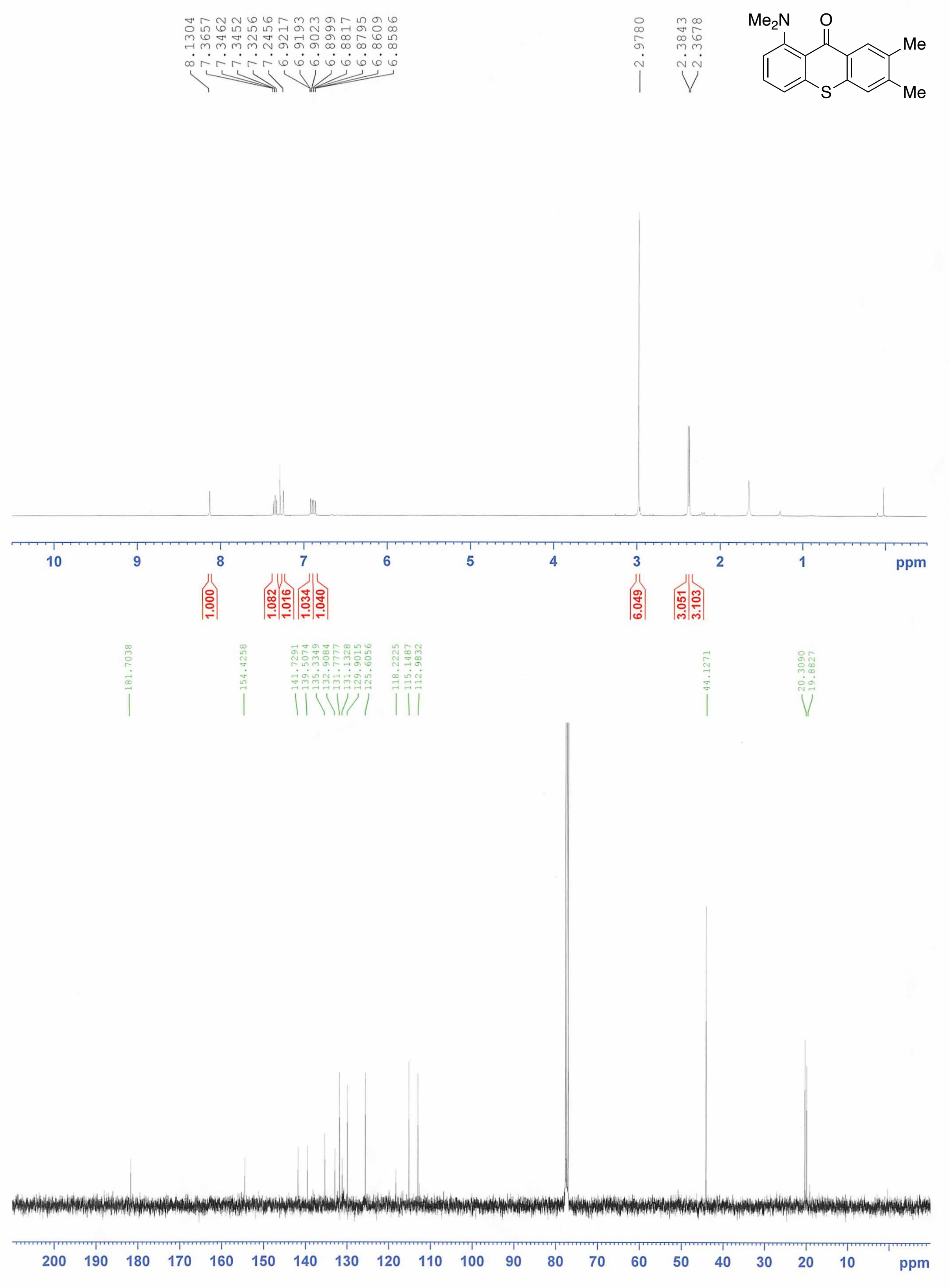

^{1}H NMR (400 MHz) and ^{13}C NMR (101 MHz) spectra of 1-bromo-6,7-dimethoxy-9*H*-thioxanthen-9-one (**5c**) ($CDCl_3$)

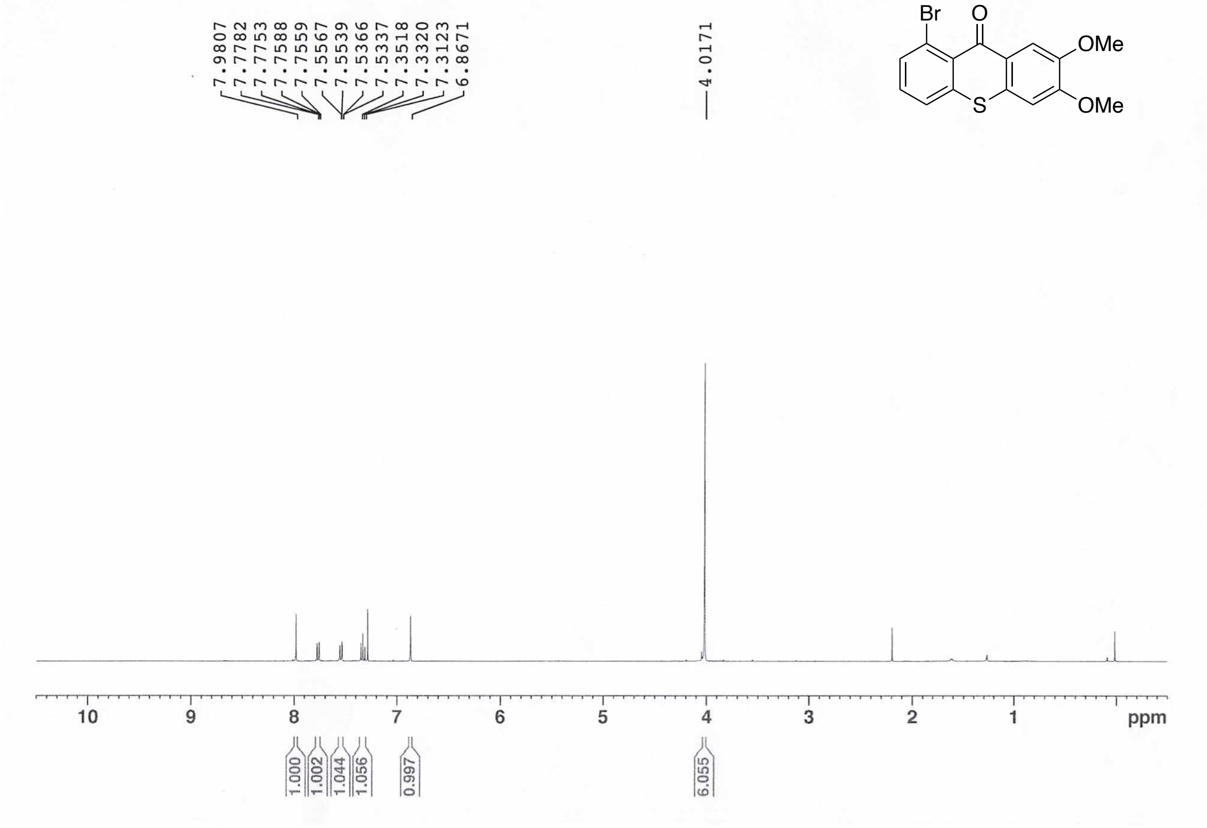

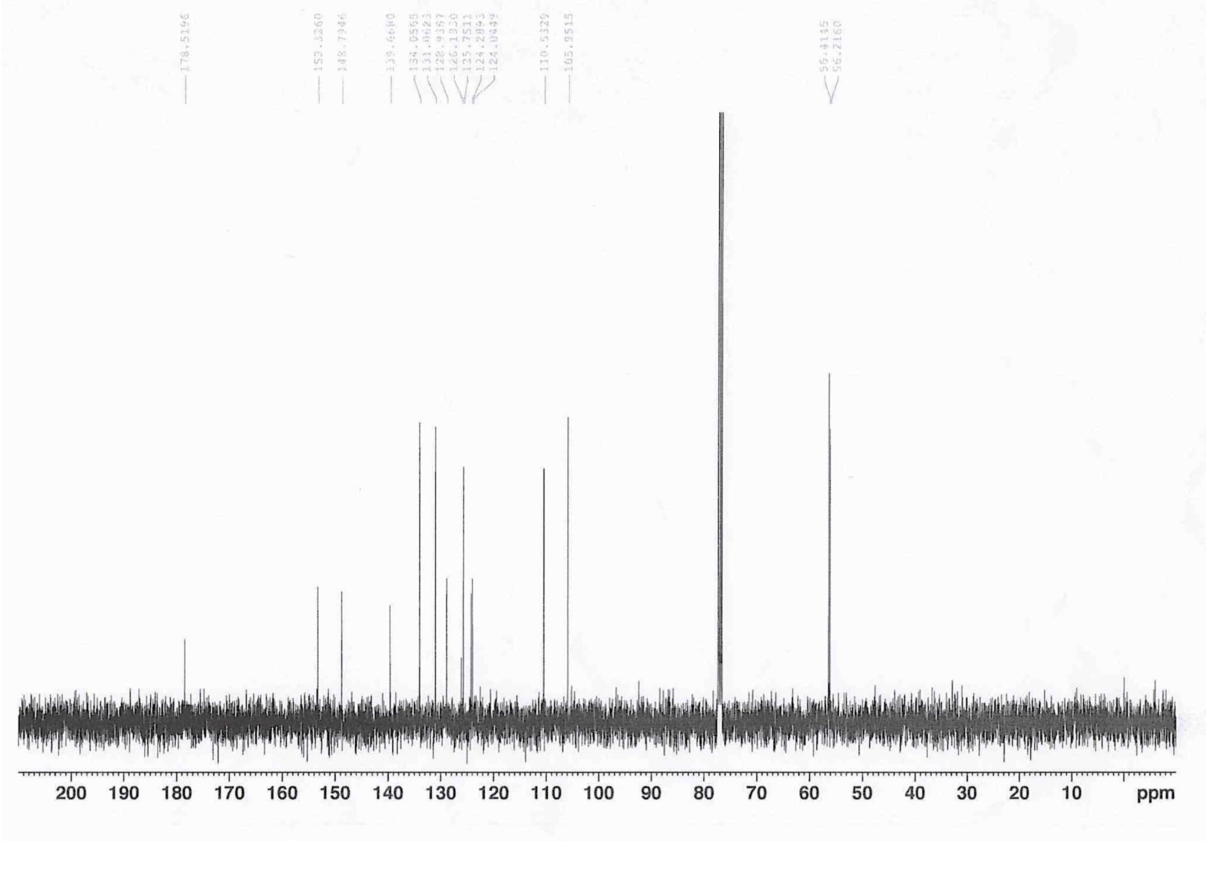

^{1}H NMR (400 MHz) and ^{13}C NMR (101 MHz) spectra of *N*-(1,8-dimethoxy-9*H*-thioxanthen-9-ylidene)-*N*-methyl-1-phenylmethanaminium trifluoromethanesulfonate (**4b**) ($CDCl_3$)

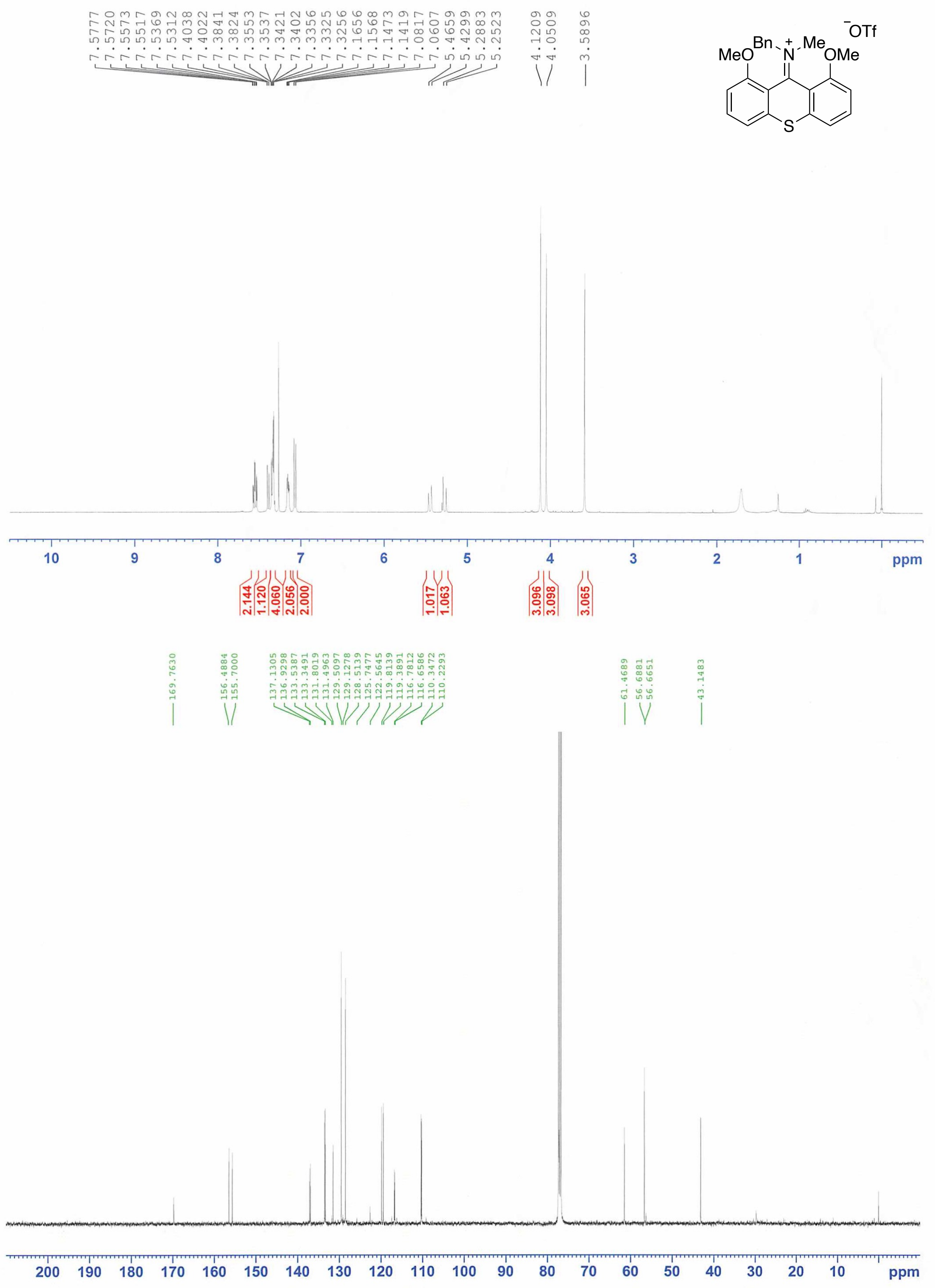

^{1}H NMR (400 MHz) and ^{13}C NMR (101 MHz) spectra of *N*-(1,8-dimethoxy-9*H*-thioxanthen-9-ylidene)-*N*-methylmethanaminium trifluoromethanesulfonate (**4c**) ($CDCl_3$)

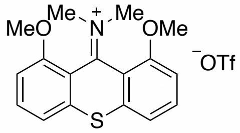

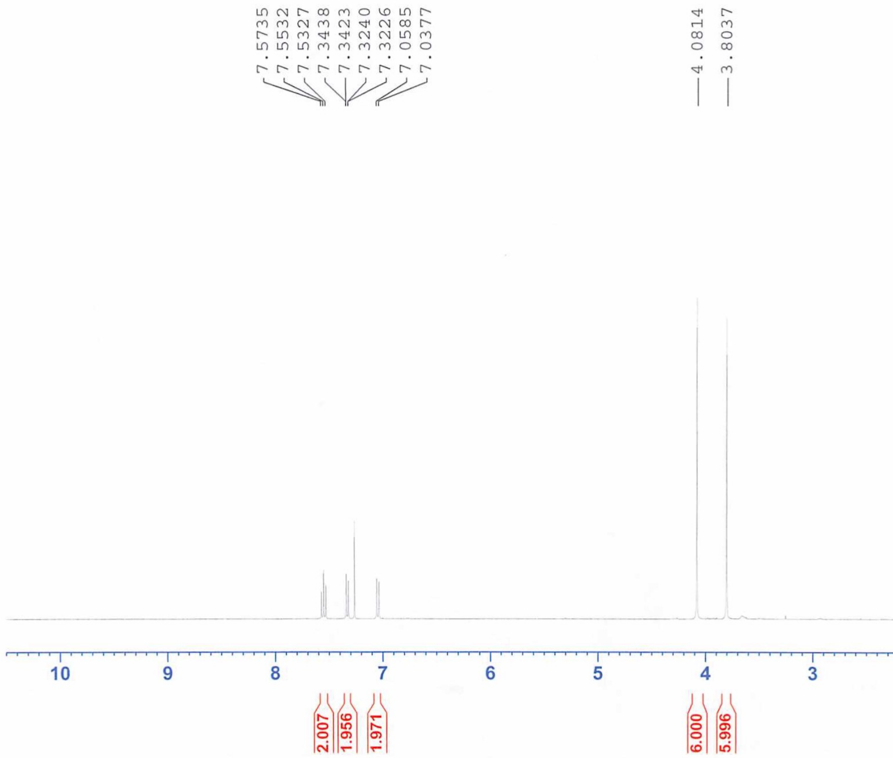

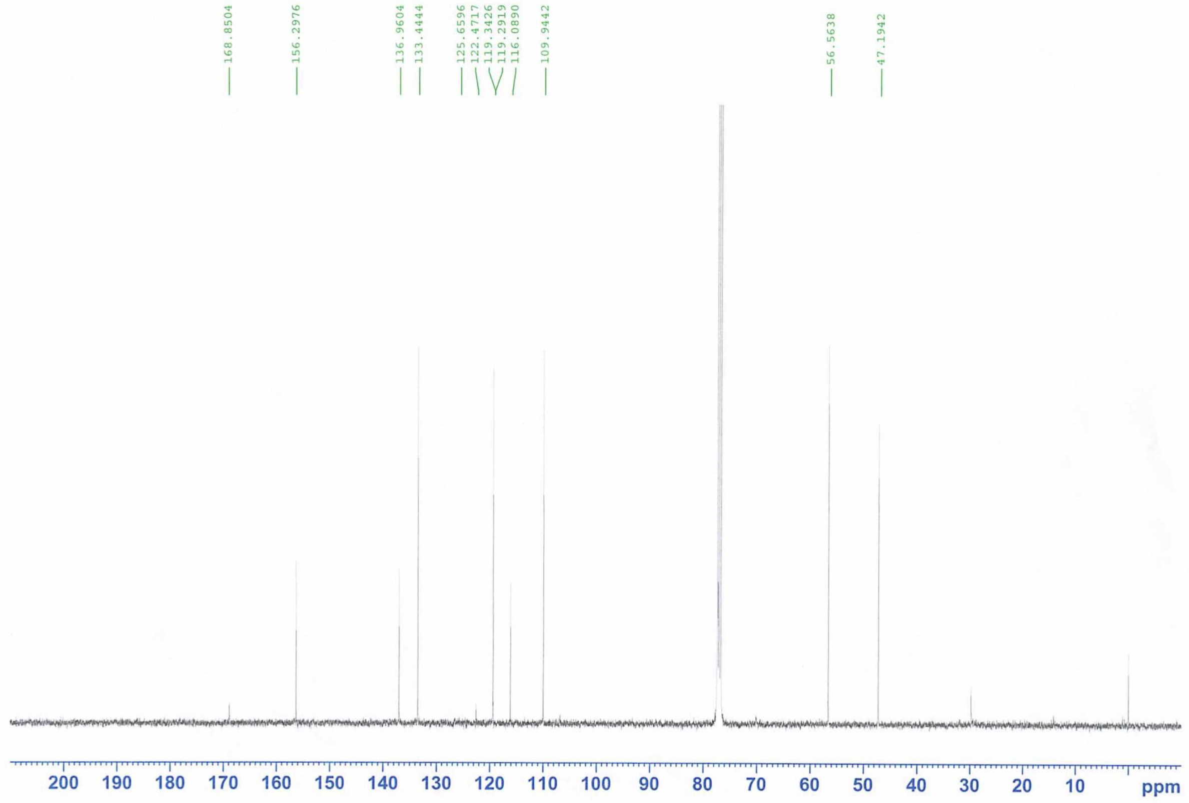

^{1}H NMR (400 MHz) and ^{13}C NMR (101 MHz) spectra of bis(dibenzo[*b*,*d*]furano)[5,4-*b*:5',4'-*d*]thiopyran-4-one (**3o**) ($CDCl_3$)

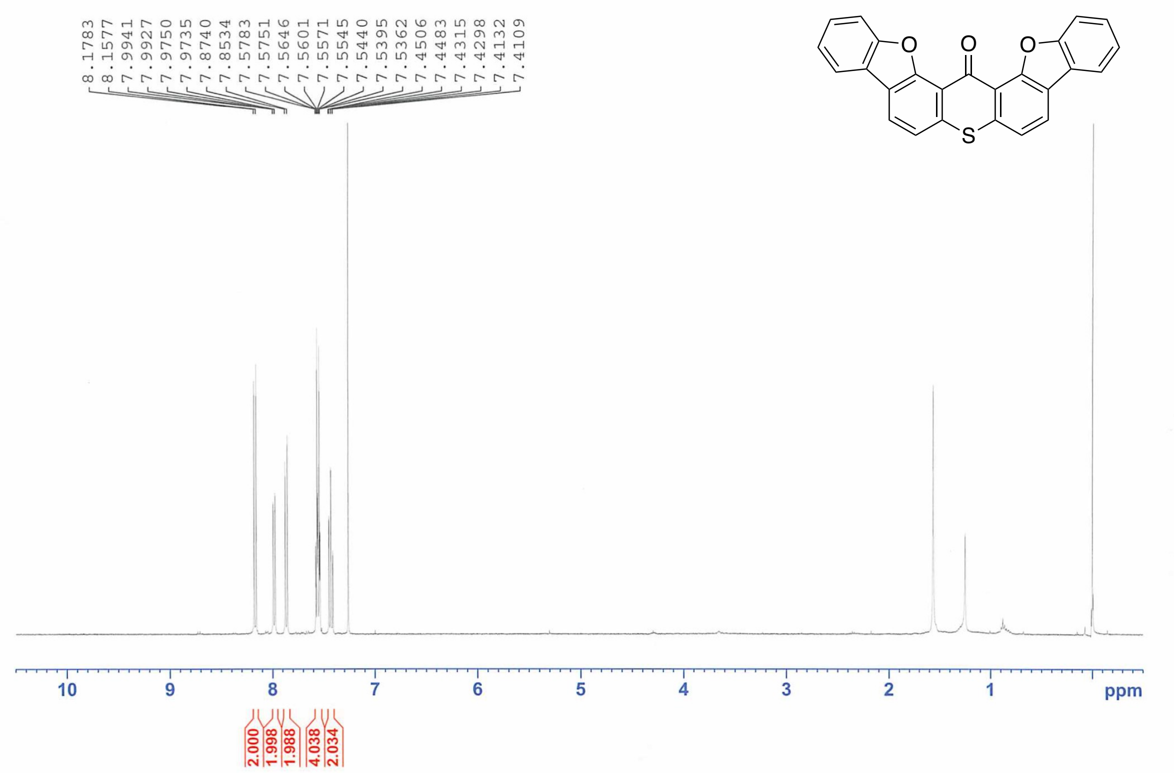

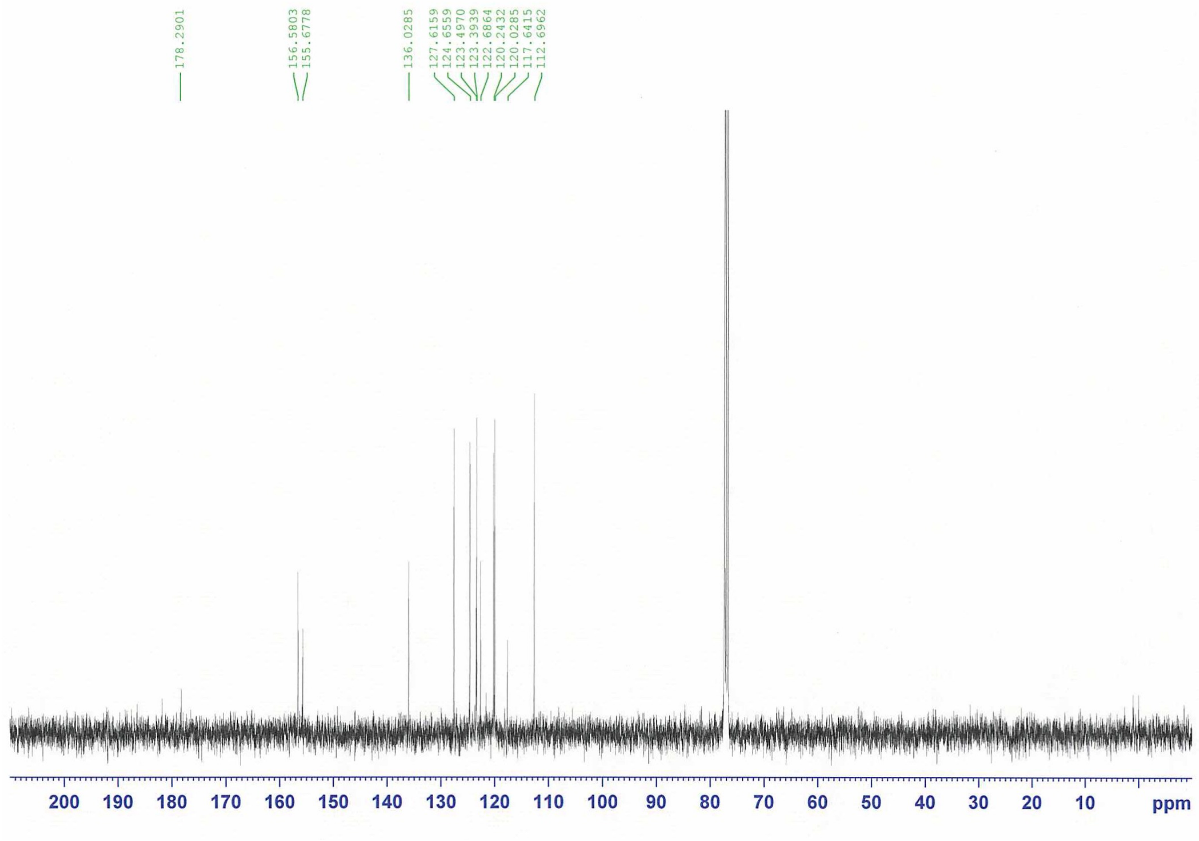

^{1}H NMR (400 MHz) and ^{13}C NMR (101 MHz) spectra of 2,3,6,7-tetramethoxy-9*H*-thioxanthen-9-one 10-oxide (**7**) ($CDCl_3$)

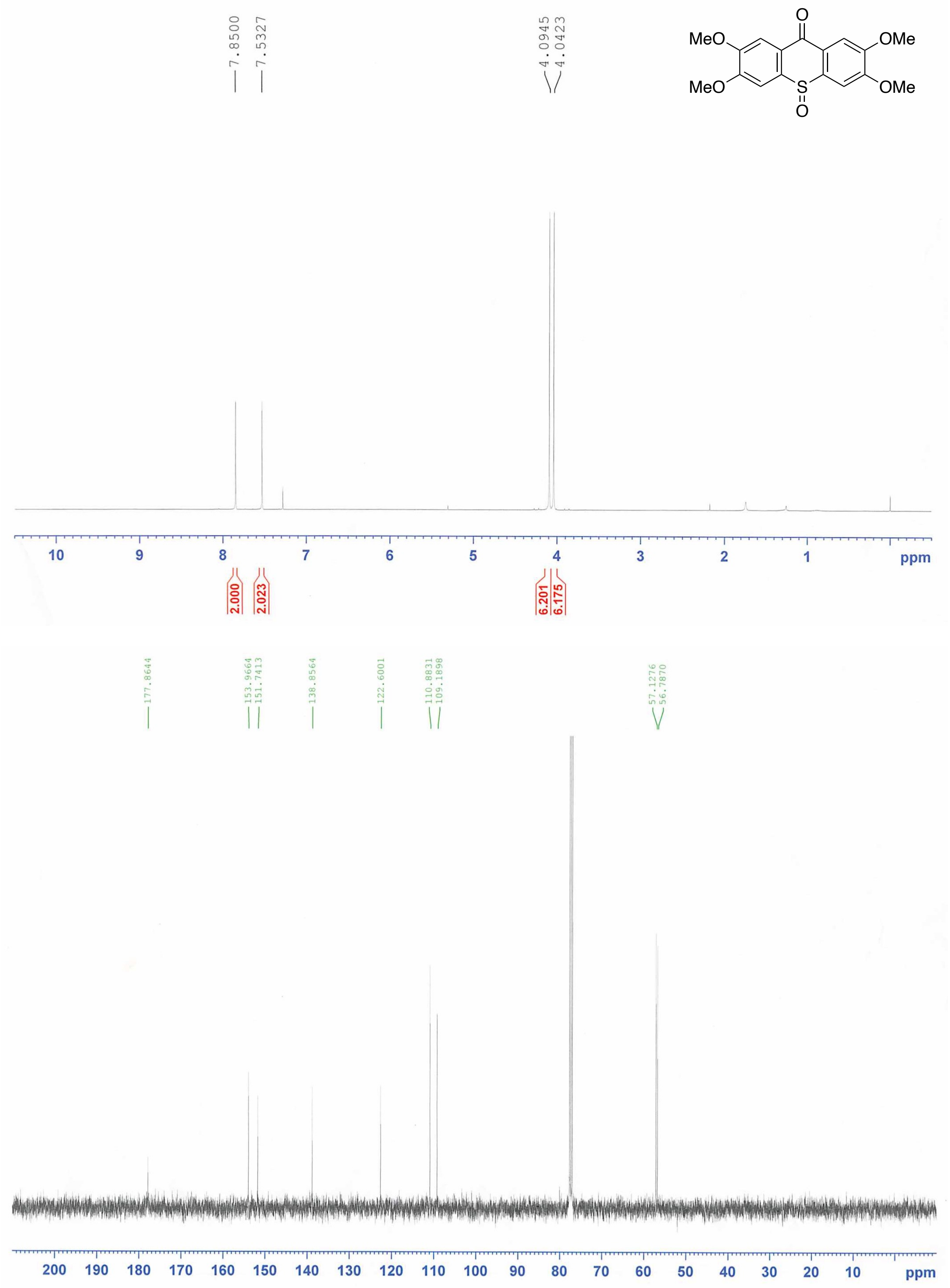

^{1}H NMR (400 MHz) and ^{13}C NMR (101 MHz) spectra of 2,3,6,7-tetramethoxy-9*H*-thioxanthen-9-one 10,10-dioxide (**8**) ($CDCl_3$)

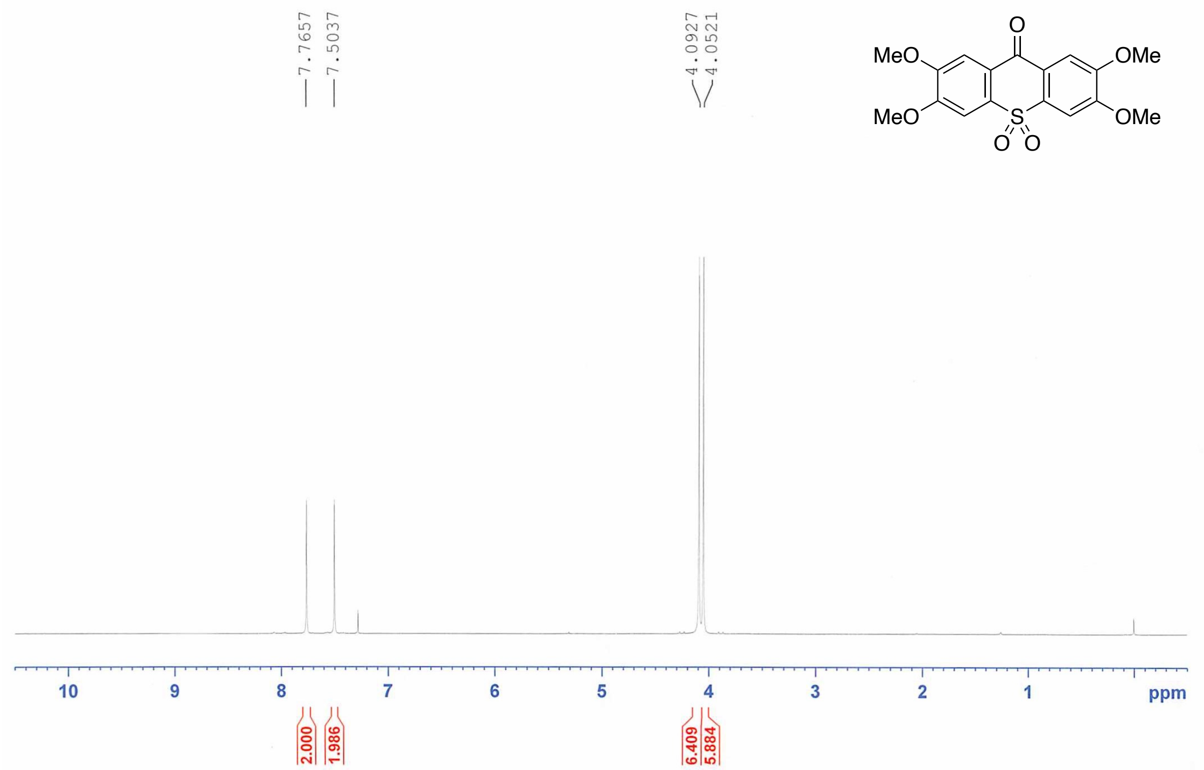

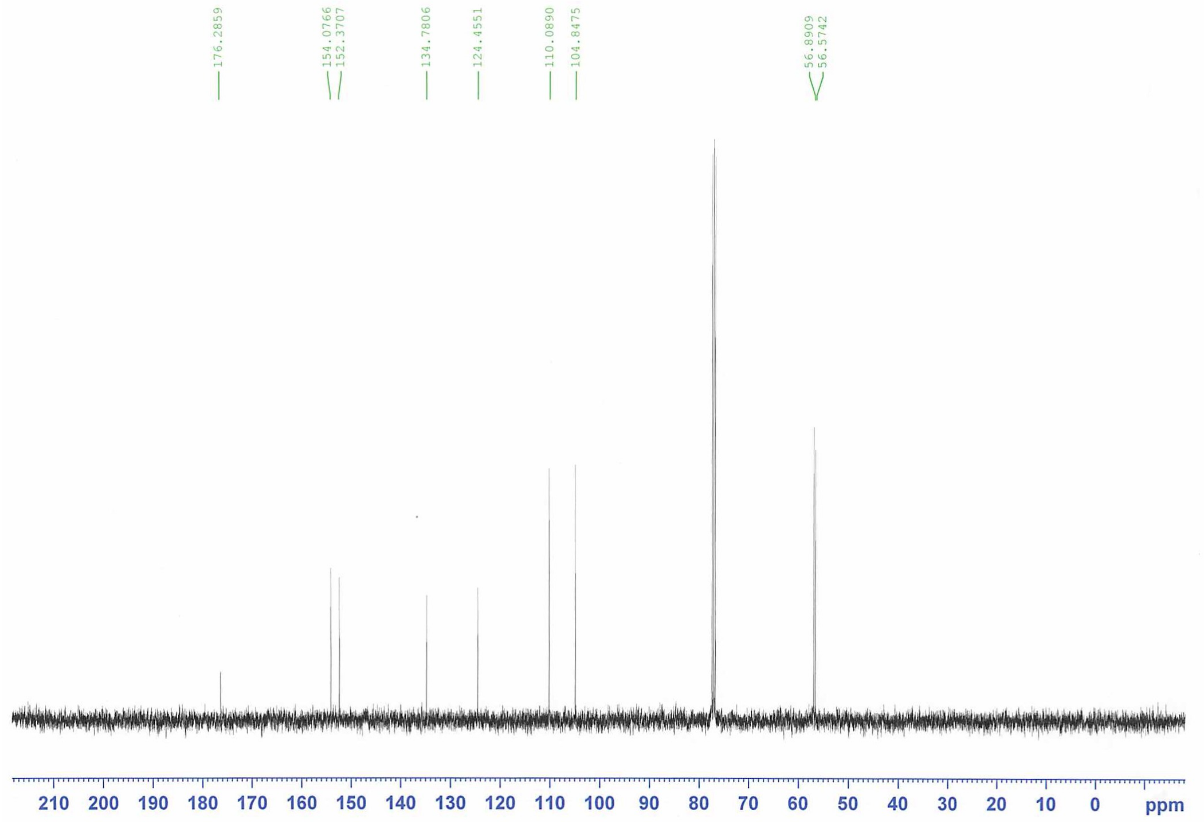

^{1}H NMR (400 MHz) and ^{13}C NMR (101 MHz) spectra of 2,3,6,7-tetramethoxy-9-(4-methoxyphenyl)thioxanthylium trifluoromethanesulfonate (**10**) ($CDCl_3$)

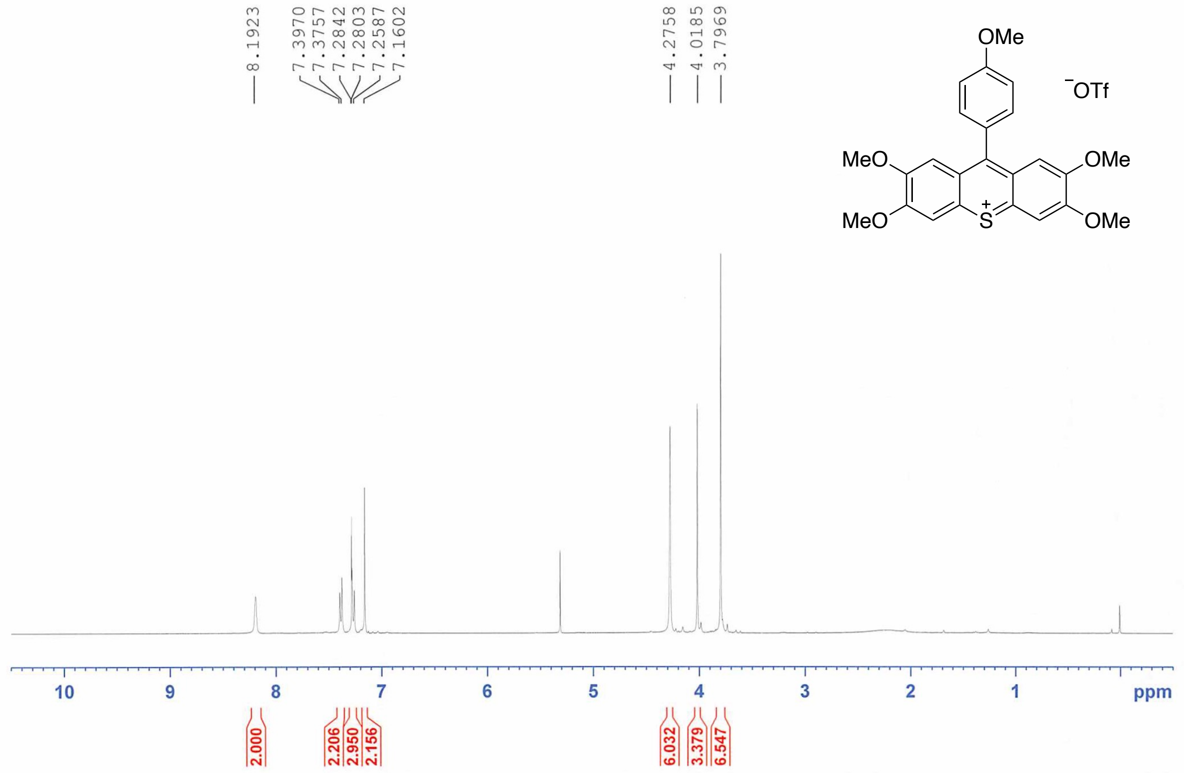

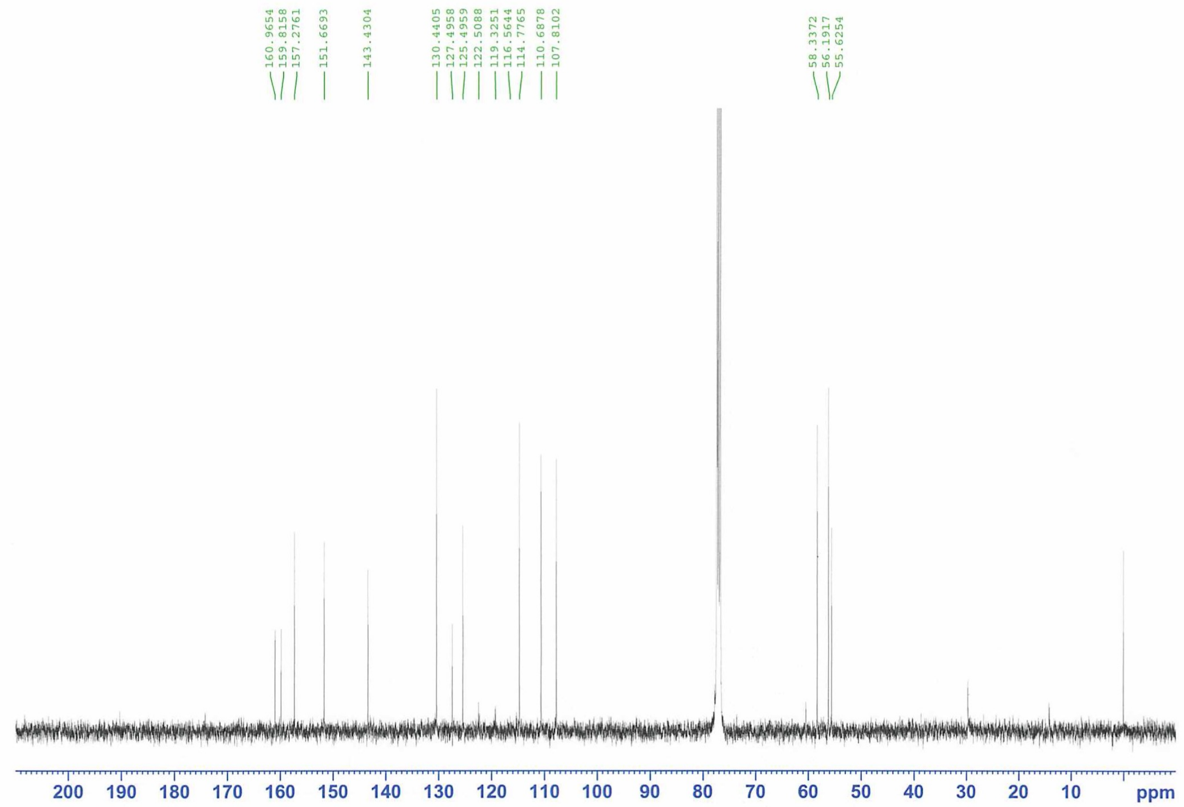

^{1}H NMR (400 MHz) and ^{13}C NMR (101 MHz) spectra of 4-iododibenzo[*b*,*d*]furan-3-ol ($CDCl_3$)

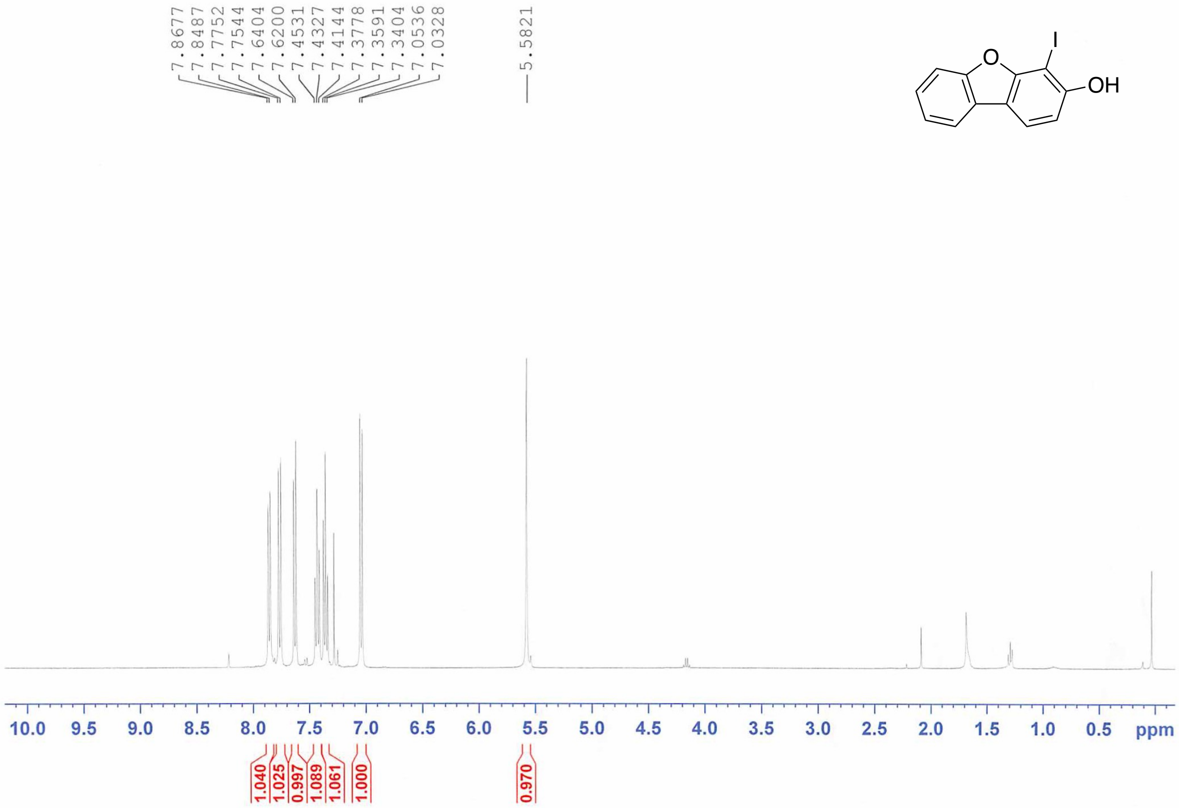

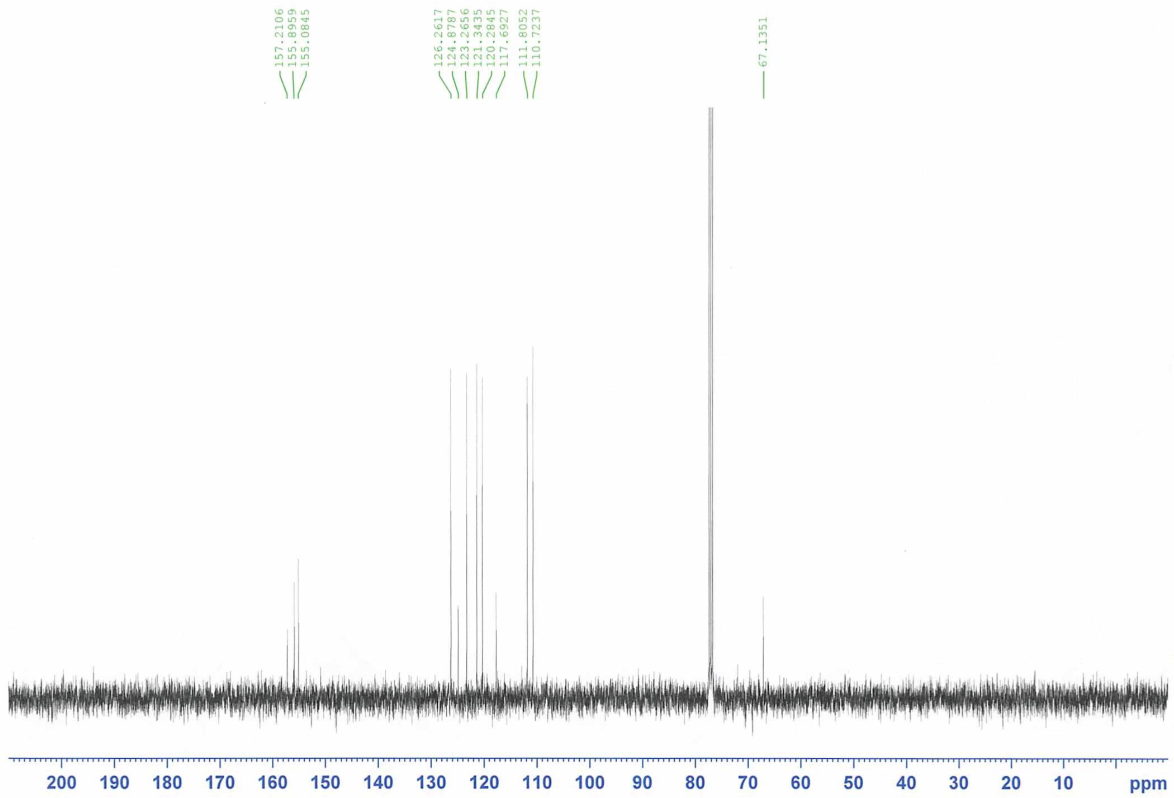

^{1}H NMR (400 MHz) and ^{13}C NMR (101 MHz) spectra of 4-(trimethylsilyl)dibenzo[*b*,*d*]furan-3-yl trifluoromethanesulfonate (**1p**) ($CDCl_3$)

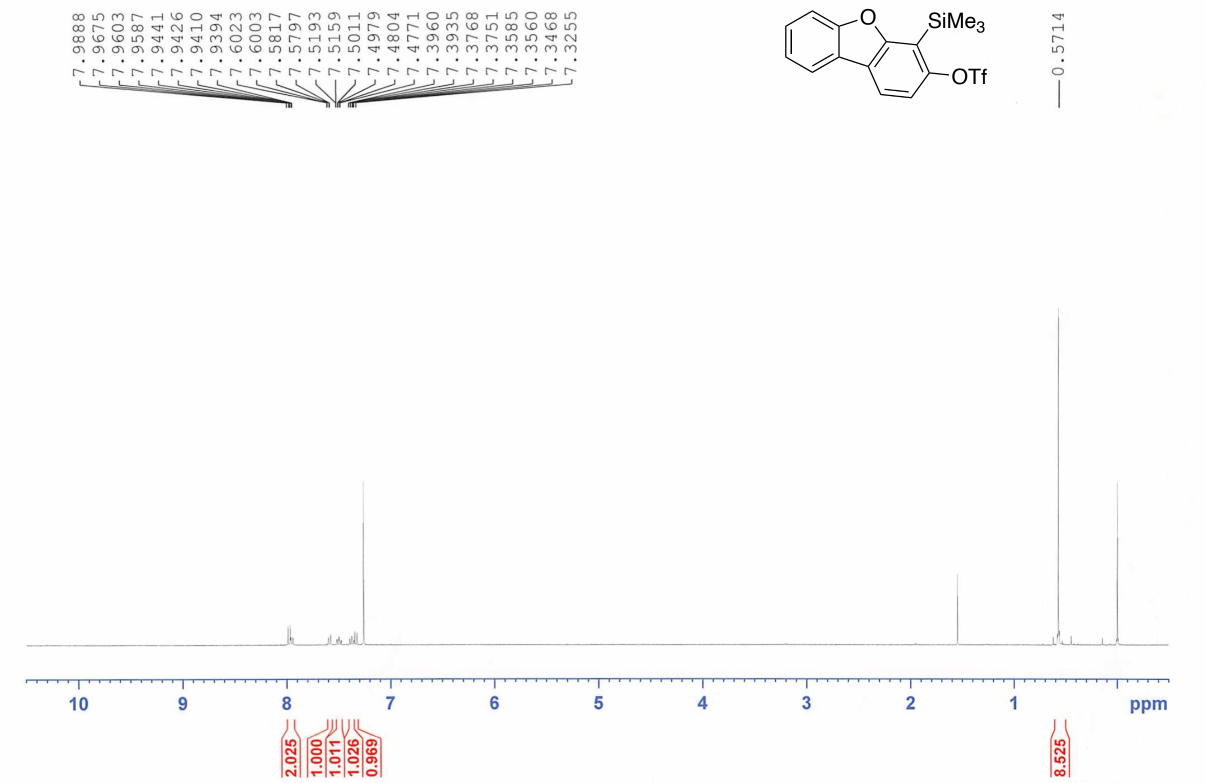

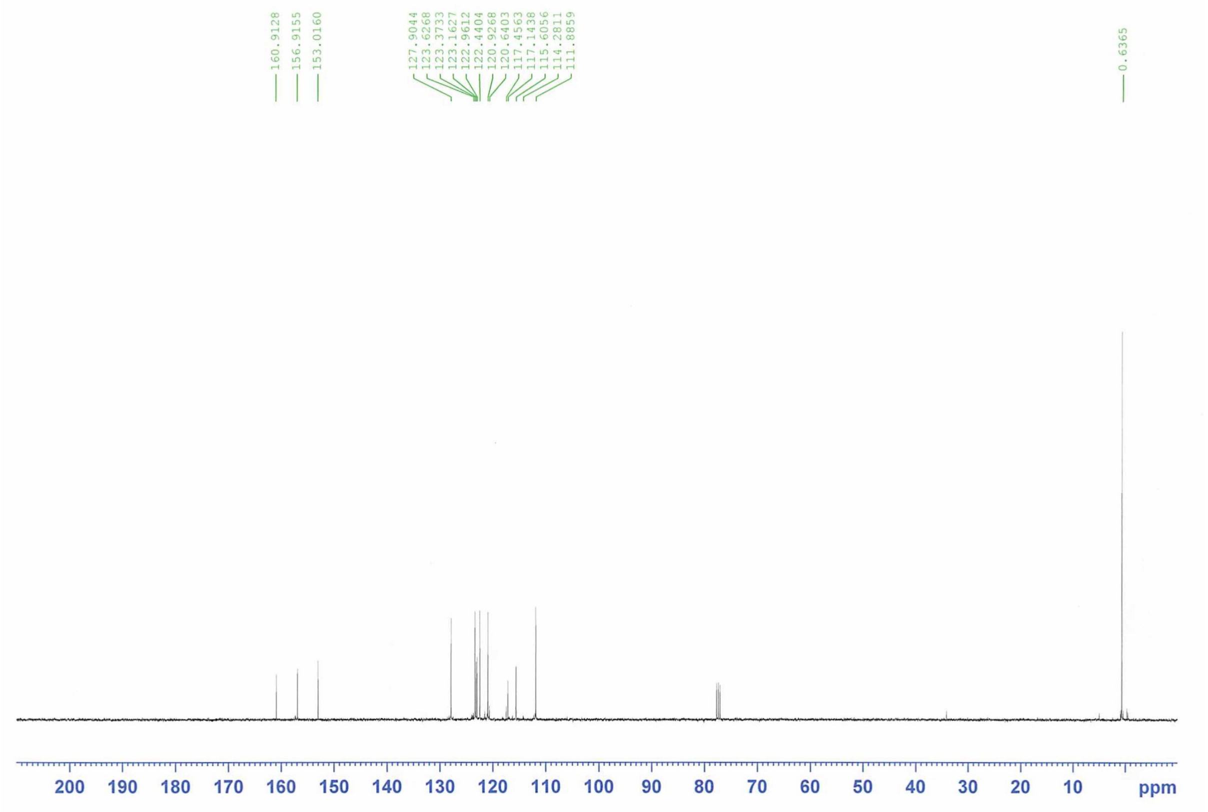

^{1}H NMR (400 MHz) and ^{13}C NMR (101 MHz) spectra of *O*,*O*-dibenzyl carbonothioate (**2a**) ($CDCl_3$)

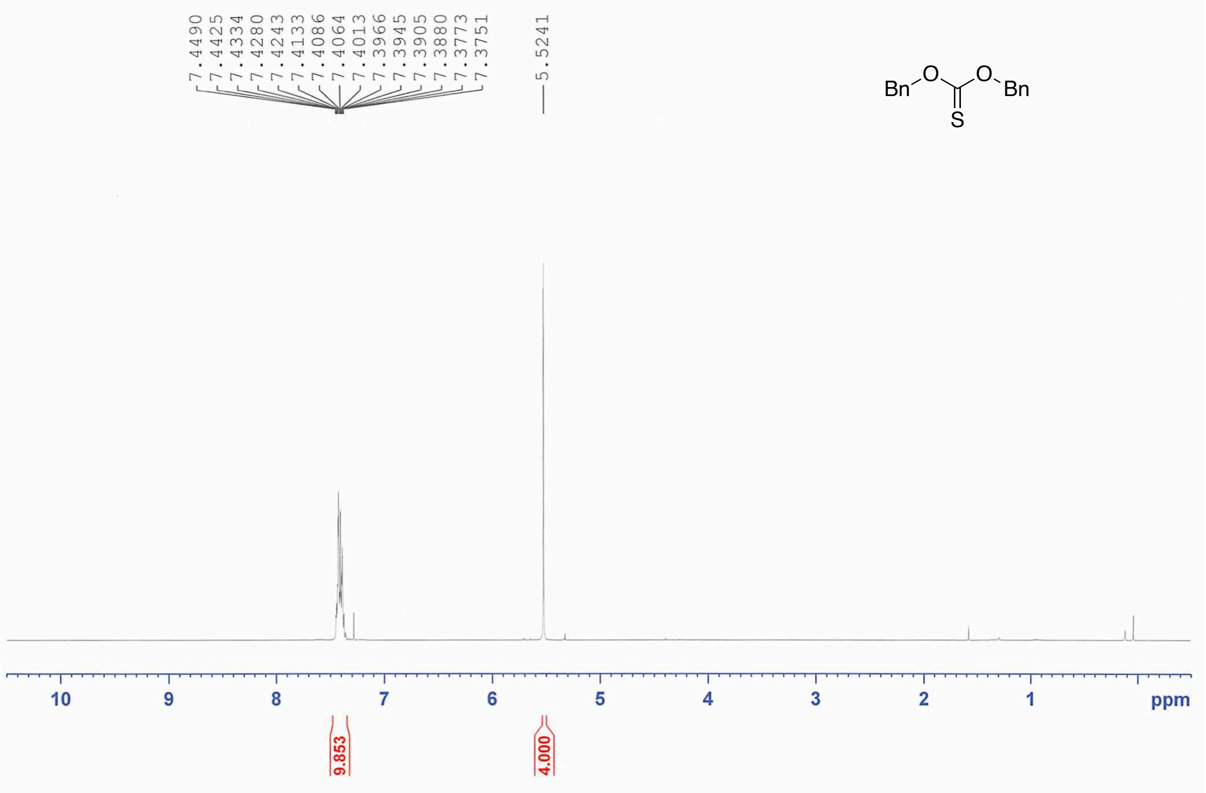

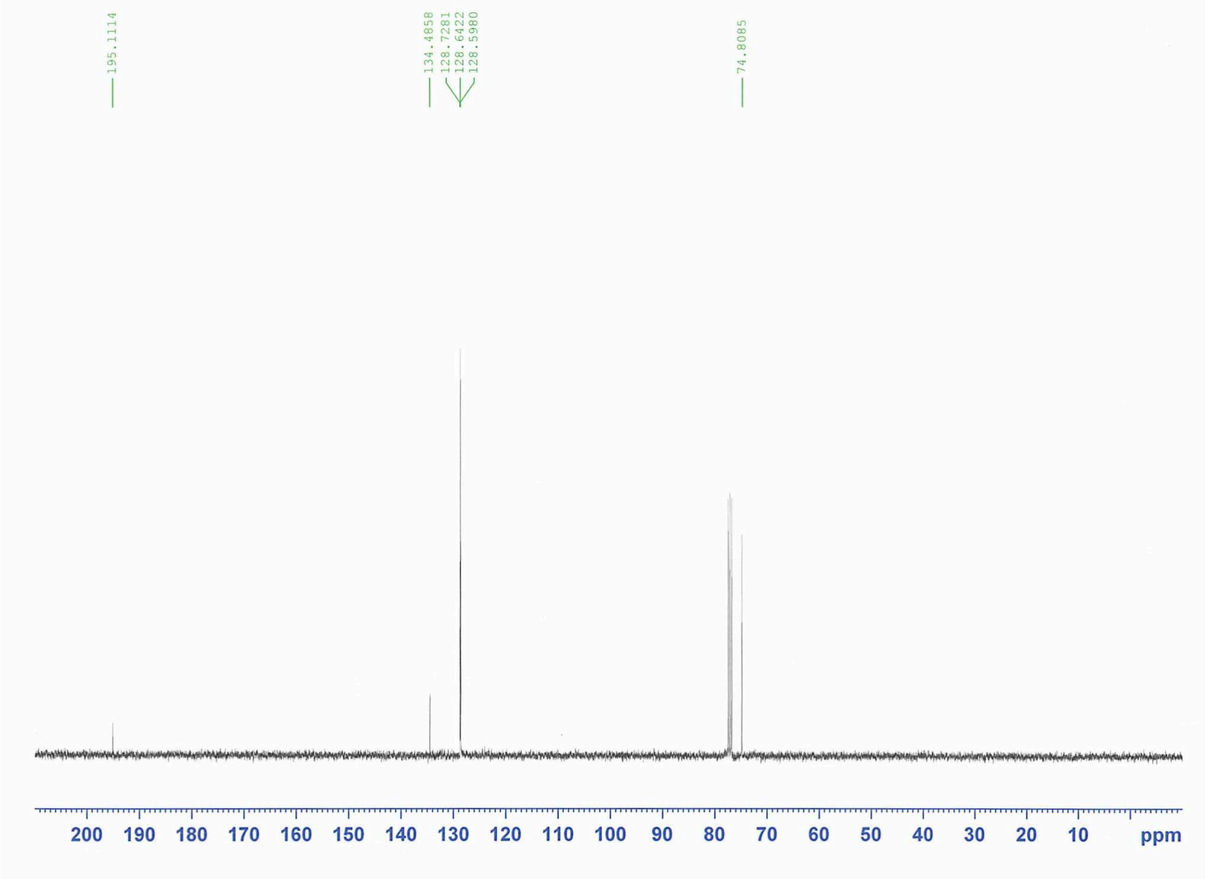

^{1}H NMR Spectra of Reported Compounds

^{1}H NMR (400 MHz) spectrum of 9*H*-thioxanthen-9-one (**3a**) ($CDCl_3$)

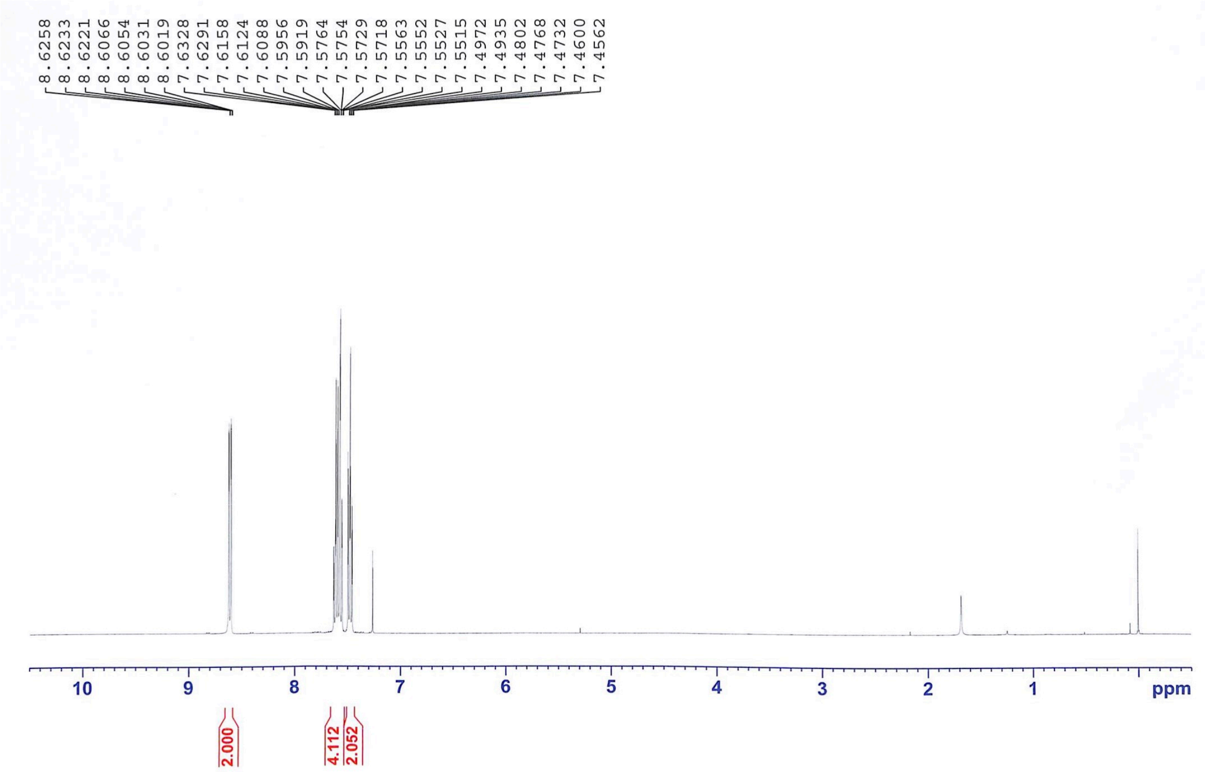

^{1}H NMR (400 MHz) spectrum of 1,8-dimethoxy-9*H*-thioxanthen-9-one (**3e**) ($CDCl_3$)

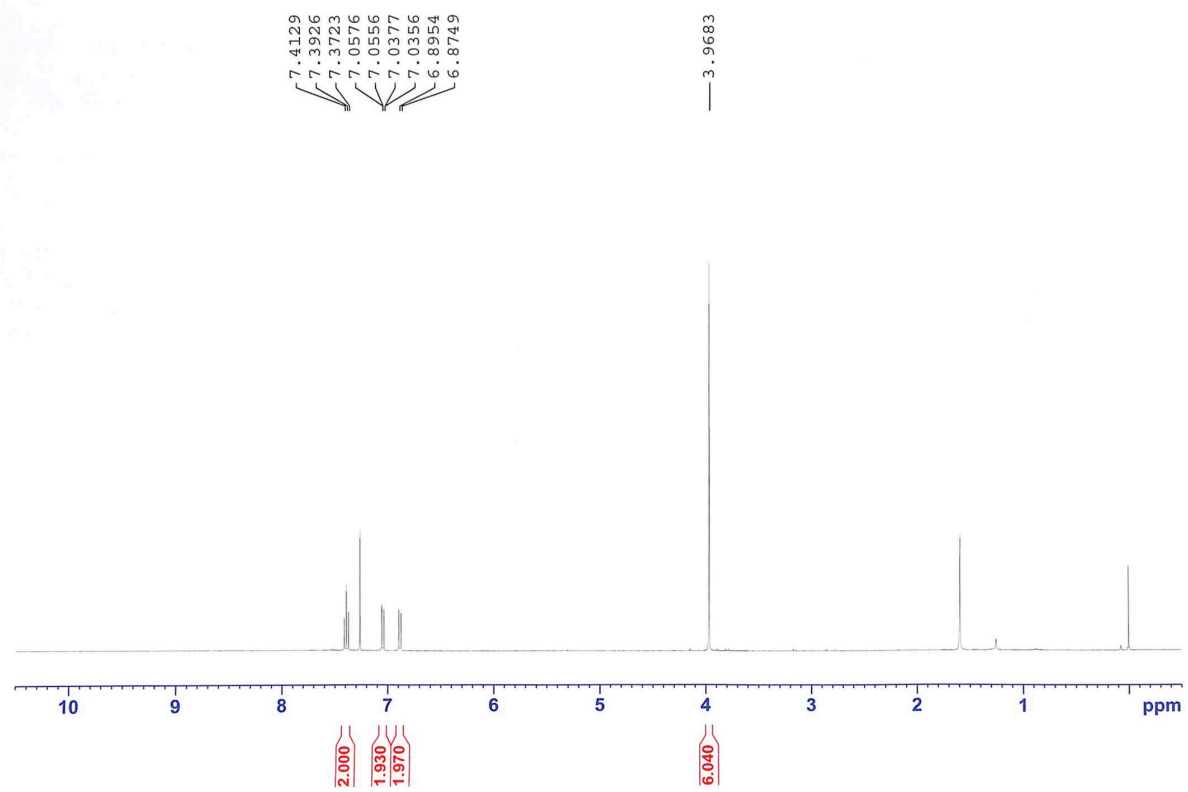

Supplement: Supplementary file 1 — ol4c04490_si_001.pdf [file ol4c04490_si_001.pdf]
